# Sequential and additive expression of miR-9 precursors control timing of neurogenesis

**DOI:** 10.1242/dev.200474

**Published:** 2022-10-03

**Authors:** Ximena Soto, Joshua Burton, Cerys S. Manning, Thomas Minchington, Robert Lea, Jessica Lee, Jochen Kursawe, Magnus Rattray, Nancy Papalopulu

**Affiliations:** ^1^ Division of Developmental Biology and Medicine, School of Medical Sciences, Faculty of Biology, Medicine and Health, The University of Manchester, Oxford Road, Manchester, M13 9PT, UK; ^2^ Institute of Science and Technology Austria (IST Austria), Am Campus 1, 3400 Klosterneuburg, Austria; ^3^ Discovery Department, Medicines Discovery Catapult, Block 35, Mereside, Alderley Park, Alderley Edge, Cheshire, SK10 4TG, UK; ^4^ School of Mathematics and Statistics, University of St Andrews, North Haugh, St Andrews, KY16 9SS, UK; ^5^ Division of Informatics, Imaging and Data Sciences, Faculty of Biology, Medicine and Health, The University of Manchester, Oxford Road, Manchester M13 9PT, UK

**Keywords:** pri-mir-9, miR-9, Neurogenesis, Zebrafish, Temporal control

## Abstract

MicroRNAs (miRs) have an important role in tuning dynamic gene expression. However, the mechanism by which they are quantitatively controlled is unknown. We show that the amount of mature miR-9, a key regulator of neuronal development, increases during zebrafish neurogenesis in a sharp stepwise manner. We characterize the spatiotemporal profile of seven distinct microRNA primary transcripts (pri-mir)-9s that produce the same mature miR-9 and show that they are sequentially expressed during hindbrain neurogenesis. Expression of late-onset pri-mir-9-1 is added on to, rather than replacing, the expression of early onset pri-mir-9-4 and -9-5 in single cells. CRISPR/Cas9 mutation of the late-onset pri-mir-9-1 prevents the developmental increase of mature miR-9, reduces late neuronal differentiation and fails to downregulate Her6 at late stages. Mathematical modelling shows that an adaptive network containing Her6 is insensitive to linear increases in miR-9 but responds to stepwise increases of miR-9. We suggest that a sharp stepwise increase of mature miR-9 is created by sequential and additive temporal activation of distinct loci. This may be a strategy to overcome adaptation and facilitate a transition of Her6 to a new dynamic regime or steady state.

## Teaser

**Highlighted Article:** Sharp stepwise increase of mature miR-9 is contributed by sequential-temporal activation of distinct loci; a strategy to overcome adaptation and facilitate a transition to a new state of Her6 dynamics/level.

## INTRODUCTION

MicroRNAs (miRs) are a class of small (∼22 nt) regulatory non-coding RNAs, which regulate gene expression at the post-transcriptional level. These small RNAs are processed from large microRNA primary transcripts (pri-mir) into 70∼90 nt precursors (pre-mir) before further splicing into ∼22 nt mature miR. miR-9 is a highly conserved miR that is expressed predominantly in the central nervous system (CNS) of vertebrates and plays a crucial role during CNS development. Specifically, previous work in *Xenopus*, zebrafish and mice has shown that miR-9 is essential for cell fate transitions during neurogenesis (Shibata et al., 2011; Coolen et al., 2013; Bonev et al., 2011, 2012). miR-9 post-transcriptionally targets many transcription factors that are involved in neural development such as FoxG1 (Shibata et al., 2008), Tlx (also known as Nr2e1; Zhao et al., 2009) and members of the Hes/Her helix-loop-helix family of transcription factors, including Hes1 in mouse and *Xenopus* (Bonev et al., 2011, 2012) and Her6/Her9 in zebrafish (Coolen et al., 2012; Galant et al., 2016; Soto et al., 2020; Leucht et al., 2008).

The Hes/Her family of proteins is expressed dynamically in an oscillatory manner at the ultradian timescale (Hirata et al., 2002; Shimojo et al., 2008). Hes/Her oscillations are achieved by a negative feedback loop, whereby Hes/Her proteins inhibit their own transcription coupled with a rapid turnover of protein and mRNA. Instability of both protein and mRNA allows for levels of the protein to fall, de-repression to occur and expression to resume, generating a cyclic pattern (Hirata et al., 2002; Novak and Tyson, 2008). Indeed, both mRNAs and proteins of Hes family genes are unstable: for example, in mice, the half-life of *Hes1* mRNA is ∼24 min, the Hes1 protein half-life is in the order of 22 min (Hirata et al., 2002) and the Her6 (Hes1 zebrafish orthologue) protein half-life is ∼12 min (Soto et al., 2020).

Instability of mRNA, as well as translation of protein, are partly controlled by miRs. Indeed, our previous work revealed that miR-9 regulation is important for controlling *Hes1* mRNA stability and allowing the oscillatory expression of Hes1 to emerge (Bonev et al., 2012; Goodfellow et al., 2014). We have recently shown that in zebrafish, the dynamics of Her6 protein expression switch from noisy to oscillatory and then to downregulation, and that these changes coincide temporally with the onset of miR-9 expression in the hindbrain (Soto et al., 2020). When the influence of miR-9 on *her6* is removed experimentally, Her6 expression does not evolve away from the ‘noisy’ regime and is not downregulated with a consequent reduction in progenitor differentiation. We have interpreted this to mean that the miR-9 input is necessary to constrain gene expression noise, enabling oscillations to occur and to be decoded by downstream genes, which in turn participate in downregulating Her6 as cells differentiate (Soto et al., 2020).

However, not only the presence of miR-9 but also the amount of miR-9 present is important, as too much or too little miR-9 can lead to dampening of Hes1 oscillations (Bonev et al., 2012; Goodfellow et al., 2014). Indeed, mathematical modelling showed that increasing miR-9 over time drives the Hes1 expression into different states (oscillatory or stable high/low) and that the amount of miR-9 present in the cell determines the length of time for which Hes1 oscillates, effectively timing the transition to differentiation (Phillips et al., 2016; Goodfellow et al., 2014). Together these findings support that Hes/Her dynamics and downregulation are sensitive to the amount of mature miR-9 present in the cell; however, the mechanism by which the miR-9 level is controlled is not known.

This question is complicated by the observation that vertebrates (and some invertebrates) possess multiple copies of the miR-9 gene at distinct loci, which are all capable of producing the same mature miR. For example, both human and mouse contain three copies of miR-9 (Rodriguez-Otero et al., 2011; Shibata et al., 2011) and frogs have four (Walker and Harland, 2008). Due to an additional round of whole-genome duplication (WGD) in teleost fish (Amores et al., 1998; Jaillon et al., 2004), zebrafish have seven paralogues of miR-9 (pri-mir-9-1 to pri-mir-9-7) (Chen et al., 2005).

One possibility is that different genomic loci contribute to miR-9 regulation in a qualitative way, with differential temporal and spatial specificity of mature miR-9 expression. Indeed, there is some limited evidence that these discrete copies of miR-9 are expressed differentially during development both temporally and spatially (Nepal et al., 2016; Tambalo et al., 2020). Another, and yet unexplored, possibility is that transcription from different loci may serve to control miR-9 quantitatively, that is to increase the amount of miR-9 in the cell and perhaps do so in a temporally controlled manner, thus contributing to the change of miR-9 levels that is necessary to drive a change in the dynamics of Hes/Her targets.

Here, we undertake a systematic study of pri-mir-9 expression in zebrafish that aims to address the likelihood of these distinct scenarios, with special attention to the possibility of a quantitative control mechanism. We show by *in situ* hybridization that the expression of miR-9 spreads from the forebrain to the hindbrain and increases quantitatively in the hindbrain between 24 and 48 h post-fertilisation (hpf). A detailed time course of the expression of all seven pri-mir-9 paralogues shows that they are all transcriptionally active, but exhibit subtle, yet distinct, temporal and spatial profiles. Focusing on a set of early- and late-expressed pri-mir-9s in the hindbrain (pri-mir-9-1, pri-mir-9-4 and pri-mir-9-5) by quantitative single molecule fluorescent *in situ* hybridisation at single cell level, we found that, surprisingly, in many cells, early and late pri-mir-9s were concurrently transcriptionally active such that the expression from late-activated pri-mir-9s is added on to the early ones. This is functionally significant as the specific mutation of the late pri-mir-9-1 selectively reduces neurons that normally differentiate late. Our mathematical modelling suggests that the sharp quantitative increase afforded by the deployment of additional transcriptional units, may facilitate the downregulation of Her6 at late time points. We found this to be consistent with a subtle but reproducible failure to downregulate Her6 at late stages when pri-mir-9-1 was specifically mutated. Taken together, although both quantitative and qualitative mechanisms may contribute to the decoding function of mature miR-9s, we found a previously unappreciated quantitative component in the deployment of pri-mir-9s, which is temporally controlled and in turn controls the evolution of Her6 dynamic expression over time.

## RESULTS

### Pri-mir-9s are expressed with differed temporal onset

miRs are derived from a duplex precursor and the -5p strand (‘guide’) is preferentially incorporated into an RNA-induced silencing complex (RISC) to exert its regulatory functions, while the complementary -3p strand (‘passenger’) is thought to be rapidly degraded. Indeed, for the mature miR-9 the miR-9-5p is designated as the ‘guide’ strand and its annotation is derived from the mature miR sequence being embedded in the 5′ stem of the miR-9 precursor.

**Fig. 1.**
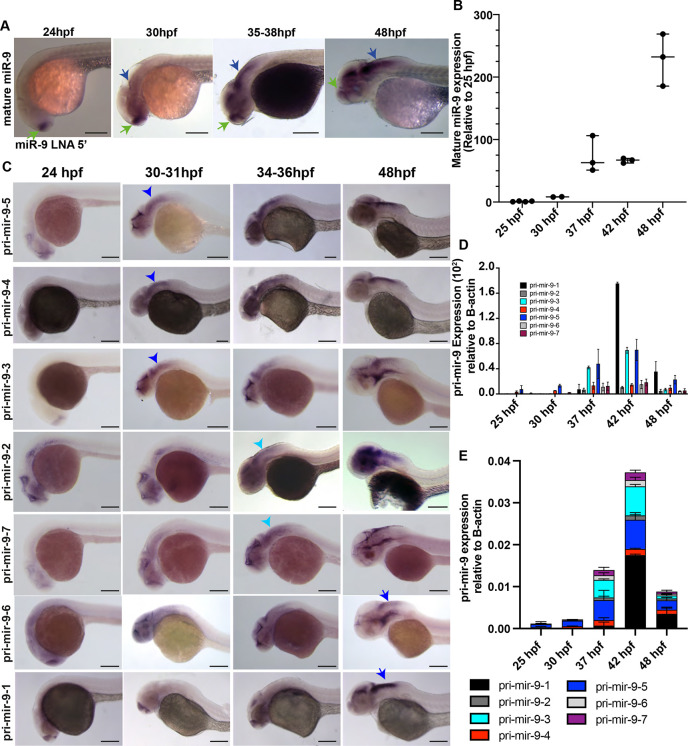
**Pri-mir-9 paralogues are expressed with different temporal onset.** (A) Representative example of chromogenic whole-mount *in situ* hybridization (WM-ISH) of miR-9 using miR-9 LNA 5′-Dig observed at different stages during development. Similar results can be observed in Soto et al. (2020). Longitudinal view, anterior to the left. Green arrow, forebrain expression; blue arrow, hindbrain expression. (B) Taqman RT-qPCR of mature miR-9 from dissected hindbrain at different stages of development, relative to 25 hpf. Horizontal bars indicate median with 95% confidence intervals. (C) Chromogenic WM-ISH of different pri-mir-9s using specific probes for each paralogue observed at different stages during development. Longitudinal view, anterior to the left. Blue arrowhead, expression in hindbrain at 30-31 hpf; light blue arrowhead, expression in hindbrain at 34-36 hpf; blue arrow, expression in hindbrain at 48 hpf. (D,E) SYBR green RT-qPCR relative quantification of the seven pri-mir-9s from dissected hindbrains at different stages of development. Quantification was normalised using β-actin. Data are mean±s.d. (B,D,E) *N*=3, each N contains a pool of 10 hindbrains. Scale bars: 200 μm.

To investigate the expression of the mature miR-9 (-5p strand) in zebrafish embryos, we first performed a whole-mount *in situ* hybridization (WM-ISH) for the mature miR-9 using a locked nucleic acid (LNA) probe. Mature miR-9 was detected only in the forebrain at 24 hpf (Fig. 1A), but at 30 hpf miR-9 was weakly observed in the midbrain and rhombomere (r) 1 of the hindbrain, maintaining high expression in the forebrain (Fig. 1A, 30 hpf). As development progressed, miR-9 expression increased in the hindbrain with steady high levels in the forebrain (Fig. 1A, 35-38 hpf; blue arrow). Later in development, levels in the hindbrain were further increased, while those in the forebrain were decreased (Fig. 1A, 48 hpf; blue arrow, hindbrain; green arrow, forebrain). These results show a temporally controlled increase of miR-9 expression along the brain/hindbrain axis as previously described in Soto et al. (2020).

To characterize the increase of expression in the hindbrain in a quantitative manner, we used quantitative real-time PCR (RT-qPCR) on dissected hindbrains from stages 25 hpf to 48 hpf. This analysis confirmed that there is upregulation in the time frame analysed. An initial low level of expression at 30 hpf was followed by a sharp upregulation at 37 hpf, which was maintained through to 42 hpf, undergoing a second sharp increase at 48 hpf (Fig. 1B).

In zebrafish, the mature miR-9 can be produced from seven paralogues of miR-9. The miR-9 paralogues occupy seven unique loci across the genome (GRCz11; Genome Reference Consortium Zebrafish Build 11) (Yates et al., 2020). With the exception of miR-9-3, which is located upstream of a long intergenic noncoding RNA (lincRNA), all miR-9 genes are intragenic, overlapping annotations of lincRNAs or proteins (Yates et al., 2020) (Fig. S1A,B). Our *in silico* analysis of previously published RNA-seq data shows differential temporal expression of six of the seven miR-9 paralogues hosts (White et al., 2017). It is also clear that upregulation of miR-9 host genes (and hence miR-9) coincides with a gradual decline in the expression of Her/Hes family gene expression, consistent with the idea that Her/Hes genes are major targets of miR-9 (Fig. S1C) (Bonev et al., 2011).

Previous work has revealed that the seven miR-9 zebrafish paralogues are expressed in the forebrain at early stages of neurogenesis; however, toward the end of embryonic neuronal differentiation they are also expressed in the hindbrain (Nepal et al., 2016). Little is known about the period spanning the peak of neurogenesis, when miR-9 controls downstream targets such as the ultradian oscillator Her6. To characterize the expression in greater spatiotemporal detail, particularly over regions of the hindbrain area in which Hes/Her target genes are expressed, we investigated the expression of all seven primary transcripts over a time period spanning the peak of neurogenesis, which occurs at 33 hpf (Lyons et al., 2003), using specific probes for each pri-mir-9 (Fig. S2; Materials and Methods, Molecular cloning).

We observed that all pri-mir-9s were first expressed in the forebrain (24 hpf) in a regional specific manner, which is not further characterised here. At 48 hpf they are all also expressed in the hindbrain (Fig. 1C, 24 and 48 hpf; Fig. S3A-C) consistent with previously described results (Nepal et al., 2016). Differential expression was evident in the intermediate stages. Specifically, pri-mir-9-3, -9-4 and -9-5 were expressed ahead of the others in the hindbrain (Fig. 1C, 30-31 hpf; blue arrowhead). At the peak of hindbrain neurogenesis (34-36 hpf), pri-mir-9-2 and -9-7 were upregulated, joining most of the pri-mir-9s that were highly expressed at this stage (Fig. 1C, 34-36 hpf). Pri-mir-9-1 and -9-6 were temporally delayed, showing hindbrain expression at 48 hpf, at which point all pri-mir-9 were fully expressed. Quantifying the expression with RT-qPCR confirmed that pri-mir-9-4 and -9-5 were expressed early and that expression of pri-mir-9-1 commenced relatively late, at 42 hpf (Fig. 1D,E). At 48 hpf, all pri-mir-9s had lower level of expression, although pri-mir-9-1 continued to be relatively high compared with the other pri-mir-9s (Fig. 1D,E). Overall, every pri-mir-9 was expressed in the CNS and exhibited a temporal progression.

### Expression of pri-mir-9s from distinct loci is additive and sequentially activated

To achieve a more detailed characterisation of expression, we selected three different primary transcripts based on: (1) the onset of their hindbrain temporal expression during development, earliest or latest; and (2) a phylogenetic analysis of sequence based on vertebrate evolutionary relationship performed by Alwin Prem Anand et al. (2018) to select representatives that are widely distributed in the phylogenetic tree. Thus, pri-mir-9-5 was selected as the earliest to be expressed in the hindbrain and belonging to clade I/subgroup I, pri-mir-9-4 as the earliest and belonging to clade II, and pri-mir-9-1 as the latest and belonging to clade I/subgroup II (Alwin Prem Anand et al., 2018) (Fig. S3D).

**Fig. 2.**
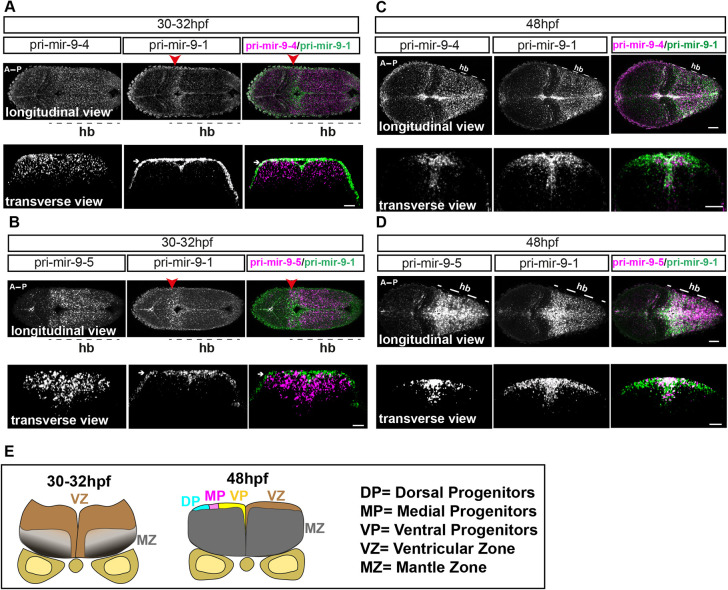
**Progressive additive expression of pri-mir-9s during development.** (A-D) Representative example of double fluorescent WM-ISH (WM-FISH) labelling of pri-mir-9-1/pri-mir-9-4 (A,C) and pri-mir-9-1/pri-mir-9-5 (B,D) in hindbrain (hb) from wild-type embryo observed at 30-32 hpf (A,B) and at 48 hpf (C,D). Transverse view was collected from hindbrain rhombomere 4/5. Longitudinal view was collected from embryos with anterior to the left and posterior to the right; images are maximum intensity projection; 5 μm thickness for 48 hpf embryos and 10 μm thickness for 30-32 hpf embryos. Merged images indicate pri-mir-9-4 or -9-5 in magenta and pri-mir-9-1 in green. White arrows indicate artefactual signal originated from the amplification step with FITC staining in the WM-FISH; red arrowheads indicate rhombomere 1 of the hindbrain. Pri-mir-9-1/pri-mir-9-4: longitudinal/30-32 hpf, *N*=3; transverse/30-32 hpf, *N*=3; longitudinal/48 hpf, *N*=4; transverse/48 hpf, *N*=8. Pri-mir-9-1/pri-mir-9-5: longitudinal/30-32 hpf, *N*=3; transverse/30-32 hpf, *N*=4; longitudinal/48 hpf, *N*=4; transverse/48 hpf, *N*=5. (E) Schematic of transverse section from zebrafish hindbrain at the level of the otic vesicle for 30-32 hpf and 48 hpf. A, anterior; MZ, mantle zone; P, posterior; VZ, ventricular zone. Within the VZ there are dorsal progenitors (DP), medial progenitors (MP) and ventral progenitors (VP). Scale bars: 30 µm.

Double whole-mount fluorescent *in situ* hybridization (WM-FISH) for pri-mir-9-1/pri-mir-9-4 and pri-mir-9-1/pri-mir-9-5 performed on stage 30-32 hpf embryos revealed expression of pri-mir-9-4 and pri-mir-9-5 along the anterior-posterior (A-P) hindbrain axis, whereas the expression of pri-mir-9-1 at this early stage was limited to the region of the anterior hindbrain corresponding to r1 (Fig. 2A; red arrowhead). A transverse view at mid-hindbrain (r4) reveals expression of pri-mir-9-4 and pri-mir-9-5 within the ventricular zone (VZ; Fig. 2A,B,E), indicating that pri-mir-9s are expressed in the region where most of the progenitors are found (Lyons et al., 2003; Tambalo et al., 2020). Pri-mir-9-1 staining shows an artefactual surface expression, as indicated with white arrow in transverse view at 30-32 hpf (Fig. 2A,B); this is because of the WM-FISH detection method.

We repeated this analysis at 48 hpf to examine whether the late expression of pri-mir-9-1 is cumulative with pri-mir-9-4 and pri-mir-9-5 or spatially distinct. Double WM-FISH of pri-mir-9-1 with pri-mir-9-4 or pri-mir-9-5 revealed overlapping expression of the primary transcripts in both longitudinal and transverse views (Fig. 2C,D). In addition, some distinct expression was observed in transverse views in that pri-mir-9-1 was more broadly expressed toward the dorsal progenitor region (Fig. 2C-E) when compared with pri-mir-9-4 and pri-mir-9-5.

### Mature miR-9 accumulates in single cells by overlapping expression of distinct loci primary transcripts

**Fig. 3.**
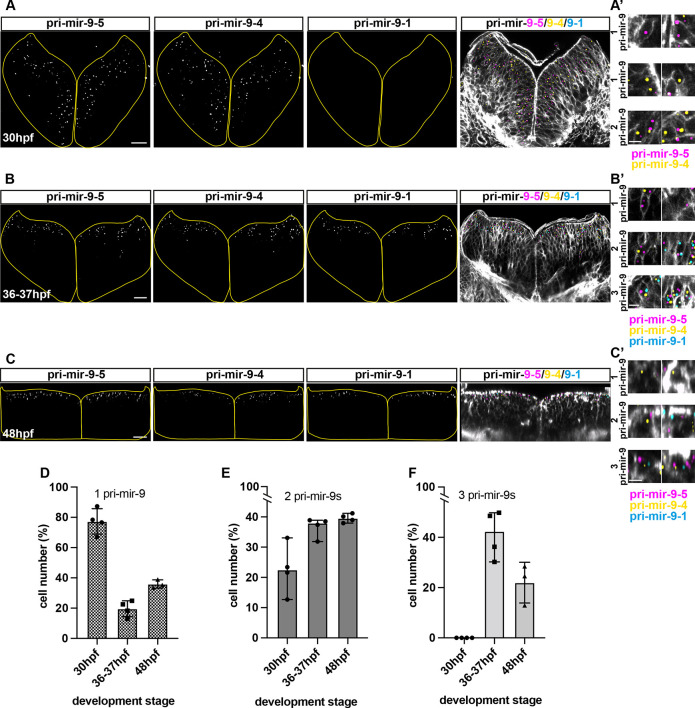
**Mature miR-9 expression in a cell is contributed by overlapping activation of distinct miR-9 loci.** (A-C) Representative example of transverse view from triple whole-mount smiFISH, labelling active transcriptional sites for pri-mir-9-5, -9-4 and -9-1 (from left to right) combined with cell boundary staining (Phalloidin-Alexa Fluor 488) in hindbrain from wild-type embryo at 30 hpf (A), 36-37 hpf (B) and 48 hpf (C). Merged images show pri-mir-9-5 in magenta, pri-mir-9-4 in yellow, pri-mir-9-1 in cyan and membrane in grey. (A′-C′) Increased magnification of representative images to show single cells expressing any single pri-mir-9 (1 pri-mir-9), any two different pri-mir-9 (2 pri-mir-9) and the three different pri-mir-9 (3 pri-mir-9). (D-F) Percentage of cells expressing any single pri-mir-9 (D), any two different pri-miR-9 (E) and three different pri-mir-9 (F) relative to total number of cells positive for the precursors (30 hpf, *N*=4; 37-37 hpf, *N*=4; 48 hpf, *N*=3). Data are median with 95% confidence interval. Scale bars: 20 µm (A-C); 5 µm (A′-C′).

For overlapping expression to contribute to the total levels of mature miR-9 in a cell, early and late pri-mir-9s would need to be expressed in the same cells. Thus, we investigated pri-mir-9 expression at the single-cell level, using triple WM-smiFISH for pri-mir-9-1, -9-4 smiFISH (single-molecule inexpensive fluorescent *in situ* hybridization) and -9-5 to detect nascent transcription sites, and Phalloidin staining to reveal cell boundaries. At 30 hpf we observed that most cells expressed only one miR-9 primary transcript, pri-mir-9-4 or pri-mir-9-5, while a small proportion expressed both and none expressed pri-mir-9-1 (Fig. 3A,D-F). By contrast, at 36-37 hpf and 48 hpf (Fig. 3B,C), the number of cells that expressed one pri-mir-9 decreased and, correspondingly, the number that expressed two or three pri-mir-9s increased. The most striking increase was observed in the number of cells that co-express three pri-mir-9s at 36-37 hpf, which was because of the onset of transcription of pri-mir-9-1 in the same cells that expressed pri-mir-9-4 and -9-5. This finding suggests that, in many hindbrain cells, the late expression of pri-mir-9-1 is added to the earlier expression of pri-mir-9-4 and -9-5.

### Medial and dorsal progenitors maintain concurrent expression of miR-9 primary transcripts at late neurogenesis

**Fig. 4.**
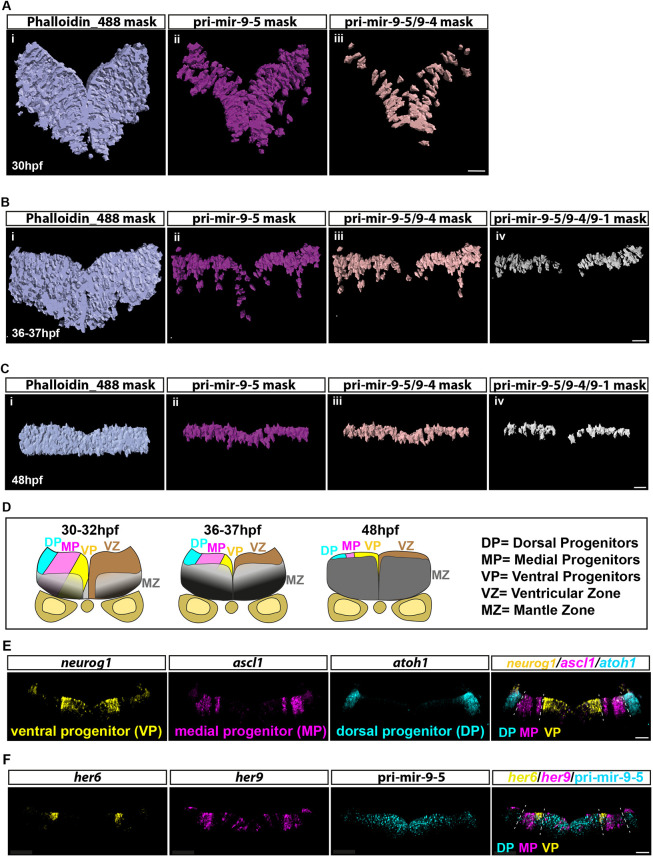
**Concurrent expression of miR-9 precursors in dorsal and medial progenitors.** (A-C) Mask representing segmented cells obtained from confocal images in Fig. 3. Using Imaris software, the cell segmentation was performed based on the membrane marker, Phalloidin-AF488, and the spot tool allowed us to count active transcriptional sites for pri-mir-9-5, -9-4 and -9-1. From left to right we visualize the mask showing all segmented cells present in the transverse view of the hindbrain (i, light blue), segmented cells that express pri-mir-9-5 (ii, magenta), both pri-mir-9-5 and -9-4 (iii, light pink) and all three pri-mir-9-5, -9-4 and -9-1 (iv, grey). The study was performed at 30 hpf (A), 36-37 hpf (B) and 48 hpf (C). (D) Schematic of transverse section from zebrafish hindbrain at the level of the otic vesicle for 30-32 hpf, 36-37 hpf and 48 hpf. MZ, mantle zone; VZ, ventricular zone. Within the VZ there are dorsal progenitors (DP), medial progenitors (MP) and ventral progenitors (VP). (E) Representative example of transverse view at 36-37 hpf from triple whole-mount smiFISH labelling *neurog1* as ventral progenitor marker (VP, yellow), *ascl1* as medial progenitor marker (MP, magenta) and *atoh1* as dorsal progenitor marker (DP, cyan). The merge image shows the three progenitor markers in their respective colours, which are expressed in the VZ as described in D. (F) Representative example of transverse view at 36-37 hpf, from triple whole-mount smiFISH labelling pri-mir-9-5 (cyan) and the zebrafish Hes1 orthologues *her6* (yellow) and *her9* (magenta). The merge image shows pri-mir-9-5 (cyan) co-expressing with *her6* (yellow) and *her9* (magenta). Dashed line indicates boundary between different progenitor regions (dorsal, medial and ventral progenitor region). Scale bars: 20 µm.

Based on the smiFISH data presented above, we created a map that depicts transcription in single cells in transverse sections of the hindbrain over development (Fig. 4A-C). From left to right we observe: (i) the whole transverse section obtained from the Phalloidin staining, (ii) the region in which cells transcribe pri-mir-9-5, (iii) the cells with overlapping transcription for pri-mir-9-5/9-4 and (iv) the cells in which the three primary transcripts are transcribed (Fig. 4A-C). At 30 hpf, pri-mir-9-4 and -9-5 are co-expressed in many cells of the VZ (Fig. 4A). At 36-37 hpf, pri-mir-9-1 is transcriptionally activated in most, but not all, neural progenitors that already express pri-mir-9-4 and -9-5 (Fig. 4B). At 48 hpf the pattern of triple pri-mir-9 co-expression is similar to that seen in 36-37 hpf (Fig. 4C). This result supports the concurrent expression of pri-mir-9s at late stages but also shows their expression in the neural progenitor area, which thins during development as cells differentiate (Fig. 4D). All three paralogues are switched off in differentiating cells located in the marginal zone, suggesting that they are involved in the decision to differentiate rather than in maintaining the differentiated state (Fig. 4A-C).

To explore the identity of the triple pri-mir-9 expressing progenitors, we turned our attention to the dorso-ventral (D-V) progenitor axis of the VZ. The everted structure of the zebrafish hindbrain means that dorsal progenitors are located more laterally than medial or ventral ones (Fig. 4D). We compared the expression to *neurog1*, *ascl1* and *atoh1*, which are markers for ventral, medial and dorsal progenitors, respectively (Fig. 4E) (Tambalo et al., 2020). Remarkably, at early stages of development the cells expressing two primary transcripts were mostly localized in the ventral progenitor region of the VZ (Fig. 4A), whereas at later stages the cells with three primary transcripts excluded the ventral-most domain (Fig. 4B,C), suggesting that miR-9 high levels are required in medial and dorsal progenitors. Pri-mir-9-5 was expressed throughout the everted D-V axis (Fig. 4B,D,F) and was co-expressed with *her6* and *her9*, both of which were expressed in the progenitor domain (mainly medial and some dorsal) and are downregulated as cells differentiate. We have previously described Her6 protein expression also in ventral progenitors, which is, however, extremely weak at late development (36-37 hpf) and has not been detected by smiFISH here (Soto et al., 2020). Both *her6* and *her9* contain miR-9 binding sites and are candidates for dynamic regulation by miR-9 (Fig. 4F) (Coolen et al., 2013; Leucht et al., 2008; Soto et al., 2020).

### Knocking out the late pri-mir-1 preferentially affects neuronal differentiation from medial progenitors

**Fig. 5.**
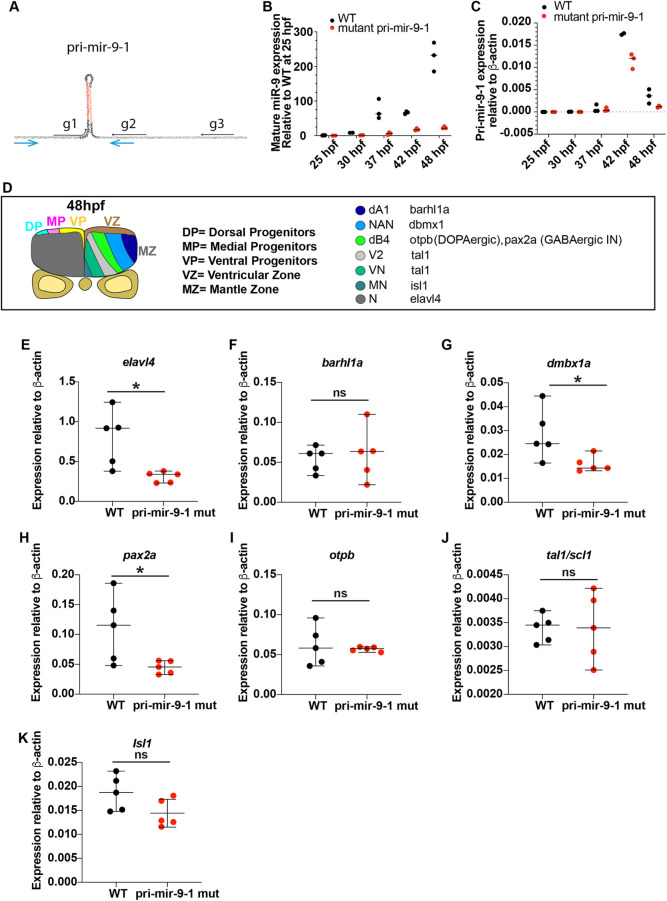
**Knocking out the late pri-mir-9-1 preferentially affects neuronal differentiation from medial progenitors.** (A) Pri-mir-9-1 hairpin loops with the respective primers used for quantitative PCR annotated as blue arrows (Materials and Methods, mRNA extraction and RT-qPCR; Table S3). Customized guide RNA to delete specifically pri-mir-9-1 are annotated as g1, g2 and g3. Red sequence, miR-9-5′ arm; orange sequence, miR-9-3′ arm; black letters, pre-mir-9; grey sequence, partial sequence of pri-mir-9-1. (B) Taqman RT-qPCR of mature miR-9 from dissected hindbrain at different stages of development, in wild-type conditions (black dots) and deletion of pri-mir-9-1 (red dots), relative to wild-type at 25 hpf. (C) SYBR green RT-qPCR relative quantification of pri-mir-9-1 from dissected hindbrain at different stages of development, in wild-type conditions (black dots) and deletion of pri-mir-9-1 (red dots). Quantification was normalised using β-actin. (D) Schematic of transverse section from zebrafish hindbrain at the level of the otic vesicle at 48 hpf. MZ, mantle zone; VZ, ventricular zone. Within the VZ there are dorsal progenitors (DP), medial progenitors (MP) and ventral progenitors (VP). The schematic shows late neuronal markers expressed in different neuronal cell types in the hindbrain: dA1, dorsal neurons expressing *barhl1a*; NAN, noradrenergic neurons expressing *dbmx1*; dB4, GABAergic interneurons expressing *pax2a*; V2, interneurons expressing *tal1*; VN, ventral neurons expressing *tal1*; MN, motor neurons expressing *isl1*; N, pan neuronal zone expressing *elavl4*; *otpb* is localised in the dB4 region but is a marker for dopaminergic neurons. (E-K) SYBR green relative quantification of *elavl4* (E), *barhl1a* (F), *dmbx1a* (G), *pax2a* (H), *otpb* (I), *tal1/scl1* (J) and *isl1* (K) from dissected hindbrain at 72 hpf, in wild-type conditions (black dots) and deletion of pri-mir-9-1 (red dots). Quantification was normalised using β-actin. Horizontal bars indicate median with 95% confidence intervals. *P<0.05 (Mann-Whitney two-tailed test). (B-C) *N*=3, each N contain a pool of 10 hindbrain. (E-K) *N*=5. ns, not significant.

The spatial analysis above showed that the expression of pri-mir-9-1 is added onto to pre-existing pri-mir-9-4 and -9-5 expression in medial and dorsal progenitors. To find out whether there is any specificity in deleting pri-mir-9-1, we designed a CRISPR/Cas9-based knockdown with guides that were specific to pri-mir-9-1 (Fig. 5A; Fig. S4A-C). This resulted in reduction of mature miR-9 and pri-mir-9-1 from 37 hpf onwards when the endogenous locus was transcribed (Fig. 5B,C). RT-qPCR was also performed to pri-mir-9-3, -9-4 and -9-5 under mutation of pri-mir-9-1. Some reduction (with high variability between samples) was also observed in pri-mir-9-4 and -9-5, but it was not maintained at later stages of development (Fig. S4E,F, 48 hpf). Pri-mir-9-3 was not affected (Fig. S4D).

Exploring the potential defect further we used a panel of differentiation markers spanning the D-V axis (Fig. 5D). Injected fish did not show overt abnormalities; however, RT-qPCR analysis at 3 days post-fertilisation (dpf) showed that the differentiation marker *elavl4* was reduced (Fig. 5E). This analysis also showed that, within the *her6* domain, there was a reduction of noradrenergic neurons (NAN) derived from medial progenitors (*dmbx1a*; Fig. 5G) and adjacent GABAergic interneurons (*pax2a*; Fig. 5H), while the more ventral neuronal markers *tal1* and *isl1* (*isl1a*) were not significantly different, neither was the most dorsal marker (*barhl1a*) outside the *her6* domain (Fig. 5F,I-K).

Medial/dorsal progenitors differentiate later in vertebrate development than ventral ones (Delile et al., 2019), therefore our findings suggest that the late increase of miR-9, afforded by the additional deployment of pri-mir-9-1, is needed for cells to adopt a late neuronal fate.

### A miR-9 stepwise increase may be required to overcome adaptation of downstream target expression

**Fig. 6.**
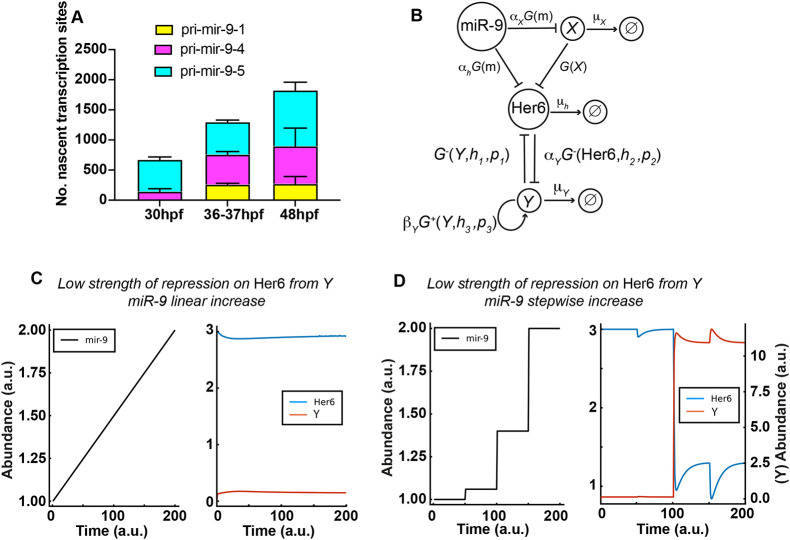
**A miR-9 stepwise increase may be required to overcome adaptation of downstream target expression.** (A) Graph representing the number of nascent transcription sites for pri-mir-9-1 (yellow), pri-mir-9-4 (magenta) and pri-mir-9-5 (cyan) at 30 hpf, 37-38 hpf and 48 hpf in 25 μm thick transverse sections. 30 hpf, *N*=4; 37-37 hpf, *N*=4; 48 hpf, *N*=3. Data are mean±s.d. (B) Schematic of the extended mathematical model, which combines an incoherent feedforward loop with an additional mutually repressive self-activating downstream target, *Y*. The parameters *α*_*h*_, *α*_*X*_ and *α*_*Y*_ represent the basal production rates of *h*, *X* and *Y*, respectively. *μ*_*h*_, *μ*_*X*_ and *μ*_*Y*_ represent the degradation rates of *h*, *X* and *Y*, respectively, and *β*_*Y*_ represents the production rate of Y under self-activation. The *h*_*i*_ and *p*_*i*_ are Hill coefficients and repression thresholds, respectively, for each of the Hill functions 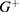 and 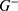, and *G(x)*=1/x. The model is described in detail in the Materials and Methods, Mathematical modelling (subsection Extended model) and specific parameter values are listed in Table S14. (C,D) Dynamics of Her6 in response to different miR-9 expression profiles, for the extended model. (C) A linear miR-9 expression profile leads to a small initial response in Her6 expression levels, which returns to steady state levels owing to the perfect adaptation. (D) Large instantaneous changes in miR-9 can result in a change in steady state for Her6. The initial step change is not sufficient to cause a change in steady state, therefore we introduce a fold change in the stepwise increase of miR-9, which activates *Y* and represses Her6 into a lower steady state.

Having shown that the increase in miR-9 in development is functionally important for differentiation, we wanted to explore whether the shape of the increase is also important. In other words, whether the way that miR-9 increases in steps can be decoded. This was motivated by the biological evidence obtained from smiFISH and RT-qPCR experiments in which we observed a stepwise sharp increase of the primary transcripts (Fig. 6A) and the mature miR-9 (Fig. 1B) over time. We used a mathematical model to ask whether a simple network of gene interactions can differentially respond to a stepwise increase of miR-9 rather than a gradual increase.

Biological systems need to be robust to stochastic fluctuations that are due to low copy numbers, or to random perturbations in the surrounding environment. This is referred to as adaptation in the context of a particular output of interest, making the biological system resistant to changes of the input. However, some changes of signals are not simply due to noise or environmental fluctuations, and adaptive systems may therefore also have to respond to specific signals under changing conditions, especially during development, in order to move into a new state. Thus, we explored whether a stepwise change in gene expression can allow a system to move out of an adaptively stable state. As incoherent feed-forward loops (IFFL) are common in biology (Goentoro et al., 2009; Shen-Orr et al., 2002) and have been shown to enable adaptation (Khammash, 2021), we hypothesized the existence of such a network centred around miR-9 as the input and Her6 as the output (Fig. S5A; Materials and Methods, Mathematical modelling) in which miR-9 affects Her6 negatively (directly) but also positively (indirectly) via repressing a repressor, *X*. Here, miR-9 directly reduces the rate of production of Her6 protein as well as the rate of production of an intermediate (unknown) species *X*. Similarly, the production of Her6 is repressed by *X* (Fig. S5A; parameter values, Table S14). Mathematically, we say that Her6 adapts perfectly to changes in miR-9 as, in this model, the steady state of Her6 is independent of miR-9 (Materials and Methods, Mathematical modelling, Steady state calculation of Her6 in the perfect adaptation model). The speed of this adaptation is controlled by the difference in reaction speed of the direct and indirect interactions between miR-9 and Her6. If the direct interaction is much faster than the indirect interaction, Her6 returns to steady state slowly after miR-9 copy numbers are perturbed. However, if the indirect interaction is faster, adaptation occurs quickly.

Such ‘perfect adaptation’ is beneficial because it allows stable mean expression of Her6 in the presence of fluctuations of miR-9 expression (Fig. S5B,C). Conversely, no changes in miR-9, i.e. linear or stepwise, can lead to persistent downregulation of Her6. As Her6 is downregulated in response to increasing miR-9 levels during development, there would need to be an additional mechanism that enables the controlled escape from perfect adaptation. To investigate this, we extended our model to include such a potential mechanism (Fig. 6B). Specifically, we introduced a downstream target of Her6, named *Y*, which self-activates and interacts with Her6 through mutual repression [as we have already previously hypothesised in Soto et al. (2020)]. The different behaviours of this system can be seen in Fig. 6C,D. A linear increase in miR-9 leads to an initial repression of Her6, which then proceeds to return to its unperturbed steady state, due to the perfect adaptation (Fig. 6C; Fig. S6A). However, following a sharp increase of miR-9, the concentration of Her6 decreases more strongly. This is sufficient for *Y* to overcome the repression from Her6, so that it can self-activate and in turn repress Her6 into a new, lower steady state (Fig. 6D; Fig. S6B). Hence, this extended motif can indeed overcome the built-in adaptation. Importantly, the escape from adaptation is triggered by a step-like change in miR-9 expression and cannot be achieved through gradual changes in miR-9 expression, or small-scale fluctuations.

The qualitative behaviour of the model is not sensitive to different values of the parameter *p*_1_, which regulates the strength of repression of Her6 by *Y*. The precise choice of *p*_1_ simply modulates the level of the lower state of Her6 expression (Fig. 6D versus Fig. S6B).

### Knocking out the late pri-mir-1 results in failure to downregulate Her6 in late developmental stages

**Fig. 7.**
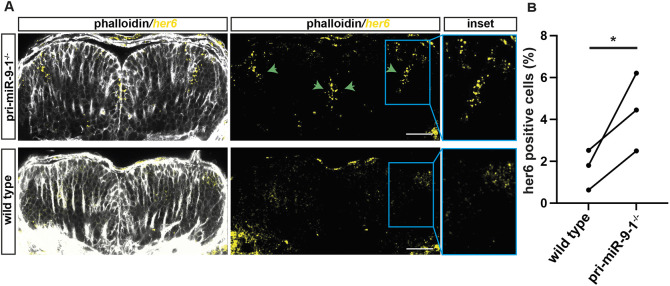
**Knocking out the late pri-mir-9-1 impairs *her6* downregulation over the course of development.** (A) Representative example of transverse view from whole-mount smiFISH labelling *her6* transcript (yellow) combined with cell boundary staining, Phalloidin-Alexa Fluor 488 (grey), in hindbrain from pri-mir-9-1 homozygote mutant (pri-mir-9-1^−/−^) (bottom panels) and wild-type (top panels) embryos at 48 hpf. Insets are increased magnification from representative images from boxed area. The images are maximum projections of three *z*-stacks, 1.89 mm. Green arrows indicate regions with high *her6* expression levels in pri-mir-9-1^−/−^ mutants. (B) Percentage of cells expressing *her6* relative to total number of cells. Pairwise comparison of *her6*-positive cells; dots indicate mean per experiment from wild-type (two embryos, three embryos, four embryos; three independent experiments) and pri-mir-9-1 homozygote mutant (two embryos, two embryos, three embryos; three independent experiments); *P=0.041 (one-tailed paired *t*-test). Scale bars: 30 µm.

The prediction from the mathematical model is that a stepwise increase of miR-9 is needed for Her6 protein to transition to a new gene expression state. We have previously shown that, during development, Her6 expression undergoes a transition from noisy to oscillatory to downregulation (Soto et al., 2020). Therefore, we performed *her6* smiFISH in transverse sections of the hindbrain to quantify the percentage of *her6*-positive cells in a wild-type and in a pri-mir-9-1 homozygous mutant (pri-mir-9-1^−/−^) stable fish line (Fig. S7A-C). In the pri-mir-9-1 mutants, at 48 hpf *her6* was not downregulated in medial progenitors (Fig. 7A,B) nor in ventral progenitors where the expression is normally lower (Soto et al., 2020). The lack of *her6* downregulation was confirmed with WM-ISH (Fig. S7D) and with live imaging of homozygous Her6::Venus knock-in zebrafish (Soto et al., 2020), which were also heterozygous for the pri-mir-9-1 mutation (Fig. S7E, pri-mir-9-1^+/−^). Therefore, our findings suggest that the late activation of pri-mir-9-1 contributes to the increase of miR-9 needed to downregulate Her6/Her9 in late neural progenitors so that they can give rise to a spatiotemporally appropriate neuronal fate.

## DISCUSSION

miR-9 is expressed from several genomic loci which, after transcription and processing, produce the same 5′ mature form of miR-9 that targets the key neural progenitor transcription factors, Her/Hes. How common is this multi-locus organisation? In humans, only 6.3% of mature miR arms are identical across two or more loci (Kozomara et al., 2019): it is thus not very common, but it is not unique to miR-9. In zebrafish this number rises to around 32.3% (Kozomara et al., 2019). The higher number of miR expressed from multiple loci is possibly due to the teleost-specific WGD. Evidence from rainbow trout also shows that, following the salmonid-specific extra round of WGD, miRs appear to be retained at higher levels than protein-coding genes (Berthelot et al., 2014). This may suggest that extra copies of miR are evolutionarily advantageous. Here, we propose that retention of multiple miR loci could have specific functional advantages for regulatory control of target gene expression of an organism. By examining in detail the temporal and spatial expression at a single cell level of three selected early and late pri-mir-9s, from across their phylogenetic tree, we offer two possible, not-mutually exclusive, explanations for this multi-site organisation or primary transcripts.

The first explanation involves a qualitative mechanism. In this scenario, distinct pri-mir-9s have a different spatial expression, which allows them to target different, i.e., region-specific, gene expression. Some differences in the spatial expression of pri-mir-9s are easily discernible at low resolution (e.g. differential expression in the forebrain), whereas others are subtle and require post-hybridisation sectioning to document, as we have done here. An example of the latter is the expression of pri-mir-9-1 which extends more dorsally in the hindbrain than pri-mir-9-4 at a late stage of development. This correlates well with the expression of Her6 and Her9, which are both miR-9 targets but are expressed adjacent to each other along the D-V axis (Soto et al., 2020).

The second explanation favours a quantitative mechanism. In this scenario, the differential temporal expression, where some primary transcripts commence their expression early while others are only expressed late, results in the simultaneous expression of both (or more) transcriptional loci in the same cells at a particular time in development. In support of this scenario, we have shown using smiFISH that pri-mir-9-1, a late onset primary transcript, is co-expressed in the same cells as the earlier onset pri-mir-9-4 or -9-5. This co-expression may be a strategy to increase the amount of miR-9 available to the cell to a level more than that possible with transcription from one locus alone.

Why would an increase in mature miR-9 over time be needed? One possibility is raised by the recent work from Amin et al. (2021), who demonstrated that miR-dependent phenotypes emerge at particular dose ranges because of hidden regulatory inflection points of their underlying gene networks. This indicates that the miR cellular dose is a major determinant of *in vivo* neuronal mRNA target selection. A complementary scenario is supported by our previous work where we have shown that the dynamical profile of Hes1 (i.e. oscillatory expression to stable expression of different levels), as well as the amount of time that Hes1 oscillates for, depends on the amount of miR-9 in the cell (Goodfellow et al., 2014; Phillips et al., 2016; Bonev et al., 2012). More recently, we have also shown, using *in vivo* manipulations, that the input of miR-9 changes the dynamic expression of Her6 from noisy to oscillatory and then to decreasing (Soto et al., 2020).

Taken together, these findings suggest that variations in the dose level of a single miR achieved by additive transcription can exert regulatory effects either by targeting different downstream gene products or by modifying the dynamic expression of the same targets. Both scenarios are compatible with experimental results, whereby mutating the late-onset pri-mir-9-1 preferentially reduced the appearance of markers for neurons that differentiate late. This suggests that the late miR-9 increase is important for late cell fate choices. This is further supported by our previous work reporting a complete repression of neurogenesis along the D-V axis of the hindbrain (Bonev et al., 2011) when total miR-9 is knocked out, whereas in this article pri-mir-9-1 knockout (KO) has more specific effect.

In other cases where multiple paralogues of an miR have been described, differential and non-mutually exclusive qualitative and quantitative regulation may also take place. For example, a recent study found that miR-196 paralogues show both unique and overlapping expression in the mouse (Wong et al., 2015). In this study, single KOs showed some unique phenotypes (qualitative mechanisms) but combinatorial KOs showed better penetrance and additional defects, suggesting an additive role of miR-196 paralogues in establishing vertebral number (quantitative mechanism).

A salient finding from our analysis is that the increase in the amount of miR-9 present in the cell is sharp, as one would perhaps expect by the onset of transcription from additional loci. An exciting possibility, supported by our mathematical modelling, is the existence of gene network motifs that do not respond to slow increases of miR-9 because they are designed to show adaptation, that is, to have steady output in spite of external perturbations. Such network motifs often involve IFFLs, which in turn are very common in biological systems because of their multiple advantages, including fold-change detection and robustness of output (Goentoro et al., 2009; Khammash, 2021). However, in development, cells also need to transition from one state to another in order to diversify cell fates, which is essential for the development of multicellular organisms. Thus, despite the usefulness of adaptation for robustness and homeostasis (Khammash, 2021), a mechanism must exist to be able to over-ride it. We suggest that, in the case of miR-9, a sharp, non-linear increase may be needed to push a dynamical system into a new state and this may be associated with a cell fate change. In our case, we suggest that the increase of miR-9 during development serves to drive the dynamics of Her6 (and other targets) from one state to another, which may include temporal downregulation, and which in turn is important for the sequential acquisition of cell fates.

At present, our computational model is qualitative, rather than quantitative, and the identity of some interacting genes in the network motif are not known. For example, we postulate the existence of a gene *X* that lies between miR-9 and Her6. Interestingly, a preliminary bioinformatic screen using transcription factor binding profiles from JASPAR (Castro-Mondragon et al., 2022) and miR target predictions for miR-9 from TargetScanFish (Release8.0) (McGeary et al., 2019) has identified Onecut, among others, as a potential candidate for factor X, which is a predicted regulator of Her6 and a direct target of miR-9. This is encouraging because Onecut is expressed in the zebrafish hindbrain, is a validated miR-9 target (Madelaine et al., 2017; Bonev et al., 2011) and a temporal factor for mammalian neurogenesis (Sagner et al., 2021).

Despite these limitations, this model was conceptually useful to illustrate the existence of a system that can decode and distinguish between specific upstream signalling profiles. Interestingly, miRs are very commonly involved in transcription factor network motifs, including IFFLs (Tsang et al., 2007). However, the regulation of each pri-mir-9 is presently unknown, but miRs are often involved in reciprocal interactions with transcription factors (Minchington et al., 2020). A fully parameterized model based on experimental evidence and identification of the unknown components/genes would be needed before it can be tested further.

In conclusion, by providing evidence for both a quantitative and qualitative mechanism, we have shed light on the possible roles of organising pri-mir-9s in several distinct genomic loci, which may have led to their evolutionary conservation. An added benefit of our work is that the detailed characterisation we have described here will enable the selection of the correct genomic locus for genetic manipulation of miR-9 production, depending on the precise spatio-temporal expression. It would be interesting to see whether the same mechanism is observed in mammalian species that have three distinct primary miR-9s.

## MATERIALS AND METHODS

### Research animals

Animal experiments were performed under UK Home Office project licences (PFDA14F2D) within the conditions of the Animal (Scientific Procedures) Act 1986. Animals were only handled by personal licence holders.

### mRNA extraction and RT-qPCR

miRs and total mRNA were extracted from a pool of ten zebrafish hindbrains using the miRVana miRNA Isolation kit and gDNA removed using DNase1 (New England Biolabs). Reverse transcription was performed with either TaqMan MicroRNA Reverse Transcription kit (Applied Biosystems) for mature miR-9 or SuperScript III (Invitrogen) with random hexamers for pri-mirs. Each qPCR reaction was prepared in triplicate in a 96-well plate with the relevant TaqMan MicroRNA assay or using POWER SYBR Green Mastermix (Thermo Fisher Scientific), 0.2 μM each forward and reverse primer (see Table S3 for respective primers) and 50 ng cDNA. Reactions were run on Step One Plus Real-time PCR System (Applied Biosystems) alongside negative controls. The data for each sample were normalized to the expression level of U6 snRNA for mature miR-9 or *b-actin* for pri-mir-9s and analysed by the 2^−ΔΔCt^ method. For each primer pair, the PCR product was examined by gel electrophoresis and its melting curve to ensure a single fragment of the predicted molecular weight.

### Molecular cloning

RNA probes for pri-mir-9-1, pri-mir-9-2, pri-mir-9-4, pri-mir-9-5 and pri-mir-9-7 were PCR amplified and cloned into pCRII vector using primers described in Table S1. Except for pri-mir-9-2 probe, they were designed to distinguish the primary transcripts by including sequences, intron and exon, before and after each miR processing, while also covering the sequence corresponding to mature miR-9 (Fig. S2). As the mature miR-9 sequence is conserved between paralogues, to avoid any cross-binding of probes to this sequence we mutated it on each probe using QuikChange II XL Site-Directed Mutagenesis assay. This allowed us introduce deletions and single nucleotide exchange in specific regions of the mature miR-9 sequence (Table S2; Fig. S2, sequence highlighted in red). pri-mir-9-3 and pri-mir-9-6 probes were generated from plasmids kindly gifted by Laure Bally-Cuif (Nepal et al., 2016).

### Whole-mount chromogenic and fluorescence *in situ* hybridization and sectioning

Chromogenic *in situ* hybridisation was performed as previously described (Thisse and Thisse, 2008) using specific probes for each pri-mir-9 (described in the Materials and Methods, Molecular cloning) and *her6* (previously used in Soto et al., 2020). The antibody used to detect the riboprobes was AP-anti-DIG (Roche, 11093274910, 1:1000). Multicolour fluorescence *in situ* hybridisation was modified from Lea et al. (2012) by developing with tyramide amplification (Perkin Elmer) after addition of antisense RNA probes and antibodies conjugated to horseradish peroxidase, anti-DIG-POD (Roche, 11207733910, 1:1000) and anti-FITC-POD (Roche, 11426346910, 1:500) (Lea et al., 2012).

Transverse sections were obtained as described in Dubaissi et al. (2012) with modifications. Embryos were embedded in 25% fish gelatine and 30% sucrose for a minimum of 24 h. We collected 18 µm thickness hindbrain sections and transferred them onto superfrost glass slides. The slides were air dried overnight under the fume hood and mounted with Prolong Diamond Antifade.

### Imaging

Chromogenic *in situs* were imaged using a Leica M165FC with a DFC7000T camera. Fluorescent *in situ* sections were imaged using Leica TCS SP5 upright confocal with HCX PL APO LU-V-I 20×0.5 water UV lens or Olympus FLUOVIEW FV1000 confocal with UPLSAPO 20× NA:0.75 lens.

### smiFISH probe design and synthesis

The smiFISH probes were designed using the probe design tool at http://www.biosearchtech.com/stellarisdesigner/. The software can assign varied size of probes, 18-22 nt, therefore we gave a size of 20 nt for all designed probes with the maximum masking level available for zebrafish. Using the respective pri-mir-9 sequence we designed 36 probes for pri-mir-9-1, 35 probes for pri-mir-9-4 and 35 probes for pri-mir-9-5 (Tables S5-S7, respectively). Using the respective gene mature mRNA sequence, we designed 29 probes for *her6*, 33 probes for *her9*, 40 probes for *neurog1*, 39 probes for *atoh1a* and 40 probes for *ascl1a* (Tables S8-S12, respectively). The designed probes were X-FLAP tagged (5′-CCTCCTAAGTTTCGAGCTGGACTCAGTG-3′) at the 5′ of each gene-specific sequence. The gene-specific probes were ordered from Integrated DNA Technologies (IDT) in a 96-well format in nuclease-free water, 100 µM concentration. Upon arrival, we combined 100 µl of the gene-specific probes together, mixed, split into 100 µl aliquots and stored at −20°C. In addition, we ordered fluo-FLAP sequences (5′-CACTGAGTCCAGCTCGAAACTTAGGAGG-3′) from either IDT or Biosearch Technology. These were labelled with either Atto-550, CalFluor-610 or AlexaFluor-647. Each gene-specific probe mix was labelled by mixing 2 µl of the gene-specific X-FLAP probe mix (100 µM), 2.5 µl of fluo-FLAP (100 µM) and 5 µl of 10× NEBuffer 3 (New England Biolabs) in a final volume of 50 µl. The hybridisation cycle was 85°C for 3 min, 65°C for 3 min and 25°C for 3 min. The labelled probe was stored at −20°C.

### Whole mount smiFISH

The whole-mount smiFISH protocol for zebrafish embryos was developed by adapting smiFISH protocol from Marra et al. (2019). Embryos were fixed in 4% formaldehyde in 1× PBS. After smiFISH protocol, embryos were stained with Phalloidin-Alexa Fluor 488 (400× dilution in PBS 1× Tween 0.1%) for 1 h at room temperature and followed by three washes with PBS-Tween. Embryos were embedded in 4% low melting point agarose (Sigma-Aldrich) to collect 250 µm thickness hindbrain transverse sections.

### smiFISH microscopy and deconvolution

smiFISH images were collected using a Leica TCS SP8 upright confocal with HC APO L U-V-I 63×/0.9 water lens, magnification 0.75×. We acquired three-dimensional stacks of 1024×1024 pixels and *z*-size 0.63 µm, magnification 0.75×, 16 bits per pixel, pinhole of 1 airy unit and scan speed of 200. Channels were sequentially imaged. smiFISH images were collected with frame accuracy 3 and line average 6.

To quantify *her6*-positive cells from smiFISH images we acquired three-dimensional stacks of 1024×1024 pixels and *z*-stacks 43-51, covering a total of 27-32 μm, that is approximately the size of half to one rhombomere (voxel size *x*:0.229, *y*:0.229, *z*:0.63 µm).

Deconvolution of confocal images was performed using Huygens Professional Software. As pre-processing steps, the images were adjusted for ‘microscopic parameters’ and ‘object stabilizer’ as additional restoration, the latter was used to adjust for any drift during imaging. Following this, we used the deconvolution Wizard tool, the two main factors to adjust during deconvolution were the background values and the signal-to-noise ratio. Background was manually measured for every image and channel, and the optimal signal-to-noise ratio identified for the images was value 3. After deconvolution the images were analysed using Imaris 9.5.

### smiFISH segmentation

Segmentation was performed using Phalloidin-AlexaFluor 488 as membrane marker. Using Imaris 9.5 software we selected the ‘Cells tool’ from which ‘Cells only’ was used as detection type and ‘Cell boundary’ was selected as cell detection type. Automated segmentation was performed, followed by manual curation to identify for cells incorrectly segmented.

To quantify pri-mir-9 nascent transcriptional sites and *her6* transcripts we used the ‘Spot tool’. The estimated spot diameter size was *xy* 1 µm and *z* 2 µm. We used the default parameters to identify the nascent transcriptional sites and further manual curation was performed to correct for minimal errors carried out by the software. Further on, spots were imported into the segmented cells to identify the cells that contained one, two or three pri-mir-9s.

Following membrane segmentation and quantification of *her6* transcripts, the percentage of *her6*-positive cells was calculated over the total number of cells segmented from the hindbrain transverse section (covering 27-32 μm).

### Expression analysis of Hes/Her genes and miR hosts

For the *in silico* analysis of the miR host gene expression we downloaded the time course RNA-seq data (TPM) from White et al. (2017). Here, we used the overlapping host genes as a proxy for the expression of the miR. miR would not show up in standard RNA-seq analysis and there is no current miR time course data. Host genes were identified as those with overlapping annotations with the miR-9 genes. The host genes for each pri-mir-9 are in Table S4. Pri-mir-9-7 has no overlapping annotation at this time and is thus not reported on in these data.

We filtered the RNA-seq data removing genes which were neither the host genes of the miR or members of the Her family. Three repeats for each stage of development are included in the data and we averaged the expression across the three repeats for each stage. The stages reported in the data are based on standard embryonic stages in zebrafish development. However, we wanted to visualize the expression in terms of hours and the stages were converted accordingly. Finally, before plotting, these data were *z*-scored to normalize the expression of each of the genes so that we could compare changes in expression over time rather than absolute levels. These data were then plotted using the heatmap.3 package in R.

### Deletion of pre-mir-9-1 using CRISPR/Cas9

#### Preparation of Cas9nls and sgRNAs

For pre-mir-9-1 deletion using CRISPR/Cas9, sgRNA target sites were identified using the CRISPRdirect (http://crispr.dbcls.jp/) and Target Finder (Feng Zhang lab; http://crispr.mit.edu/). sgRNAs were generated following CRISPRscan protocol (Moreno-Mateos et al., 2015) using the oligonucleotides described in Table S13. Transcription of sgRNA was carried out using MEGAshortscript T7 kit (Ambion/Invitrogen) with 100-400 ng of purified DNA following the manufacturer's instructions. After transcription sgRNA was purified using MEGAclear™ Transcription Clean-Up Kit. The Cas9nls protein was obtained from New England Biolabs (M0646T).

#### Microinjection and genotyping

One-cell stage wild-type embryos were injected with ∼1 nl of a solution containing 185 ng/μl Cas9nls protein, 125 ng/μl sgRNA, 40 ng/μl caax-mRFP mRNA in 0.05% Phenol Red. To evaluate if each sgRNA was generating mutation, genomic DNA was extracted from 3-4 dpf embryos using 50 μl NP lysis buffer per embryo [10 mM Tris (pH 8), 1 mM EDTA, 80 mM KCl, 0.3% NP40 and 0.3% Tween] and 0.5 μg/μl Proteinase K (Roche) for 3-4 h at 55°C, 15 min at 95°C and then stored at 4°C. Then, High Resolution Melt (HRM) was performed (Fig. S4A) using the Melt Doc kit (Applied Biosystems) following the manufacturer's instructions. Specific primers were designed to generate an amplicon of 395 bp in wild-type conditions: forward primer 5′-ACAGTTGACTTTCTAATTACAACCC-3′ and reverse primer 5′-AGCAGGAGGAGATAATCACAGC-3′.

To analyse the effect of pre-mir-9 deletion in F0 embryos we combined three different sgRNA flanking the region of the mature miR-9 region and they were microinjected as described above. We chose to use three sgRNAs to increase our probability of deleting the mature miR-9 sequence. The embryos were injected with 125 ng/µl of each sgRNA: this low concentration of sgRNA was used to not have overt phenotype at the macroscopic level during the experimental period (24hpf-72 hpf), minimizing the chances of non-specific toxicity. Further on, the amplicons with deletion were identified by agarose gel and sequencing (Fig. S4B,C), as described below.

To identify F1 progeny with germ line transmission (GLT), 3-5 dpf embryos were fin clipped following the protocol described by Robert Wilkinson (Wilkinson et al., 2013) with modifications. Sylgard (Sigma-Aldrich, 761028)-coated 10 cm dishes were prepared for dissections. Embryos were placed into Sylgard-coated dishes containing L15 medium with 0.1% Tricaine (Sigma-Aldrich, UK) and 5% fetal bovine serum (Sigma-Aldrich). Once fin clipped, the embryo was rinsed in E3 medium and transferred to a fresh well; the biopsy was transferred to a PCR tube for genomic extraction. Genomic extraction was carried out in 10 μl volume using Phire Animal Tissue Direct PCR kit (Thermo Fisher Scientific, F-140WH). PCR reaction was carried out with 1 μl of the genomic extraction and primers used for HRM. An amplicon of 395 bp indicates a wild-type band and 275 bp indicates a pri-mir-9-1 mutant band. To evaluate the region deleted in the F1 pri-mir-9-1 mutants, PCR was performed per embryo, the amplicon obtained was cloned into pCRII and transformed into bacteria Top10. Three bacterial colonies were miniprepped and sequenced.

### Live imaging of whole developing hindbrain

The F1 adult animals were kept as Her6::Venus^+/−^;pri-mir-9-1^+/−^ and were inbred to obtain and compare offspring such as Her6::Venus^+/+^;pri-mir-9-1^+/−^ or ^−/−^ with Her6::Venus^+/+^;pri-mir-9-1^+/+^. To perform a comparative analysis of overall Her6 expression during hindbrain development on the mixed genotype population, a pool of ten embryos were laterally mounted in 1% low-melting agarose on glass-bottom dishes (MatTek Corporation P50G-1.5-14-F) and imaged using a Zeiss LSM 880 fast Airyscan microscope, followed by genotyping. Only pairs (wild type and mutant) that were found within the same pool were analysed, to allow comparison between similar developmental stages. Parameters used were ×1 zoom; image size *x*: 425 m, *y*: 425 mm, *z*: 150 mm. Images were subject to 2D maximum projection in FIJI.

### Mathematical modelling

#### Steady state calculation of Her6 in the perfect adaptation model

The perfect adaptation model can be described by a set of differential equations (Fig. S5A; Table S14):
(1)

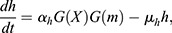

(2)

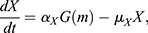
where *h* is Her6, *m* is miR-9 and *α*_*h*_, *μ*_*h*_, *α*_*X*_ and *μ*_*X*_ are positive real constants which represent the production and degradation rates of Her6 and *X*, respectively. The negative interaction between each of these model components is given by an arbitrary function G. To identify a possible shape of G, we consider the steady state of Eqns (1) and (2), which leads to:

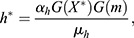


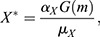
which combine to give:

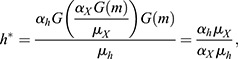
if *G* is defined by the negative interaction, *G*(*m*)=1/*m*. Hence, in our chosen model the steady state of Her6, h*, is independent of miR-9. To achieve this, we made the assumption that G is a nonlinear negative interaction, which agrees with previous models of miR-9 interactions (Goodfellow et al., 2014). In order to explore the adaptation properties of this network, we made certain simplifications over previous models (Goodfellow et al., 2014) such as omitting the *her6* autorepression, transcriptional delays and noise. Thus, this simplified Her6 network does not reproduce the oscillatory expression of Her6 but instead explores the transition between different stable steady states.

#### Extended model

The extended system can be described by the following set of differential equations (Fig. 6A; Table S14):
(3)

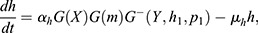

(4)

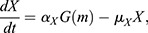

(5)

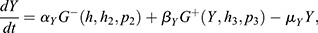


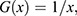


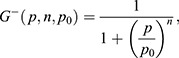


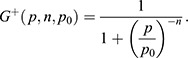


We pre-define the profile of miR-9 expression over time to interrogate both stepwise and linear expression, and then solve the system for *h*, *X* and *Y*. The parameters *α*_*h*_, *α*_*X*_ and *α*_*Y*_ represent the basal production rates of *h*, *X* and *Y*, respectively. Similarly, *μ*_*h*_, *μ*_*X*_ and *μ*_*Y*_ represent the degradation rates of *h*, *X* and *Y*, respectively, and *β*_*Y*_ represents the production rate of Y under self-activation. The *h*_*i*_ and *p*_*i*_ are Hill coefficients and repression thresholds, respectively, for each of the Hill functions. For the activating Hill function 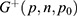 with arbitrary input parameter *p*, Hill coefficient *n* and repression threshold *p*_0_, as *p* grows much larger than *p*_0_, 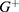 tends to 1, and as *p* goes to 0, 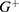 tends to 0. For 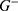 the limits are reversed, i.e. 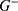 is equal to 1 for small values of *p* and goes to 0 for *p*≫*p*_0_. The Hill coefficient *n* determines the sensitivity of the function to changes in *p*, i.e. larger *n* corresponds to higher sensitivity. All parameters introduced here are constants, and their values are listed in Table S14. These parameters are chosen such that *Y* is repressed and has no effect on the system when Her6 is at its high steady state h*.

### Statistical testing

Statistical tests were performed in GraphPad Prism 9. Data were tested for normality with D'Agostino–Pearson test. Discrete scatter plots show median with 95% confidence interval where multiple independent experiments are analysed. Statistical significance between two datasets was tested with Mann–Whitney test (non-parametric). For paired experiments the data was tested for normality with Kolmogorov–Smirnov test followed by a one-tailed paired *t*-test. Sample sizes, experiment numbers and *P*-values <0.05 are reported in each figure legend.

## Supplementary Material

10.1242/develop.200474_sup1 Supplementary information
