# Supplementary material for: Sequential and additive expression of miR-9 precursors control timing of neurogenesis
Source: Development. 2022 Oct 3;149(19):dev200474. doi: 10.1242/dev.200474 (PMC9641661; doi:10.1242/dev.200474)
Supplement: Supplementary information [file develop-149-200474-s1.pdf]

Figure S1

A

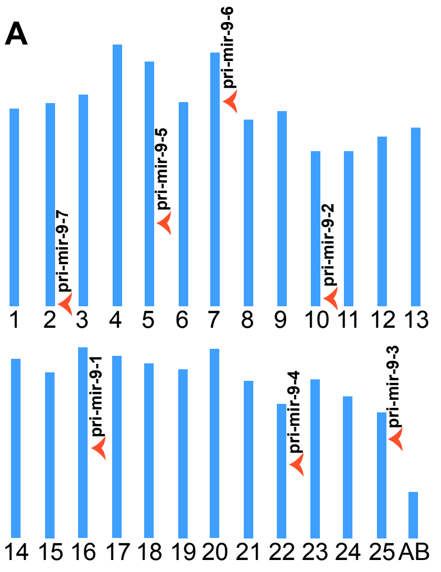

B

name	chromosome	Genomic location	strand
Pri-mir-9-1	chr16	lincRNA, intragenic/exonic	+
Pri-mir-9-2	chr10	lincRNA, intragenic/ intronic	-
Pri-mir-9-3	chr25	intergenic	-
Pri-mir-9-4	chr22	BX664610.1 protein, intragenic/intronic	+
Pri-mir-9-5	chr5	lincRNA, intragenic/exonic	-
Pri-mir-9-6	chr7	lincRNA, intragenic/exonic	-
Pri-mir-9-7	chr2	lincRNA. intragenic/intronic	-

C

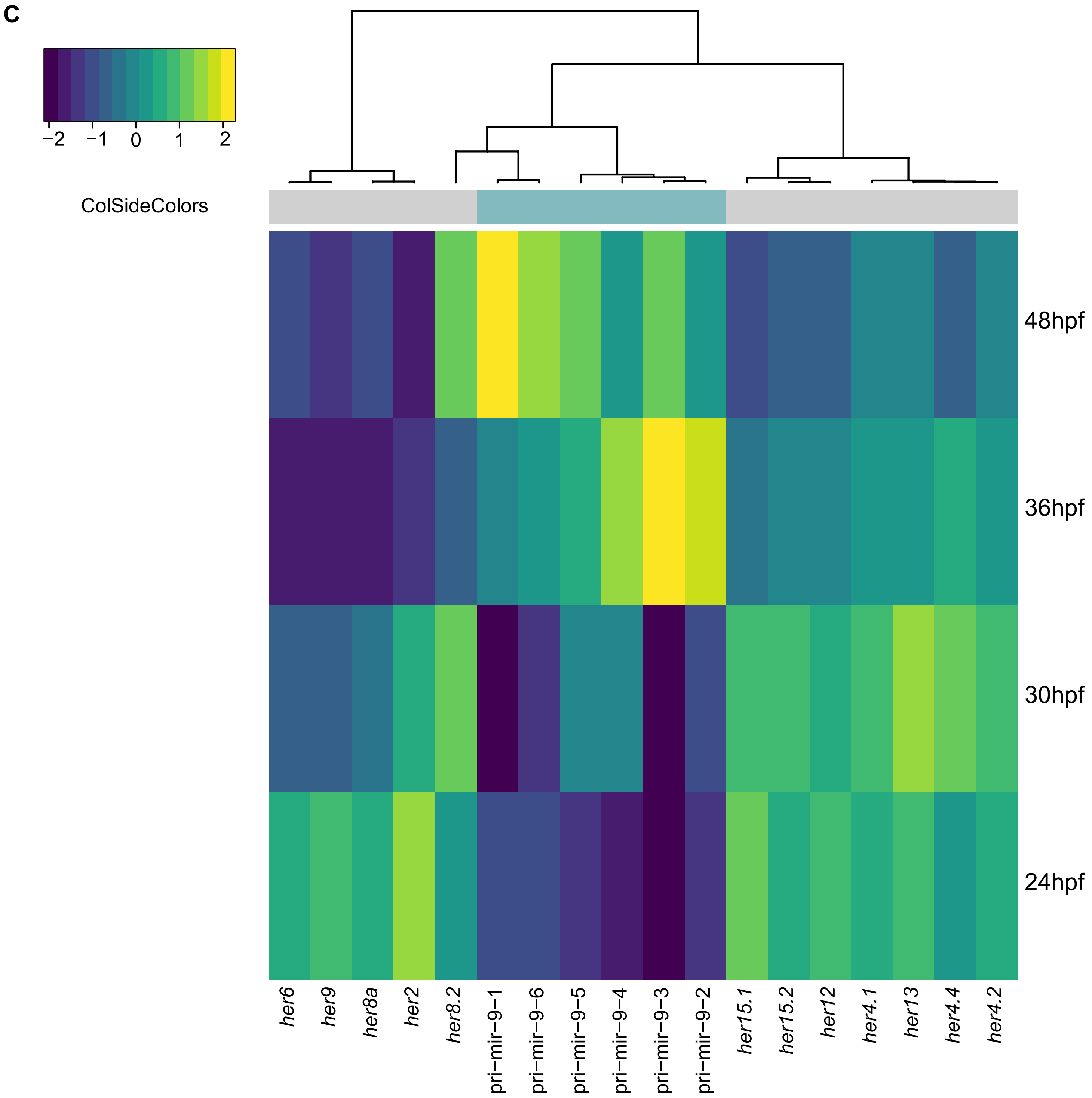

Development • Supplementary information

Fig. S1. miR-9 host gene expression negatively correlates with Hes/Her gene expression over developmental time. (A) Map of miR-9 paralogues in *Danio rerio* produced with information from Vega genome browser, data was collated from ensembl genome browser GRCz10, showing chromosomal loci. Red arrowhead indicates location of respective pri-mir-9. **(B)** Table showing the genomic loci of the seven *Danio rerio* miR-9 paralogues, their host gene, exonic or intronic location within the host gene and strand orientation**. (C)** Heatmap showing z-scored expression data over developmental time for the miR-9 host / pri-mirs and Hes/Her genes over developmental time in the zebrafish embryo. Data is from White *et al.*, 2017.

Development • Supplementary information

Figure S2

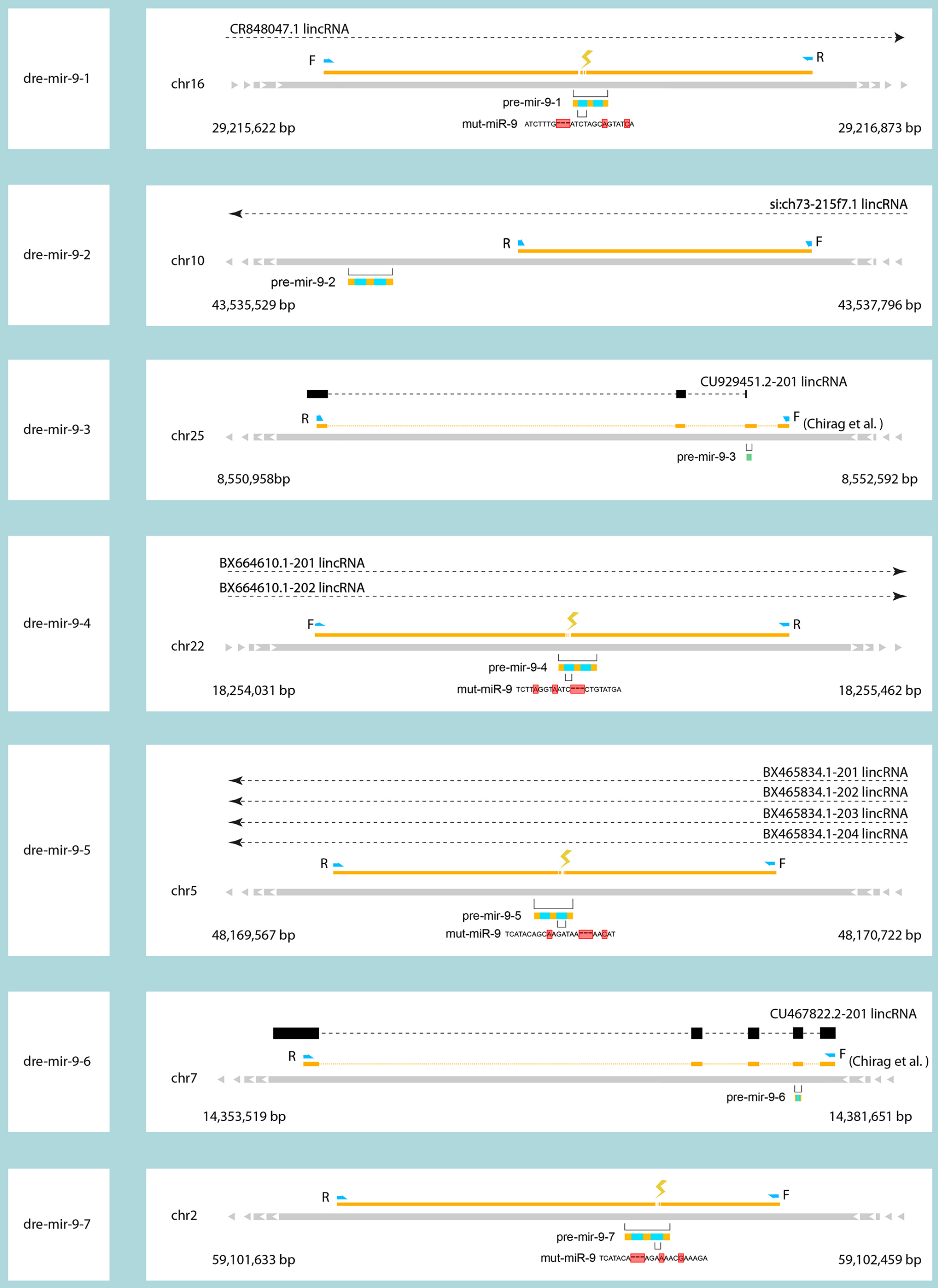

Fig. S2. Probe design for *in situ* hybridisations of pri-mir-9s. The probes are indicated by the orange blocks. Where probes are on exons, introns are indicated by dashed lines. Forward (F) and reverse (R) are visualised by blue arrows. Overlapping transcripts are indicated by the black boxes and dashed lines. The microRNA hairpin is indicated by a small orange box broken by two blue boxes indicating the mature sequences. Probes for pri-mir-9-3 and pri-mir-9-6 were based on those from Nepal et al. (2016). Mutations in the mature sequence are indicated by lightning bolts and also annotated underneath.

Development • Supplementary information

Figure S3

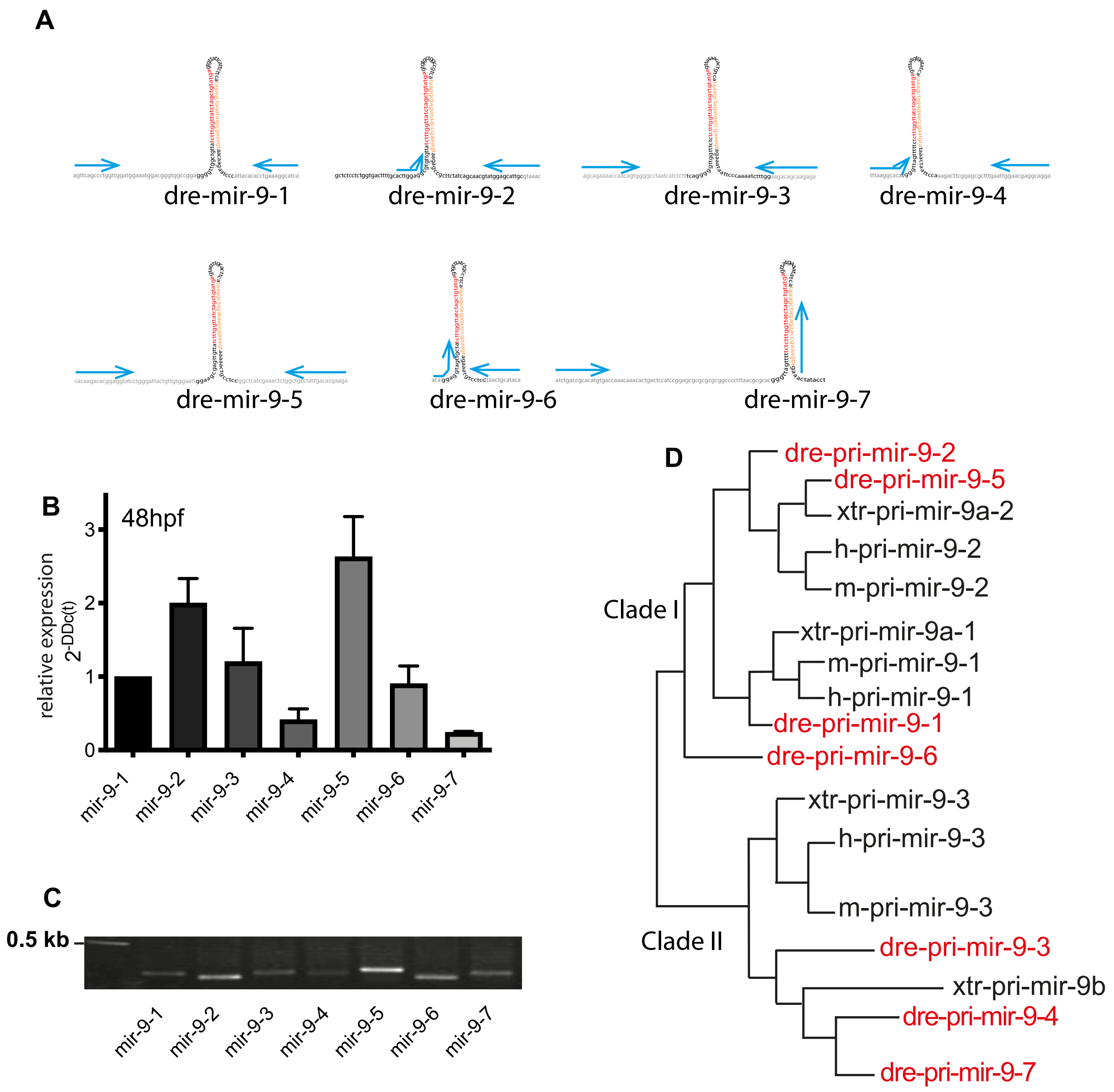

Fig. S3. All seven miR-9 paralogues are expressed at 48hpf. (A) Schematic of the seven miR-9 paralogues hairpin loops with the respective primers used for qRTPCR annotated as blue arrows **(Methods. mRNA extraction and Quantitative realtime PCR; Table S3).** Red sequence: miR-9-5'arm, orange sequence: miR-9-3' arm, black letters: pre-mir-9, grey sequence: partial sequence of pri-mir-9. (B) Bar plot showing relative expression of the seven miR-9 paralogues at 48hpf, N=3. Error bar represent mean and SEM. (C) Amplicons of the seven miR-9 paralogues generated by qRT-PCR using the respective primers **(Table S3)**. (D) Evolutionary relationship of pri-mir-9 family modified from Alwin Prem Anand et al., 2018 phylogenetic analysis. Highlighted in red letter are the seven miR-9 paralogues. The zebrafish miR-9 paralogues cluster into two Clades: Clade I that is divided into 2 subgroups: Subgroup I corresponding to the group in which human (h) and mouse (m) pri-mir-9-2 precursor cluster with *Danio rerio* (dre) pri-mir-9-2 and pri-mir-9-5 precursors and subgroup II corresponding to the group where h/m-pri-mir-9-1 precursor clusters with dre-pri-mir-9-1. Interestingly, dre-pri-mir-9-6 is closely related to clade I but doesn't group with any subgroup. Clade II correspond to the branch where m/h-pri-miR-9-3 group with dre-pri-mir-9-3, dre-pri-mir-9-4 and dre-pri-mir-9-7.

Development • Supplementary information

Figure S4

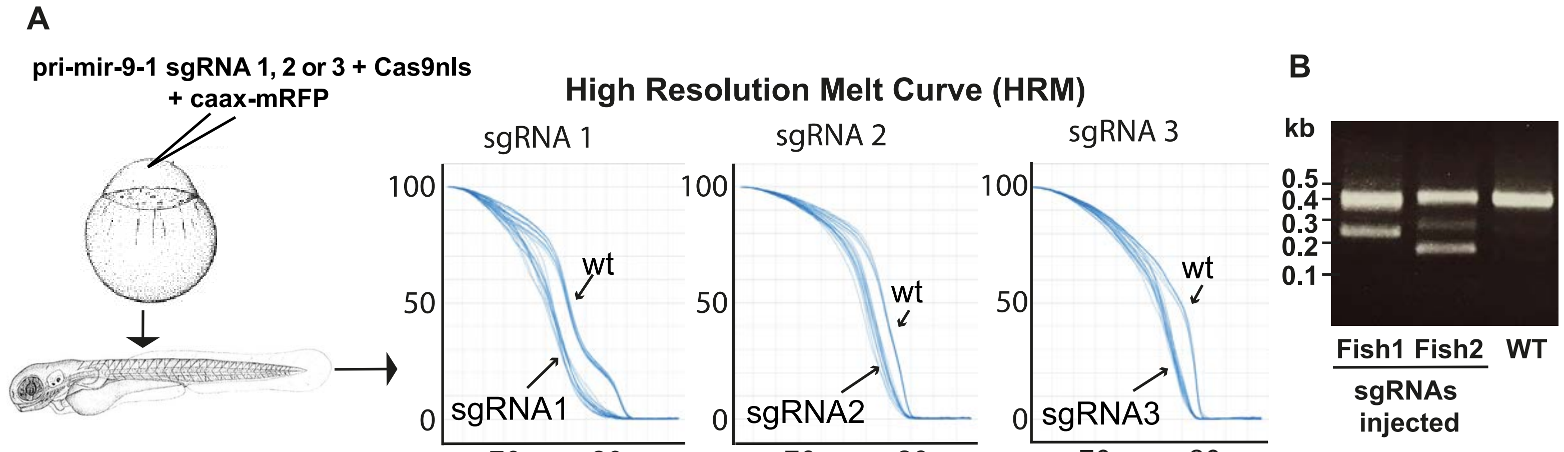

C

```
Fish1   AGTTCAGCCCTGGTTGGATGGAAATGGATGGGTGGCCGGAGG--------
Fish2   AGTTCAGCCCTGGTTGGATGGAAATGGACGGGTGGCCGGA----------
wt      AGTTCAGCCCTGGTTGGATGGAAATGGACGGGTGGCCGGAGGGGTTGGCT
        **************************** ***********

Fish1   --------------------------------------------------
Fish2   --------------------------------------------------
wt      GTTATCTTTGGTTATCTAGCTGTATGAGTGTTATTCATTCTTCATAAAGC

Fish1   -----------------------------------------AGGCATCAC
Fish2   --------------------------------------------------
wt      TAGATAACCGAAAGTAACAAGAATCCCATTACACACCTGAAAGGCATCAC

Fish1   CCCACCTTGAGATCTGTACTCCTCCCCTCTCCTCTTCTCCTCTTTTCCCT
Fish2   --------------------------------------------------
wt      CCCACCTTGAGATCTGTACTCCTCCCCTCTCCTCTTCTCCTCTTTTCCCT

Fish1   C-CAAGTCTCCCTCTCTCTCAGTTTCAATCTCTGCATCATTTTATTGTGA
Fish2   ---------------------------------------TTTTATTGTGA
wt      CCCAAGTCTCCCTCTCTCTCAGTTTCAATCTCTGCATCATTTTATTGTGA
                                               ***********

Fish1   GAGCAAGCCACAAAATGGCCTCCATCAGTGCTGATATGGAAGGTAAGCAG
Fish2   GAGCAAGCCACAAAATGGCCTCCATCAGTGCTGATATGGAAGGTAAGCAG
wt      GAGCAAGCCACAAAATGGCCTCCATCAGTGCTGATATGGAAGGTAAGCAG
        **************************************************

Fish1   CATTT---TT
Fish2   CATTTTTTAG
wt      CATTTTTTAG
        *****
```

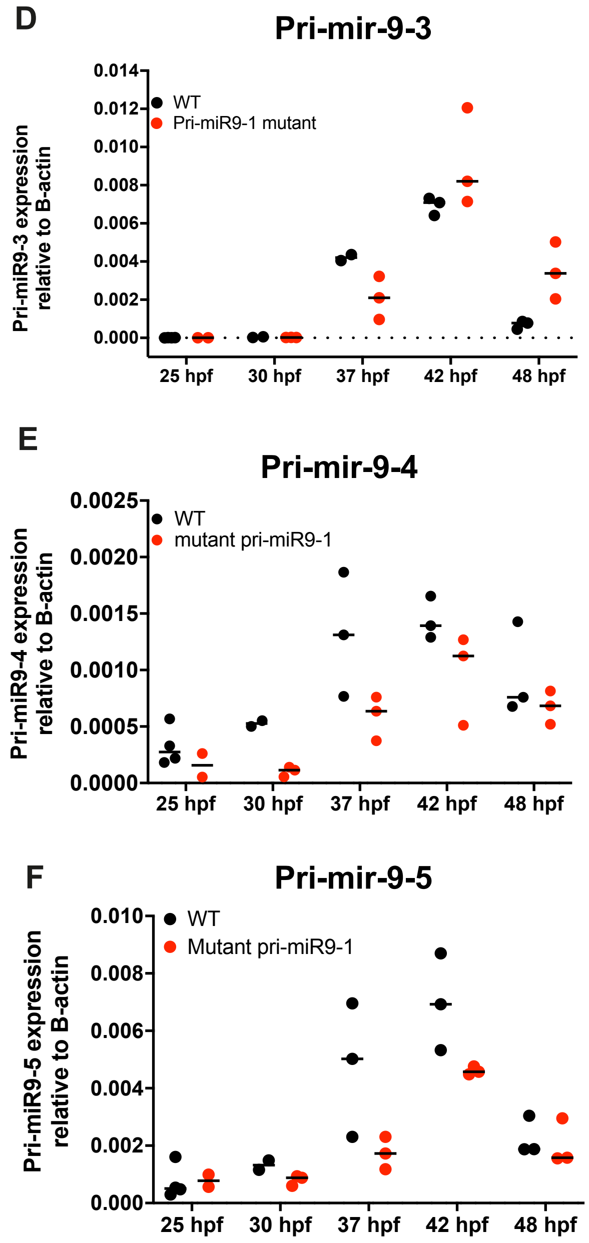

Fig. S4. Deletion of pre-mir-9-1

(A). (A-left) Schematic representation of experimental procedure used to delete premir-9-1, **(A-right)** high resolution melt graphs obtained from: wt (wildtype) versus sgRNA 1, 2 or 3 injected fish, respectively. **(B)** Agarose gel showing the size of the amplicon in wt fish (395bp) and in injected with three sgRNA fish, ~250bp and 395bp in fish1 and ~190bp and 395bp in fish2. **(C)** Representative examples of sequences obtained from F0 embryos showing deletion of pre-mir-9-1. Red underline indicates pre-mir-9-1 sequence. Green highlighted indicates mature miR-9 sequence. **(D-F)** SYBR green relative quantification of pri-mir-9-3 **(D)**, pri-mir-9-4 (E) and pri-mir-9-5 **(F)** from dissected hindbrain at different stages of development, in wild-type conditions (black dots) and deletion of pre-mir-9-1 (red dots), quantification was normalised using β-actin. N=3.

Development • Supplementary information

Figure S5

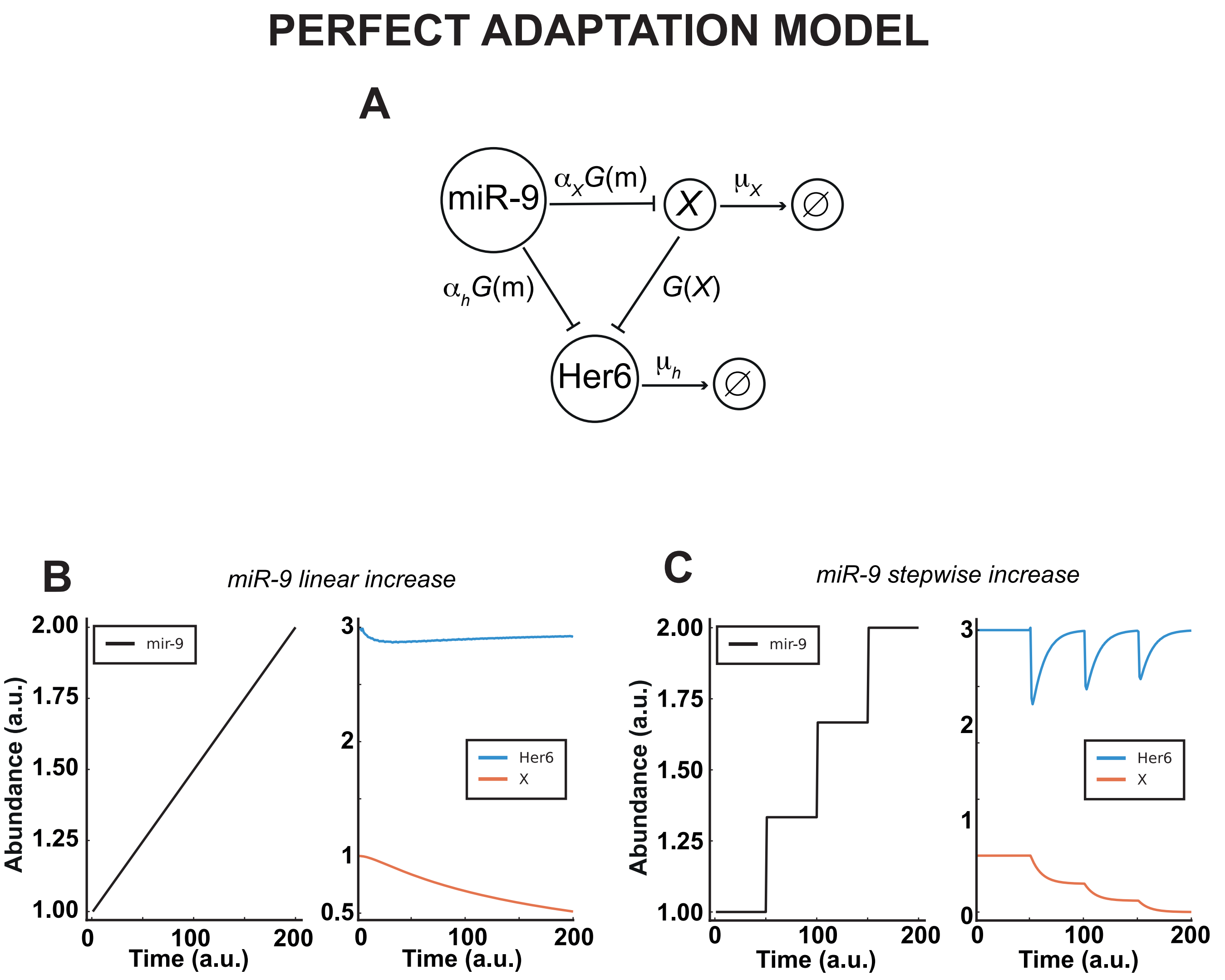

Fig. S5. Her6 is not down-regulated by miR-9 in the perfect adaptation model (A-C) Perfect adaptation model. (A) A schematic of the perfect adaptation model based on an incoherent feed forward loop. The parameters μ_h, α_h and α_X are positive real constants. μ_h represents the degradation rates of Her6 and α_h and α_X represent the basal production rate of Her6 and X respectively. See **Methods,** Mathematical modelling (a), parameter values are given in Table S14. (B) A linear mir-9 expression profile leads to a small initial response in Her6 expression levels, which returns to steady state levels due to the perfect adaptation. (C) Large instantaneous changes in miR-9 cause a large drop in Her6 levels, which then returns to steady state. The model detects fold changes in miR-9 and repetitive steps result in a diminished response from Her6.

Development • Supplementary information

Figure S6

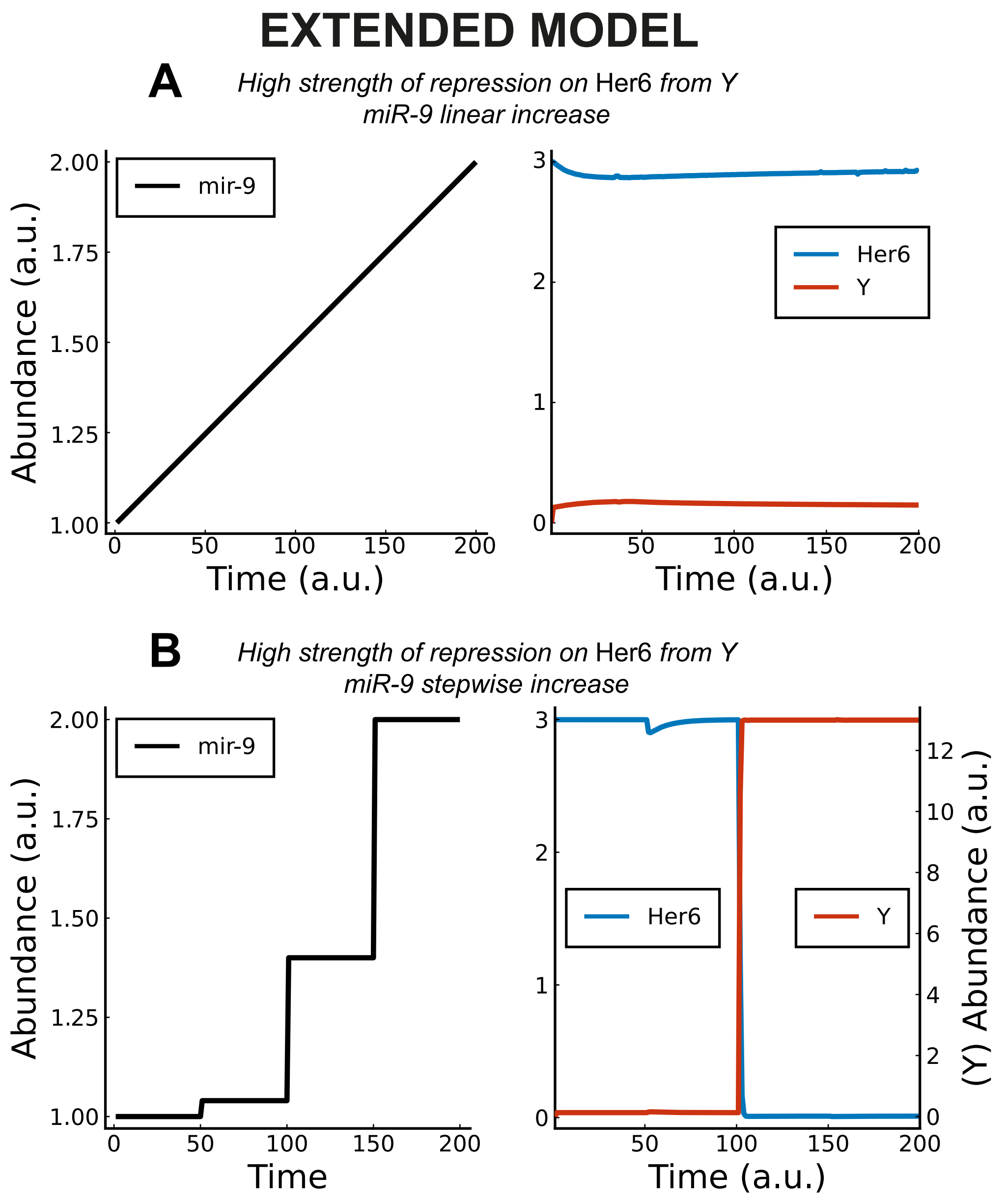

Fig. S6. Her6 is down-regulated by miR-9 stepwise increase in extended adaptation mathematical model

(A-B) Dynamics of Her6 in response to linear increase in miR-9 **(A)** or stepwise increase in miR-9 **(B)** with increased repression strength from *Y*. The stepwise increase in miR-9 under high repression strength from *Y* causes Her6 to switch off given a large enough increase in miR-9.

Development • Supplementary information

Figure S7

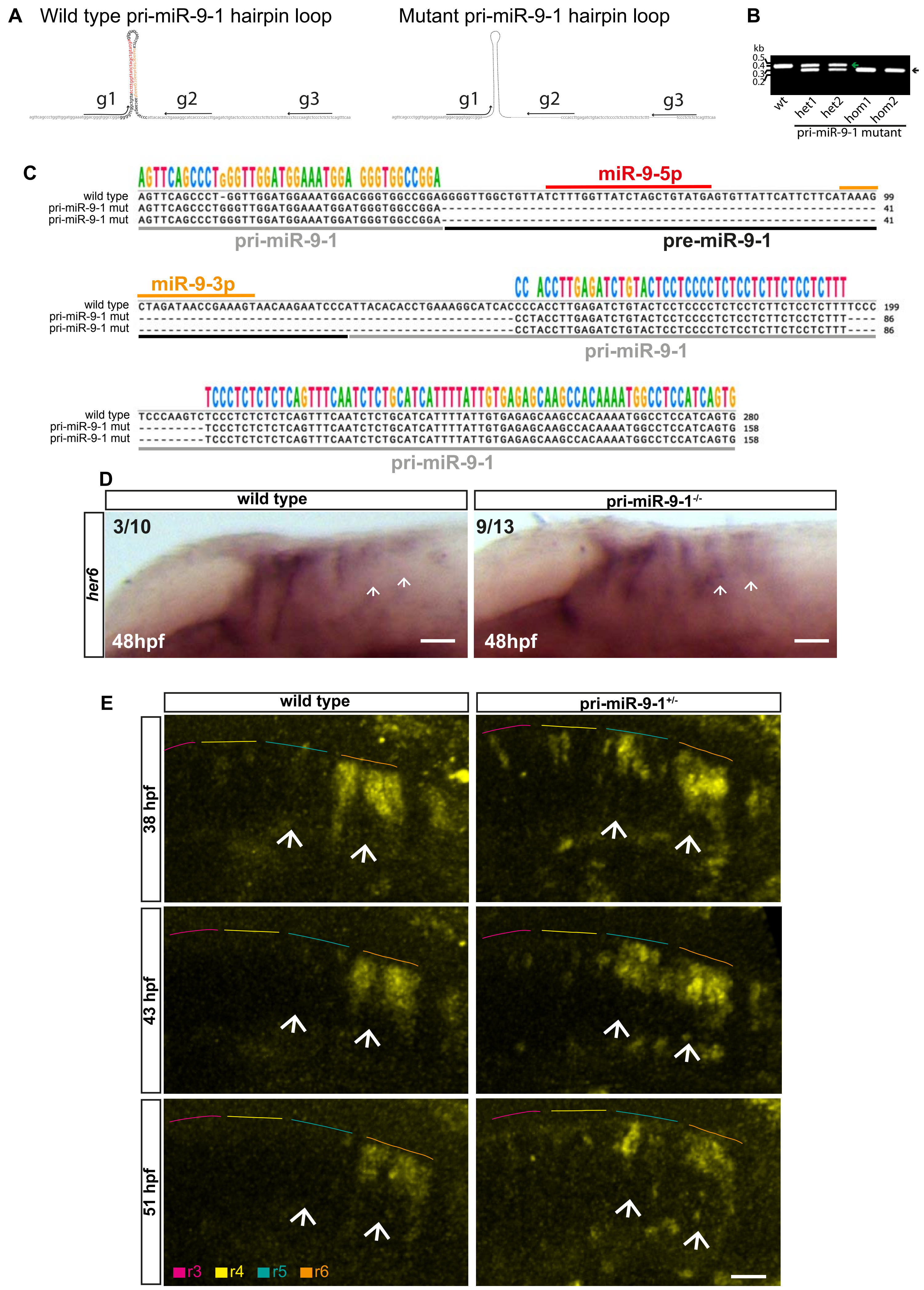

Fig. S7. Generation of pri-miR-9-1 mutant fish line. (A) Schematic of pri-miR-9-1 hairpin loops with the customized guide RNAs used to specifically delete pre-mir-9-1, annotated as g1, g2 and g3. **Left panel**, sequence of wild type pri-miR-9-1 hairpin loop. **Right panel,** sequence of mutant pri-miR-9-1 after CRISPR deletion with dashed

Development • Supplementary information

line indicating the deleted region and loss of hairpin loop. **(B)** Amplicons obtained from F1 generation of wildtype (green arrow) and pri-miR-9-1 mutant (black arrow) embryos, 395bp and 273bp, respectively. wt: wildtype; het: heterozygote; hom: homozygote. **(C)** Sequence showing the region deleted in the pri-miR-9-1 mutant. **(A and C)** Red: miR-9 5' arm, orange: miR-9 3' arm, black: pre-mir-9, grey: partial sequence of pri-mir-9-1. **(D)** Representative image of Chromogenic WM-ISH performed at 48hpf showing *her6* expression in wild type embryos and in pri-miR-9-1$^{-/-}$ mutant embryos. Note ventral extension of her6 expression in pri-miR-9-1$^{-/-}$ embryos (9/13) compared to wildtype embryos (3/10) as indicated by white arrowheads; longitudinal view, scale bar 30μm. **(E)** Representative confocal images of wild type and pri-miR-9-1$^{+/-}$ embryos showing Her6::Venus$^{+/+}$ expression in the hindbrain, rhombomeres 3 to 6 (r3-r6) over the course of development; longitudinal view, scale bar 30μm. Images are representing 2D maximum projection; N=3 independent experiments with 1 embryo, 1 embryo, 2 embryos per experimental condition, Her6::Venus$^{+/+}$;pri-mir-9-1$^{+/+}$ or Her6::Venus$^{+/+}$;pri-mir-9-1$^{-/+ or -/-}$. Note: The in-cross of Her6::Venus$^{+/-}$;pri-mir-9-1$^{+/-}$ adults had low rate of Her6::Venus$^{+/+}$;pri-mir-9-1$^{-/+ or -/-}$ embryos explaining the low number of embryos analysed per independent experiment. **(D-E)** White arrowheads indicate increased Her6::Venus expression in pri-miR-9-1 mutants.

Development • Supplementary information

Table S1. Sequences of primers used to generate probes for *in situ* hybridisation. * Primers for *Pri-mir-9-3* and *Pri-mir-9-6* were used to clone the probes that were kindly provided by the Bally-Cuif lab.

Gene	Oligonucleotides sequence 5>3
Pri-mir-9-1 f	cagattgacagagttgtgag
Pri-mir-9-1 r	cagtgctgactactctaatg
Pri-mir-9-2 f	gttcaatcctcttccgtttg
*Pri-mir-9-2 r	cagcatcccgttacacattc
*Pri-mir-9-3 f	aatgctgagttttgccacct
Pri-mir-9-3 r	tgctgcggaaaataacacaa
Pri-mir-9-4 f	ttccacaagggtatcgatag
Pri-mir-9-4 r	tattatatgagaaccacgtg
Pri-mir-9-5 f	gatgttatttccgcgtgcac
Pri-mir-9-5 r	tcagctctctctatatgtcc
*Pri-mir-9-6 f	gagagacaacatcgcatcca
*Pri-mir-9-6 r	tcaaacatagcaggatagggtct
Pri-mir-9-7 f	tactaattaacctataacgcttgc
Pri-mir-9-7 r	tctactttcggttctctagc

Table S2. Primers for site-directed mutagenesis

Number	Gene mane	Primer sequence 5>3
1	Pri-mir-9-1	aggggttggctgttatctttgatctagcagtatcagtgttattc
2	Pri-mir-9-4	tgggttagtttttctcttaggtaatcctgtatgagtttatgtgatatcataaa
3	Pri-mir-9-5	aaatactcatacagcaagataaaacataacaactcgcttccaattcc
4	Pri-mir-9-7	acgggttagtttttctctttcgttttcttgtatgagttatgaaatatcataaag

Development • Supplementary information

Table S3. Sequences of primers used for qRT-PCR

Number	name	Sequence 5'>3'	Amplicon size (bp)
1	pri-mir-9-1 f	agttcagccctggttgg	149
2	pri-mir-9-1 r	tgatgcctttcaggtgtg	
3	pri-mir-9-2 f	acttggaggcgtgttg	120
4	pri-mir-9-2 r	gtttacgcaatgctccatac	
5	pri-mir-9-3 f	agcagaaaaccaacagtgg	150
6	pri-mir-9-3 r	tctcttgctgtcttccaaag	
7	pri-mir-9-4 f	tttaaggcacatgggttag	135
8	pri-mir-9-4 r	tcctgcctcgttccaattc	
9	pri-mir-9-5 f	cacaagacacggaggtatcc	166
10	pri-mir-9-5 r	tcttcgtgtcaataggcagcc	
11	pri-mir-9-6 f	acaggaggtagttgctatc	104
12	pri-mir-9-6 r	tgtatgcagttaaggaggac	
13	pri-mir-9-7 f	atctgaccgcacatgtgac	146
14	pri-mir-9-7 r	tctactttcggttctctagc	
15	*β-actin* f	cgtgctgtcttcccatcca	176
16	*β-actin* r	tcaccaacgtagctgtctttctg	
17	*elavl4* f	ccgtcaacaatgtcaaggtg	147
18	*elavl4* r	acacttgcaagacacggtca	
19	*barhl2* f	ttcggtcacgatggagcatc	120
20	*barhl2* r	agcagatgaggctgaggtga	
21	*dmbx1a* f	gcgaccccatcatctataccat	105
22	*dmbx1a* r	cgcgtggtgagatagagactga	
23	*pax2a* f	gtgacaggtcgagagatggc	198
24	*pax2a* r	acttaataacgcggggttgct	
25	*otpb* f	tgttggagcgatttttcagg	107
26	*otpb* r	ttcctgttgatcccgtttgaa	
27	*tal1/scl* f	gcacgacggcggagaca	226
28	*tal1/scl* r	ggaggcagtggcaagggaa	
29	*isl1* f	gcagcagcaacccaacgacaa	224
30	*isl1* r	ttgagcctggaccaccttcagaa	

Table S4. microRNA host genes

**MicroRNA**	**Ensembl gene code**	**Host name**
**dre-mir-9-1**	ENSDARG00000095906	CR848047.1
**dre-mir-9-2**	ENSDARG00000098512	si:ch73-215f7.1
**dre-mir-9-3**	ENSDARG00000098395	CU929451.2
**dre-mir-9-4**	ENSDARG00000095557	BX664610.1
**dre-mir-9-5**	ENSDARG00000095715	BX465834.1
**dre-mir-9-6**	ENSDARG00000101584	CU467822.2
**dre-mir-9-7**	NA	NA

Table S5. smiFISH Probe sequences for pri-mir-9-1

Probe sequence name	Probe sequence (5' to 3')
pri-mir-9-1 smiFISH_1	CCTCCTAAGTTTCGAGCTGGACTCAGTGcactcactttctttctcaca
pri-mir-9-1 smiFISH_2	CCTCCTAAGTTTCGAGCTGGACTCAGTGgaggtctttctctctctcag
pri-mir-9-1 smiFISH_3	CCTCCTAAGTTTCGAGCTGGACTCAGTGcacagcaaagtgctagaatc
pri-mir-9-1 smiFISH_4	CCTCCTAAGTTTCGAGCTGGACTCAGTGgatttaccctcaagttgttg
pri-mir-9-1 smiFISH_5	CCTCCTAAGTTTCGAGCTGGACTCAGTGctagttgctttagttttccg
pri-mir-9-1 smiFISH_6	CCTCCTAAGTTTCGAGCTGGACTCAGTGaataggggggaaaagaccct
pri-mir-9-1 smiFISH_7	CCTCCTAAGTTTCGAGCTGGACTCAGTGcgcaaaggctgggatttatt
pri-mir-9-1 smiFISH_8	CCTCCTAAGTTTCGAGCTGGACTCAGTGgagagatggcttcaaacgca
pri-mir-9-1 smiFISH_9	CCTCCTAAGTTTCGAGCTGGACTCAGTGattgcaggctgatcattctc
pri-mir-9-1 smiFISH_10	CCTCCTAAGTTTCGAGCTGGACTCAGTGttcttgttgtctctgtctta
pri-mir-9-1 smiFISH_11	CCTCCTAAGTTTCGAGCTGGACTCAGTGagatgcaaagagtccattca
pri-mir-9-1 smiFISH_12	CCTCCTAAGTTTCGAGCTGGACTCAGTGaaccagcctgaactggttta
pri-mir-9-1 smiFISH_13	CCTCCTAAGTTTCGAGCTGGACTCAGTGctatgacttcaccagtctgt
pri-mir-9-1 smiFISH_14	CCTCCTAAGTTTCGAGCTGGACTCAGTGctcttgattgggtagcttaa
pri-mir-9-1 smiFISH_15	CCTCCTAAGTTTCGAGCTGGACTCAGTGtgatcacagtgctgagactg
pri-mir-9-1 smiFISH_16	CCTCCTAAGTTTCGAGCTGGACTCAGTGactgacgtaggttatctgta
pri-mir-9-1 smiFISH_17	CCTCCTAAGTTTCGAGCTGGACTCAGTGgctgaactgcaacctgaaa
pri-mir-9-1 smiFISH_18	CCTCCTAAGTTTCGAGCTGGACTCAGTGctttcaggtgtgtaatggga
pri-mir-9-1 smiFISH_19	CCTCCTAAGTTTCGAGCTGGACTCAGTGagtacagatctcaaggtggg
pri-mir-9-1 smiFISH_20	CCTCCTAAGTTTCGAGCTGGACTCAGTGagaggagaagaggagagggg
pri-mir-9-1 smiFISH_21	CCTCCTAAGTTTCGAGCTGGACTCAGTGgagagagggagacttgggag
pri-mir-9-1 smiFISH_22	CCTCCTAAGTTTCGAGCTGGACTCAGTGtggcttgctctcacaataaa
pri-mir-9-1 smiFISH_23	CCTCCTAAGTTTCGAGCTGGACTCAGTGagcactgatggaggccattt
pri-mir-9-1 smiFISH_24	CCTCCTAAGTTTCGAGCTGGACTCAGTGaaaatgctgcttaccttcca
pri-mir-9-1 smiFISH_25	CCTCCTAAGTTTCGAGCTGGACTCAGTGgaggagataatcacagctt
pri-mir-9-1 smiFISH_26	CCTCCTAAGTTTCGAGCTGGACTCAGTGagcaatgagagagactgggt
pri-mir-9-1 smiFISH_27	CCTCCTAAGTTTCGAGCTGGACTCAGTGacgcaccttttacaatagca
pri-mir-9-1 smiFISH_28	CCTCCTAAGTTTCGAGCTGGACTCAGTGtagatttgtgaggaggggtg
pri-mir-9-1 smiFISH_29	CCTCCTAAGTTTCGAGCTGGACTCAGTGagtgctgactactctaatgt
pri-mir-9-1 smiFISH_30	CCTCCTAAGTTTCGAGCTGGACTCAGTGattccgcgtattaaacgctt
pri-mir-9-1 smiFISH_31	CCTCCTAAGTTTCGAGCTGGACTCAGTGccctacggagagtgaaatga
pri-mir-9-1 smiFISH_32	CCTCCTAAGTTTCGAGCTGGACTCAGTGcattcagcttctccgtatcg
pri-mir-9-1 smiFISH_33	CCTCCTAAGTTTCGAGCTGGACTCAGTGcactccagaatgttgtcttg
pri-mir-9-1 smiFISH_34	CCTCCTAAGTTTCGAGCTGGACTCAGTGaaatatgcgcgtgcgagctg
pri-mir-9-1 smiFISH_35	CCTCCTAAGTTTCGAGCTGGACTCAGTGattcaggaccgatcgtaatg
pri-mir-9-1 smiFISH_36	CCTCCTAAGTTTCGAGCTGGACTCAGTGcctatcctcgagcatcgatg

Development • Supplementary information

Table S6. smiFISH Probe sequences for pri-mir-9-4

Probe sequence name	Probe sequence (5' to 3')
pri-mir-9-4 smiFISH_1	CCTCCTAAGTTTCGAGCTGGACTCAGTGctatcgatacccttgtggaa
pri-mir-9-4 smiFISH_2	CCTCCTAAGTTTCGAGCTGGACTCAGTGtggaaacgttgcgtttggtt
pri-mir-9-4 smiFISH_3	CCTCCTAAGTTTCGAGCTGGACTCAGTGacactgtcaagacactgcta
pri-mir-9-4 smiFISH_4	CCTCCTAAGTTTCGAGCTGGACTCAGTGatggttagcatttgttcgtg
pri-mir-9-4 smiFISH_5	CCTCCTAAGTTTCGAGCTGGACTCAGTGatgcacctcaaaaaactgct
pri-mir-9-4 smiFISH_6	CCTCCTAAGTTTCGAGCTGGACTCAGTGcagtatgtaggcgcttaagg
pri-mir-9-4 smiFISH_7	CCTCCTAAGTTTCGAGCTGGACTCAGTGtcttttatgtttggagctgg
pri-mir-9-4 smiFISH_8	CCTCCTAAGTTTCGAGCTGGACTCAGTGgcatcgcagttgtttacaat
pri-mir-9-4 smiFISH_9	CCTCCTAAGTTTCGAGCTGGACTCAGTGctcttgcgtttgcactttta
pri-mir-9-4 smiFISH_10	CCTCCTAAGTTTCGAGCTGGACTCAGTGtagtctaggtgttattgcgc
pri-mir-9-4 smiFISH_11	CCTCCTAAGTTTCGAGCTGGACTCAGTGtacgctcatattatgtgcgt
pri-mir-9-4 smiFISH_12	CCTCCTAAGTTTCGAGCTGGACTCAGTGcacacacacatacacgtggg
pri-mir-9-4 smiFISH_13	CCTCCTAAGTTTCGAGCTGGACTCAGTGatacagtctcactcactctt
pri-mir-9-4 smiFISH_14	CCTCCTAAGTTTCGAGCTGGACTCAGTGatggagctattgcattcact
pri-mir-9-4 smiFISH_15	CCTCCTAAGTTTCGAGCTGGACTCAGTGcagatgcagcttctgttgtg
pri-mir-9-4 smiFISH_16	CCTCCTAAGTTTCGAGCTGGACTCAGTGaaaactaacccatgtgcctt
pri-mir-9-4 smiFISH_17	CCTCCTAAGTTTCGAGCTGGACTCAGTGtaatctcaaggtctacgggg
pri-mir-9-4 smiFISH_18	CCTCCTAAGTTTCGAGCTGGACTCAGTGtcaaacgacagcactcacaa
pri-mir-9-4 smiFISH_19	CCTCCTAAGTTTCGAGCTGGACTCAGTGacatccagaacatccatttc
pri-mir-9-4 smiFISH_20	CCTCCTAAGTTTCGAGCTGGACTCAGTGccatccagcgttttacaaaa
pri-mir-9-4 smiFISH_21	CCTCCTAAGTTTCGAGCTGGACTCAGTGaattcaaagcgctccgaagt
pri-mir-9-4 smiFISH_22	CCTCCTAAGTTTCGAGCTGGACTCAGTGggattcgatgctctttgtag
pri-mir-9-4 smiFISH_23	CCTCCTAAGTTTCGAGCTGGACTCAGTGggaaacgcattaacgcagcg
pri-mir-9-4 smiFISH_24	CCTCCTAAGTTTCGAGCTGGACTCAGTGacagcttttggtattccagt
pri-mir-9-4 smiFISH_25	CCTCCTAAGTTTCGAGCTGGACTCAGTGacgtaaaatccacgtcttcc
pri-mir-9-4 smiFISH_26	CCTCCTAAGTTTCGAGCTGGACTCAGTGtatgtagccggaggcagatt
pri-mir-9-4 smiFISH_27	CCTCCTAAGTTTCGAGCTGGACTCAGTGcgtactgccagcgtactaac
pri-mir-9-4 smiFISH_28	CCTCCTAAGTTTCGAGCTGGACTCAGTGgtatccgcctgaataccaag
pri-mir-9-4 smiFISH_29	CCTCCTAAGTTTCGAGCTGGACTCAGTGcttgagaatgatcgcgaagt
pri-mir-9-4 smiFISH_30	CCTCCTAAGTTTCGAGCTGGACTCAGTGggaatgttgcccagcacaag
pri-mir-9-4 smiFISH_31	CCTCCTAAGTTTCGAGCTGGACTCAGTGacatcatcatcacgtctatg
pri-mir-9-4 smiFISH_32	CCTCCTAAGTTTCGAGCTGGACTCAGTGgtcatcccgaattcatacat
pri-mir-9-4 smiFISH_33	CCTCCTAAGTTTCGAGCTGGACTCAGTGaacacctcgatgaaccgatt
pri-mir-9-4 smiFISH_34	CCTCCTAAGTTTCGAGCTGGACTCAGTGagagatccgaccaatcggtt
pri-mir-9-4 smiFISH_35	CCTCCTAAGTTTCGAGCTGGACTCAGTGtatatgagaaccacgtgacc

Development • Supplementary information

Table S7. smiFISH Probe sequences for pri-mir-9-5

Probe sequence name	Probe sequence (5' to 3')
pri-mir-9-5 smiFISH_1	CCTCCTAAGTTTCGAGCTGGACTCAGTGacggaggagatcggtaatgc
pri-mir-9-5 smiFISH_2	CCTCCTAAGTTTCGAGCTGGACTCAGTGttagttttggccgagtggtg
pri-mir-9-5 smiFISH_3	CCTCCTAAGTTTCGAGCTGGACTCAGTGactgaatggagggatcgctc
pri-mir-9-5 smiFISH_4	CCTCCTAAGTTTCGAGCTGGACTCAGTGataacatcgcgtttgtttcg
pri-mir-9-5 smiFISH_5	CCTCCTAAGTTTCGAGCTGGACTCAGTGaggaaaaaaagtgcacgcgg
pri-mir-9-5 smiFISH_6	CCTCCTAAGTTTCGAGCTGGACTCAGTGgaggggaaaaatccgattcc
pri-mir-9-5 smiFISH_7	CCTCCTAAGTTTCGAGCTGGACTCAGTGgaaccagcagcgagattata
pri-mir-9-5 smiFISH_8	CCTCCTAAGTTTCGAGCTGGACTCAGTGtggcagcagcgatagatatc
pri-mir-9-5 smiFISH_9	CCTCCTAAGTTTCGAGCTGGACTCAGTGgctcctttggctctattaaa
pri-mir-9-5 smiFISH_10	CCTCCTAAGTTTCGAGCTGGACTCAGTGttacctcccaatctcacaaa
pri-mir-9-5 smiFISH_11	CCTCCTAAGTTTCGAGCTGGACTCAGTGtacaggccatcatttgcatt
pri-mir-9-5 smiFISH_12	CCTCCTAAGTTTCGAGCTGGACTCAGTGcctgtcgaataagaagcagt
pri-mir-9-5 smiFISH_13	CCTCCTAAGTTTCGAGCTGGACTCAGTGtatgagagttggagtgaggg
pri-mir-9-5 smiFISH_14	CCTCCTAAGTTTCGAGCTGGACTCAGTGaccggagcagttaacgttaa
pri-mir-9-5 smiFISH_15	CCTCCTAAGTTTCGAGCTGGACTCAGTGtggatatgctcgcaaccaaa
pri-mir-9-5 smiFISH_16	CCTCCTAAGTTTCGAGCTGGACTCAGTGggcaatggacttcacaattg
pri-mir-9-5 smiFISH_17	CCTCCTAAGTTTCGAGCTGGACTCAGTGtccaagtggagaagttgcta
pri-mir-9-5 smiFISH_18	CCTCCTAAGTTTCGAGCTGGACTCAGTGctccgtgtcttgtgtaagaa
pri-mir-9-5 smiFISH_19	CCTCCTAAGTTTCGAGCTGGACTCAGTGcacaacagtaatcccaggat
pri-mir-9-5 smiFISH_20	CCTCCTAAGTTTCGAGCTGGACTCAGTGgataacaactcgcttccaat
pri-mir-9-5 smiFISH_21	CCTCCTAAGTTTCGAGCTGGACTCAGTGctcatacagctagataacca
pri-mir-9-5 smiFISH_22	CCTCCTAAGTTTCGAGCTGGACTCAGTGgaggcagtttttactttcgg
pri-mir-9-5 smiFISH_23	CCTCCTAAGTTTCGAGCTGGACTCAGTGcagccagagtttcgatgagc
pri-mir-9-5 smiFISH_24	CCTCCTAAGTTTCGAGCTGGACTCAGTGagctactgacacaggctata
pri-mir-9-5 smiFISH_25	CCTCCTAAGTTTCGAGCTGGACTCAGTGtctaacatgttgtgtagcca
pri-mir-9-5 smiFISH_26	CCTCCTAAGTTTCGAGCTGGACTCAGTGttatagcaccaagtgaagcc
pri-mir-9-5 smiFISH_27	CCTCCTAAGTTTCGAGCTGGACTCAGTGcccgcgagctatgagaaata
pri-mir-9-5 smiFISH_28	CCTCCTAAGTTTCGAGCTGGACTCAGTGtttgtttgcttgagttaccg
pri-mir-9-5 smiFISH_29	CCTCCTAAGTTTCGAGCTGGACTCAGTGttcctccacaaggaaaagga
pri-mir-9-5 smiFISH_30	CCTCCTAAGTTTCGAGCTGGACTCAGTGccagtctattcgtctatttg
pri-mir-9-5 smiFISH_31	CCTCCTAAGTTTCGAGCTGGACTCAGTGttcacgtgtcagtttcagta
pri-mir-9-5 smiFISH_32	CCTCCTAAGTTTCGAGCTGGACTCAGTGaatagtgagcacgtgggttc
pri-mir-9-5 smiFISH_33	CCTCCTAAGTTTCGAGCTGGACTCAGTGcaaaggcgaccgagctgaaa
pri-mir-9-5 smiFISH_34	CCTCCTAAGTTTCGAGCTGGACTCAGTGatttctgtttgggatgccag
pri-mir-9-5 smiFISH_35	CCTCCTAAGTTTCGAGCTGGACTCAGTGgctctctctatatgtcctaa

Development • Supplementary information

Table S8. smiFISH Probe sequences for *her6*

**Probe sequence name**	Probe sequence (5' to 3')
her6 smiFISH_1	CCTCCTAAGTTTCGAGCTGGACTCAGTGtccatgatatcggcaggcat
her6 smiFISH_2	CCTCCTAAGTTTCGAGCTGGACTCAGTGgaccggagaagaggagtttt
her6 smiFISH_3	CCTCCTAAGTTTCGAGCTGGACTCAGTGtgggtttatcaggtgtagtg
her6 smiFISH_4	CCTCCTAAGTTTCGAGCTGGACTCAGTGagactttctgtgttccgaag
her6 smiFISH_5	CCTCCTAAGTTTCGAGCTGGACTCAGTGtcttttctccataatgggtt
her6 smiFISH_6	CCTCCTAAGTTTCGAGCTGGACTCAGTGtttcgttgattctcgctctt
her6 smiFISH_7	CCTCCTAAGTTTCGAGCTGGACTCAGTGattaacgttttcagctgacc
her6 smiFISH_8	CCTCCTAAGTTTCGAGCTGGACTCAGTGatcttttttcagagcatcca
her6 smiFISH_9	CCTCCTAAGTTTCGAGCTGGACTCAGTGggctttctcaagtttagagt
her6 smiFISH_10	CCTCCTAAGTTTCGAGCTGGACTCAGTGtcactgtcatctccaggatg
her6 smiFISH_11	CCTCCTAAGTTTCGAGCTGGACTCAGTGcgctgcatgtttctgagatg
her6 smiFISH_12	CCTCCTAAGTTTCGAGCTGGACTCAGTGtttagggcagcggtcatttg
her6 smiFISH_13	CCTCCTAAGTTTCGAGCTGGACTCAGTGttcccaagaacggtgggatc
her6 smiFISH_14	CCTCCTAAGTTTCGAGCTGGACTCAGTGattcactgaatccagctcgg
her6 smiFISH_15	CCTCCTAAGTTTCGAGCTGGACTCAGTGaaccgggtaacctcgttcat
her6 smiFISH_16	CCTCCTAAGTTTCGAGCTGGACTCAGTGtgttaaccccttcacatgtg
her6 smiFISH_17	CCTCCTAAGTTTCGAGCTGGACTCAGTGcgttgatctgtgtcatgcag
her6 smiFISH_18	CCTCCTAAGTTTCGAGCTGGACTCAGTGtgctgtgttggatagttcat
her6 smiFISH_19	CCTCCTAAGTTTCGAGCTGGACTCAGTGtgaaggatggatgaggaggc
her6 smiFISH_20	CCTCCTAAGTTTCGAGCTGGACTCAGTGggatctgaaccatgggttg
her6 smiFISH_21	CCTCCTAAGTTTCGAGCTGGACTCAGTGcgctaagaggcacaacgttg
her6 smiFISH_22	CCTCCTAAGTTTCGAGCTGGACTCAGTGgtcaaattggaggatgagcc
her6 smiFISH_23	CCTCCTAAGTTTCGAGCTGGACTCAGTGccatatactttagttgcgtc
her6 smiFISH_24	CCTCCTAAGTTTCGAGCTGGACTCAGTGttgccggcacaagctggaaa
her6 smiFISH_25	CCTCCTAAGTTTCGAGCTGGACTCAGTGcaaaaaggcgaactgtccgt
her6 smiFISH_26	CCTCCTAAGTTTCGAGCTGGACTCAGTGttggagcaaaggcagcgttg
her6 smiFISH_27	CCTCCTAAGTTTCGAGCTGGACTCAGTGtagactggaataacagggcc
her6 smiFISH_28	CCTCCTAAGTTTCGAGCTGGACTCAGTGgaaccggtgtgttggaattg
her6 smiFISH_29	CCTCCTAAGTTTCGAGCTGGACTCAGTGaaacggagtctgacgtgacg

Development • Supplementary information

Table S9. smiFISH Probe sequences for *her9*

Probe sequence name	Probe sequence (5' to 3')
her9 smiFISH _1	CCTCCTAAGTTTCGAGCTGGACTCAGTGcttctccatattatcggctg
her9 smiFISH _2	CCTCCTAAGTTTCGAGCTGGACTCAGTGcagcaataggtgatgctgtc
her9 smiFISH _3	CCTCCTAAGTTTCGAGCTGGACTCAGTGgcttgtcaggagtatgagat
her9 smiFISH _4	CCTCCTAAGTTTCGAGCTGGACTCAGTGagactttctatgctcgctgg
her9 smiFISH _5	CCTCCTAAGTTTCGAGCTGGACTCAGTGgcttttccatgattggcttt
her9 smiFISH _6	CCTCCTAAGTTTCGAGCTGGACTCAGTGaaggctctcgttgattctcg
her9 smiFISH _7	CCTCCTAAGTTTCGAGCTGGACTCAGTGgaatgagagtcttcagctgc
her9 smiFISH _8	CCTCCTAAGTTTCGAGCTGGACTCAGTGgctatcttttttaagagcat
her9 smiFISH _9	CCTCCTAAGTTTCGAGCTGGACTCAGTGtctccaatttagagtgtctg
her9 smiFISH _10	CCTCCTAAGTTTCGAGCTGGACTCAGTGgtcatctccagaatatcagc
her9 smiFISH _11	CCTCCTAAGTTTCGAGCTGGACTCAGTGtaaattgcgcaggtgcttga
her9 smiFISH _12	CCTCCTAAGTTTCGAGCTGGACTCAGTGaaggctgcgctcatctgaac
her9 smiFISH _13	CCTCCTAAGTTTCGAGCTGGACTCAGTGtacttgctgaggacgtttgt
her9 smiFISH _14	CCTCCTAAGTTTCGAGCTGGACTCAGTGcatgcactcgttgaatcctg
her9 smiFISH _15	CCTCCTAAGTTTCGAGCTGGACTCAGTGagagaaatcgagtcacctcg
her9 smiFISH _16	CCTCCTAAGTTTCGAGCTGGACTCAGTGtctgacctctgtattcactc
her9 smiFISH _17	CCTCCTAAGTTTCGAGCTGGACTCAGTGacaggtggttaagaagtcgc
her9 smiFISH _18	CCTCCTAAGTTTCGAGCTGGACTCAGTGcatcatctgtcccatacaac
her9 smiFISH _19	CCTCCTAAGTTTCGAGCTGGACTCAGTGcaggctgagggtagttcatg
her9 smiFISH _20	CCTCCTAAGTTTCGAGCTGGACTCAGTGagccaaatgagcctgttgag
her9 smiFISH _21	CCTCCTAAGTTTCGAGCTGGACTCAGTGgaagctgcacgtgaagaggc
her9 smiFISH _22	CCTCCTAAGTTTCGAGCTGGACTCAGTGccgttgatgggtaacgttga
her9 smiFISH _23	CCTCCTAAGTTTCGAGCTGGACTCAGTGtgagtttggaacccattgag
her9 smiFISH _24	CCTCCTAAGTTTCGAGCTGGACTCAGTGtggtgagaccgcttctgaag
her9 smiFISH _25	CCTCCTAAGTTTCGAGCTGGACTCAGTGagctggaatcctccaaagac
her9 smiFISH _26	CCTCCTAAGTTTCGAGCTGGACTCAGTGaaaagcaaactgtccgtccg
her9 smiFISH _27	CCTCCTAAGTTTCGAGCTGGACTCAGTGcaaacgctgggttggggata
her9 smiFISH _28	CCTCCTAAGTTTCGAGCTGGACTCAGTGaatgaccggagttgtggcag
her9 smiFISH _29	CCTCCTAAGTTTCGAGCTGGACTCAGTGcgcttgcgtttgcgtacaag
her9 smiFISH _30	CCTCCTAAGTTTCGAGCTGGACTCAGTGactggcgttgacagtcactg
her9 smiFISH _31	CCTCCTAAGTTTCGAGCTGGACTCAGTGcattccctggacaggagatg
her9 smiFISH _32	CCTCCTAAGTTTCGAGCTGGACTCAGTGcttgtggaacgcccgagaag
her9 smiFISH _33	CCTCCTAAGTTTCGAGCTGGACTCAGTGctgacaccaacgggactgac

Development • Supplementary information

Table S10. smiFISH Probe sequences for *neurog1*

Probe sequence name	Probe sequence (5' to 3')
neurog1 smiFISH_1	CCTCCTAAGTTTCGAGCTGGACTCAGTGtgctcttaaccctcaatcag
neurog1 smiFISH_2	CCTCCTAAGTTTCGAGCTGGACTCAGTGaagataatggcacgcgtctg
neurog1 smiFISH_3	CCTCCTAAGTTTCGAGCTGGACTCAGTGatcctgcagatagtttgtgt
neurog1 smiFISH_4	CCTCCTAAGTTTCGAGCTGGACTCAGTGgagatgcttgaggttttgca
neurog1 smiFISH_5	CCTCCTAAGTTTCGAGCTGGACTCAGTGtgataaccttattggtgggc
neurog1 smiFISH_6	CCTCCTAAGTTTCGAGCTGGACTCAGTGcggagtatacgatctccatt
neurog1 smiFISH_7	CCTCCTAAGTTTCGAGCTGGACTCAGTGtagtcacagcttgaggtttc
neurog1 smiFISH_8	CCTCCTAAGTTTCGAGCTGGACTCAGTGatcatccgtgtgcgaaaagg
neurog1 smiFISH_9	CCTCCTAAGTTTCGAGCTGGACTCAGTGtggagacgcaggtggttttc
neurog1 smiFISH_10	CCTCCTAAGTTTCGAGCTGGACTCAGTGttcttcttcacgacgtgcac
neurog1 smiFISH_11	CCTCCTAAGTTTCGAGCTGGACTCAGTGttaaggttgtgcatcctgtt
neurog1 smiFISH_12	CCTCCTAAGTTTCGAGCTGGACTCAGTGgcttctcaaagcatccaatg
neurog1 smiFISH_13	CCTCCTAAGTTTCGAGCTGGACTCAGTGtgtgtcgtcaggaaacgcag
neurog1 smiFISH_14	CCTCCTAAGTTTCGAGCTGGACTCAGTGgagtctcaattttggtcagc
neurog1 smiFISH_15	CCTCCTAAGTTTCGAGCTGGACTCAGTGatgtagttgtgagcgaagcg
neurog1 smiFISH_16	CCTCCTAAGTTTCGAGCTGGACTCAGTGatccggatggtctccgaaag
neurog1 smiFISH_17	CCTCCTAAGTTTCGAGCTGGACTCAGTGatgaagacgacgaggatgcc
neurog1 smiFISH_18	CCTCCTAAGTTTCGAGCTGGACTCAGTGggtctgagttgcagtaagac
neurog1 smiFISH_19	CCTCCTAAGTTTCGAGCTGGACTCAGTGtatccaaaatcgtccatggc
neurog1 smiFISH_20	CCTCCTAAGTTTCGAGCTGGACTCAGTGctgtacactacgtcggtttg
neurog1 smiFISH_21	CCTCCTAAGTTTCGAGCTGGACTCAGTGagatgctaggcacgaagttg
neurog1 smiFISH_22	CCTCCTAAGTTTCGAGCTGGACTCAGTGaccgacatgagaacgcttaa
neurog1 smiFISH_23	CCTCCTAAGTTTCGAGCTGGACTCAGTGtacatacttctggagattct
neurog1 smiFISH_24	CCTCCTAAGTTTCGAGCTGGACTCAGTGagtaacagtggtcttacact
neurog1 smiFISH_25	CCTCCTAAGTTTCGAGCTGGACTCAGTGgttgtgctcttggttctaat
neurog1 smiFISH_26	CCTCCTAAGTTTCGAGCTGGACTCAGTGaggcaaacagtgaataccta
neurog1 smiFISH_27	CCTCCTAAGTTTCGAGCTGGACTCAGTGtctcatttgcagactgtcat
neurog1 smiFISH_28	CCTCCTAAGTTTCGAGCTGGACTCAGTGcacgctccaaggaatgcaaa
neurog1 smiFISH_29	CCTCCTAAGTTTCGAGCTGGACTCAGTGattttctctaacggggttct
neurog1 smiFISH_30	CCTCCTAAGTTTCGAGCTGGACTCAGTGcaatctgccttgctttttaa
neurog1 smiFISH_31	CCTCCTAAGTTTCGAGCTGGACTCAGTGtgacttttcaccttggacag
neurog1 smiFISH_32	CCTCCTAAGTTTCGAGCTGGACTCAGTGtcagctttatcgctctacaa
neurog1 smiFISH_33	CCTCCTAAGTTTCGAGCTGGACTCAGTGaactgatttttcacgctcgt
neurog1 smiFISH_34	CCTCCTAAGTTTCGAGCTGGACTCAGTGttcagtctattgtcacagcg
neurog1 smiFISH_35	CCTCCTAAGTTTCGAGCTGGACTCAGTGcataaggccagatctttgtc
neurog1 smiFISH_36	CCTCCTAAGTTTCGAGCTGGACTCAGTGtttcttcgggtcaaaataca
neurog1 smiFISH_37	CCTCCTAAGTTTCGAGCTGGACTCAGTGggatcagttggacagatgag
neurog1 smiFISH_38	CCTCCTAAGTTTCGAGCTGGACTCAGTGcatgagagctggttaactgt
neurog1 smiFISH_39	CCTCCTAAGTTTCGAGCTGGACTCAGTGagaaaagtggtgggaaagcc
neurog1 smiFISH_40	CCTCCTAAGTTTCGAGCTGGACTCAGTGcgtacaaacatgtttgcacc

Development • Supplementary information

Table S11. smiFISH Probe sequences for *atoh1a*

Probe sequence name	Probe sequence (5' to 3')
atoh1a smiFISH_1	CCTCCTAAGTTTCGAGCTGGACTCAGTGggtgaaggggatttctttac
atoh1a smiFISH_2	CCTCCTAAGTTTCGAGCTGGACTCAGTGagatttctcttcttacacct
atoh1a smiFISH_3	CCTCCTAAGTTTCGAGCTGGACTCAGTGtacagggacggatattttgc
atoh1a smiFISH_4	CCTCCTAAGTTTCGAGCTGGACTCAGTGttgggaggaaagtttgtggc
atoh1a smiFISH_5	CCTCCTAAGTTTCGAGCTGGACTCAGTGatccattctgttggtttgtg
atoh1a smiFISH_6	CCTCCTAAGTTTCGAGCTGGACTCAGTGttcaaccacctctcttgtat
atoh1a smiFISH_7	CCTCCTAAGTTTCGAGCTGGACTCAGTGaagctcgaatgctggacgtc
atoh1a smiFISH_8	CCTCCTAAGTTTCGAGCTGGACTCAGTGtgaagttggaggcagaggac
atoh1a smiFISH_9	CCTCCTAAGTTTCGAGCTGGACTCAGTGcgtactttgacaggactctt
atoh1a smiFISH_10	CCTCCTAAGTTTCGAGCTGGACTCAGTGgttggtggatttgctggatg
atoh1a smiFISH_11	CCTCCTAAGTTTCGAGCTGGACTCAGTGttcaatccgtgcattcttcg
atoh1a smiFISH_12	CCTCCTAAGTTTCGAGCTGGACTCAGTGcaaaggctgggatgacactg
atoh1a smiFISH_13	CCTCCTAAGTTTCGAGCTGGACTCAGTGttggagagtttcttgtcgtt
atoh1a smiFISH_14	CCTCCTAAGTTTCGAGCTGGACTCAGTGttgatgtagatctgggccat
atoh1a smiFISH_15	CCTCCTAAGTTTCGAGCTGGACTCAGTGctgtagtaagtcggacaggg
atoh1a smiFISH_16	CCTCCTAAGTTTCGAGCTGGACTCAGTGtttctaacacgttggcatgc
atoh1a smiFISH_17	CCTCCTAAGTTTCGAGCTGGACTCAGTGctcgtactggtacgggtaac
atoh1a smiFISH_18	CCTCCTAAGTTTCGAGCTGGACTCAGTGgtcttgctccatgaaagagt
atoh1a smiFISH_19	CCTCCTAAGTTTCGAGCTGGACTCAGTGcgaaccagacttgctcgttc
atoh1a smiFISH_20	CCTCCTAAGTTTCGAGCTGGACTCAGTGaccgaggcgagtctttactg
atoh1a smiFISH_21	CCTCCTAAGTTTCGAGCTGGACTCAGTGcgagtgaggcgagaactctc
atoh1a smiFISH_22	CCTCCTAAGTTTCGAGCTGGACTCAGTGgtttcgtctgagtcactgaa
atoh1a smiFISH_23	CCTCCTAAGTTTCGAGCTGGACTCAGTGgacagctcgtcttcactctg
atoh1a smiFISH_24	CCTCCTAAGTTTCGAGCTGGACTCAGTGtttcttaaaaagcgcggcgc
atoh1a smiFISH_25	CCTCCTAAGTTTCGAGCTGGACTCAGTGtgacacgattgagggacagt
atoh1a smiFISH_26	CCTCCTAAGTTTCGAGCTGGACTCAGTGaagcaacccattacaaagcc
atoh1a smiFISH_27	CCTCCTAAGTTTCGAGCTGGACTCAGTGtctattggcgtcagacacaa
atoh1a smiFISH_28	CCTCCTAAGTTTCGAGCTGGACTCAGTGgtcaatgtcacgagaaggca
atoh1a smiFISH_29	CCTCCTAAGTTTCGAGCTGGACTCAGTGttatgcatgacattcgagcc
atoh1a smiFISH_30	CCTCCTAAGTTTCGAGCTGGACTCAGTGgtctcaaaacagtttgcgga
atoh1a smiFISH_31	CCTCCTAAGTTTCGAGCTGGACTCAGTGaggtccatgacaatcatgtg
atoh1a smiFISH_32	CCTCCTAAGTTTCGAGCTGGACTCAGTGgtcgcaaattgttacagggt
atoh1a smiFISH_33	CCTCCTAAGTTTCGAGCTGGACTCAGTGccgttttttaaagtgcaagt
atoh1a smiFISH_34	CCTCCTAAGTTTCGAGCTGGACTCAGTGgaatatttctcaagcctacg
atoh1a smiFISH_35	CCTCCTAAGTTTCGAGCTGGACTCAGTGcacatcatttcttgttccat
atoh1a smiFISH_36	CCTCCTAAGTTTCGAGCTGGACTCAGTGattggcattacttctacata
atoh1a smiFISH_37	CCTCCTAAGTTTCGAGCTGGACTCAGTGgcagaacacaacttctttgc
atoh1a smiFISH_38	CCTCCTAAGTTTCGAGCTGGACTCAGTGatagcttacttcagctacag
atoh1a smiFISH_39	CCTCCTAAGTTTCGAGCTGGACTCAGTGgctcatttccaatgtaacac

Development • Supplementary information

Table S12. smiFISH Probe sequences for *ascl1a*

Probe sequence name	Probe sequence (5' to 3')
ascl1a smiFISH_1	CCTCCTAAGTTTCGAGCTGGACTCAGTGtctcgacctgttctgagttg
ascl1a smiFISH_2	CCTCCTAAGTTTCGAGCTGGACTCAGTGgttcacgtgtggcttcaatg
ascl1a smiFISH_3	CCTCCTAAGTTTCGAGCTGGACTCAGTGaaagttttctttggactgcc
ascl1a smiFISH_4	CCTCCTAAGTTTCGAGCTGGACTCAGTGtccatttcgcggagtcaaaa
ascl1a smiFISH_5	CCTCCTAAGTTTCGAGCTGGACTCAGTGtggtttacgcttatttccat
ascl1a smiFISH_6	CCTCCTAAGTTTCGAGCTGGACTCAGTGaagcaagcaggtggcatgaa
ascl1a smiFISH_7	CCTCCTAAGTTTCGAGCTGGACTCAGTGtgagttggatgctctgagag
ascl1a smiFISH_8	CCTCCTAAGTTTCGAGCTGGACTCAGTGttgacgcggacttgttgctg
ascl1a smiFISH_9	CCTCCTAAGTTTCGAGCTGGACTCAGTGattgagtcttcttttgcacc
ascl1a smiFISH_10	CCTCCTAAGTTTCGAGCTGGACTCAGTGgggtaagctgtagccgaaac
ascl1a smiFISH_11	CCTCCTAAGTTTCGAGCTGGACTCAGTGcaaagccgttgttcacaagc
ascl1a smiFISH_12	CCTCCTAAGTTTCGAGCTGGACTCAGTGctccattgggaacgtgttcg
ascl1a smiFISH_13	CCTCCTAAGTTTCGAGCTGGACTCAGTGctttgctcatcttcttgttg
ascl1a smiFISH_14	CCTCCTAAGTTTCGAGCTGGACTCAGTGgtagttttgggagatggtgg
ascl1a smiFISH_15	CCTCCTAAGTTTCGAGCTGGACTCAGTGgccatagagttcatgtcatt
ascl1a smiFISH_16	CCTCCTAAGTTTCGAGCTGGACTCAGTGctcatccgatgagtatgagg
ascl1a smiFISH_17	CCTCCTAAGTTTCGAGCTGGACTCAGTGttgttcttctggactcagag
ascl1a smiFISH_18	CCTCCTAAGTTTCGAGCTGGACTCAGTGaaaccagttggtgaagtcca
ascl1a smiFISH_19	CCTCCTAAGTTTCGAGCTGGACTCAGTGagtttccttttacgaacgct
ascl1a smiFISH_20	CCTCCTAAGTTTCGAGCTGGACTCAGTGccaagcgagtgctgatattt
ascl1a smiFISH_21	CCTCCTAAGTTTCGAGCTGGACTCAGTGttgtgttcttggaggacatc
ascl1a smiFISH_22	CCTCCTAAGTTTCGAGCTGGACTCAGTGcttggctctttgacactcgg
ascl1a smiFISH_23	CCTCCTAAGTTTCGAGCTGGACTCAGTGatagatttcttgggcgagtg
ascl1a smiFISH_24	CCTCCTAAGTTTCGAGCTGGACTCAGTGggtgtcgtggaaagtctttt
ascl1a smiFISH_25	CCTCCTAAGTTTCGAGCTGGACTCAGTGcaacgtttgcttgttgttgt
ascl1a smiFISH_26	CCTCCTAAGTTTCGAGCTGGACTCAGTGagggcaaaccttctttgatt
ascl1a smiFISH_27	CCTCCTAAGTTTCGAGCTGGACTCAGTGagagttttgagagaggggtc
ascl1a smiFISH_28	CCTCCTAAGTTTCGAGCTGGACTCAGTGaattcgccaagttggaagca
ascl1a smiFISH_29	CCTCCTAAGTTTCGAGCTGGACTCAGTGcggcaggctataggtcaaaa
ascl1a smiFISH_30	CCTCCTAAGTTTCGAGCTGGACTCAGTGctgcgatgcatttgagacta
ascl1a smiFISH_31	CCTCCTAAGTTTCGAGCTGGACTCAGTGctccacaaccgtaaagggaa
ascl1a smiFISH_32	CCTCCTAAGTTTCGAGCTGGACTCAGTGttgagcattacactcctcta
ascl1a smiFISH_33	CCTCCTAAGTTTCGAGCTGGACTCAGTGataagacacgttggtgctga
ascl1a smiFISH_34	CCTCCTAAGTTTCGAGCTGGACTCAGTGtcactctgagtcacattaca
ascl1a smiFISH_35	CCTCCTAAGTTTCGAGCTGGACTCAGTGggacaatagctgcataacct
ascl1a smiFISH_36	CCTCCTAAGTTTCGAGCTGGACTCAGTGgagacacaaaacacctccgt
ascl1a smiFISH_37	CCTCCTAAGTTTCGAGCTGGACTCAGTGgcaaagtggaacaggcagtg
ascl1a smiFISH_38	CCTCCTAAGTTTCGAGCTGGACTCAGTGtgactgcaacacgtaaagca
ascl1a smiFISH_39	CCTCCTAAGTTTCGAGCTGGACTCAGTGttagcaggatggcttatcac
ascl1a smiFISH_40	CCTCCTAAGTTTCGAGCTGGACTCAGTGttggcattcattaagagcgc

Development • Supplementary information

Table S13. CRISPR/Cas9 Target sequences for pre-mir-9-1 and primers used to generate respective sgRNA. PAM region is Highlighted in red.

sgRNA Number	Target sequence 5'-3' with PAM	CRISPRscan Primer 5'-3'
1	GGACGGGTGGCCGGAGGGGTTGG	taatacgactcactataGGACGGGTGGCCGGAGGGGTgttttagagctagaa
2	AAGGTGGGGTGATGCCTTTCAGG	taatacgactcactataGGGGTGGGGTGATGCCTTTCgttttagagctagaa
3	AGAGAGAGGGAGACTTGGGAGGG	taatacgactcactataGGAGAGAGGGAGACTTGGGAgttttagagctagaa

Table S14. The parameter values used for the mathematical model. Highlighted in grey are the additional parameters used for the extended model. α_X, μ_X, α_h and μ_h parameters were used in both, adaptation and extended, models.

Parameter	Value
h_1	3.0
h_2	3.0
h_3	3.0
P_1 (low, high)	2.0, 10.0
p_2	1.0
p_3	1.0
α_X	0.1
μ_X	0.1
α_h	6.0
μ_h	2.0
α_Y	30.0
β_Y	100.0
μ_Y	10.0

Development • Supplementary information
