# Supplementary material for: Sequential and additive expression of miR-9 precursors control timing of neurogenesis
Source: Development. 2022 Oct 3;149(19):dev200474. doi: 10.1242/dev.200474 (PMC9641661; doi:10.1242/dev.200474)
Supplement: Supplementary file 1 [file develop-149-200474_review_history.pdf]

Sequential and additive expression of miR-9 precursors control timing of neurogenesis

Ximena Soto, Joshua Burton, Cerys S. Manning, Thomas Minchington, Robert Lea, Jessica Lee, Jochen Kursawe, Magnus Rattray and Nancy Papalopulu
DOI: 10.1242/dev.200474

Editor: Steve Wilson

Review timeline

Submission to Review Commons:	22 July 2020
Submission to Development:	15 December 2021
Editorial decision:	24 February 2022
First revision received:	24 June 2022
Editorial decision:	28 July 2022
Second revision received:	24 August 2022
Accepted:	26 August 2022

Reviewer 1

Evidence, reproducibility and clarity

In their study entitled 'Sequential onset and concurrent expression of miR-9 genomic loci in single cells contributes to the temporal increase of mature miR-9 in zebrafish neurogenesis', Soto et al show that different precursors of the micro-RNA miR-9 (pri-miR-9) have distinct spatial and temporal profiles and speculate that this leads to a temporal increase of overall miR-9 between 24hpf and 48hpf in the hindbrain. While this work is technically appealing, and assessment of the distribution of different pri micro RNAs has not been done in many developmental contexts, the findings seem to be rather preliminary and a lot of the interpretation is correlative and speculative. For example, while the authors state that Her6 switched from noisy to oscillatory at the same time as miR-9 onset, this co-incident is not further explored. Many such statements can be found in the text.

Some specific comments:

-Figure 1 could use a more quantitative assessment of the different pri-miR-9 constructs, maybe also at shorter timescales as currently is seem to be bimodal between no and full expression of the different pri-miR-9 versions.

-Why do the authors ask their questions in what seems to be the most complicated system with 7 paralogues of pri-miR-9...? How do their findings relate to systems with less paralogues?

-In Figure 4 the interpretation of the results is not clear as the authors see some pri-miR-9 sometimes in the same cells and other times exclusive between different cells. What difference would that make for overall miR-9 expression in specific regions?

-Overall, from a manuscript point of view, the message of the study could be more clearly developed.

-The discussion based on the stainings presented in the results is very speculative. Many interesting and important questions are raised here but none of has been addressed in the results section.

© 2022. Published by The Company of Biologists under the terms of the Creative Commons Attribution License (https://creativecommons.org/licenses/by/4.0/).

-In the last sentence of the discussion the authors state: 'Therefore, an added benefit of our work is that the detailed characterisation we have described here will enable the selection of the correct genomic locus for genetic manipulation of miR-9 production, depending on the precise research question'. The authors might consider to focus their manuscript on this point and develop it more into a technical report.

-Side note: Figure 1A was already published in the authors previous EMBO paper 2020, DOI10.15252/embj.2019103558 featuring the same first and last author (Figure 3A). While the images are different, so no duplication there, this should be noted in the text.

Significance

Currently this manuscript is at a descriptive level and many of the presented data seems premature considering the conclusions drawn. It might fit better as a focused technical report or significantly more experiments are needed.

This is from the perspective of a developmental biologist working in zebrafish.

Reviewer 2

Evidence, reproducibility and clarity

Major points

1)Description of expression pattern of each pri-miR and images are not consistent between figures. For example, in line 198~200, "pri-miR-9-5 is more restricted in its expression to the middle-ventral progenitor region of the ventricular zone, while pri-miR-9-1 was more broadly expressed toward the dorsal progenitor region (Fig. 3d, e)". In Figure 3c (upper panels), however, Pri-miR-9-5 looks more laterally expressed as compared to pri-miR-9-1. Same as in Figure4e.

2)line 216: "different miR-9 paralogues are concurrently transcriptionally active in the same cells". To show this in more convincing way, distribution of pri-miR-9-1, 4, 5 signals should be quantified according to the location (coordinate) within coronal sections from 32 hpf to 48 hpf.

3)line248: "expression levels of pri-miR-9-5 is consistently reduced (Fig. 5e. dashed line) in the regions where the zebrafish Hes1orthologues, Her6/Her9, are highly expressed (Fig. 5c)". This is not necessarily clear from Figure 5 c-e. Try double staining with miR-9-5 and Her6 or 9.

4)line 253: "We also observed expression of pri-miR-9-4 narrowed to the ventral progenitor region of the ventricular zone (Fig. 5d, dashed line), this can be explained by its higher number of E-boxes when compared to pri-miR-9-1".
Authors inferred that E Boxes mediate transcriptional repression of miR-9-4, because miR-9-4 and 5 showed similar expression pattern, but quite different number of E boxes belonging to them. Authors should try luciferase assay to show E box mediates repression by her6/9.

Minor points

1)line193, 194: pri-miR-4, pri-miR-5 ◊ pri-miR-9-4, 9-5.

2)line 192~: Describe well miR-9-4 expression pattern in the text.

3)Number of slices and brains used for staining should be indicated in methods or in figure legends.

Significance

From this study, new insight that microRNAs have more diverse transcriptional regulation mechanism than ever thought thus enables being versatile in multi region of the brain was obtained.

© 2022. Published by The Company of Biologists under the terms of the Creative Commons Attribution License (https://creativecommons.org/licenses/by/4.0/).

Referees Cross-Commenting

Reviewer #1's Review

-In Figure 4 the interpretation of the results is not clear as the authors see some pri-miR-9 sometimes in the same cells and other times exclusive between different cells. What difference would that make for overall miR-9 expression in specific regions?

I agree with this comment. This figure should be presented in more convincing manner: count the number of positive cells for each pri-miR in the area and classify the expressing pattern according to colocalization for at least three different embryos.

Reviewer #3's Review

FISH is more a qualitative than a quantitative approach, at the end it appears that almost all miR-9 genes are expressed simultaneously with the exception of form 1 and form 4-5, which are further detailed. It is therefore difficult to draw a certain conclusion on how much each isoform contributes, but it seems likely that all contribute in some way

-the single cell resolution analysis is limited to FISH and not really quantitative.

I agree with this comment. qPCR for each pri-miRs from the slice shown in Fig. 4 could be a more quantitative approach.

-the characterization of the E or N boxes is rather superficial and has more of a preliminary rather than a mechanistic character. There is no validation that the number of binding sites actually contribute or determine to the expression pattern or that transcription factors such as Neurogenin are really involved

I agree. To obtain a clue to answer this question, I suggested to do luciferase assay.

Reviewer 3

Evidence, reproducibility and clarity

The paper by Soto et al, deals with the expression and the regulation of miR-9 during zebrafish neurogenesis. Authors provide a detailed analysis of the spatiotempral profile of expression of the (7) different genes of miR-9 by fluorescent in situ hybridization in a time window betwenn 24 and 48 hpf.
It emerged that some the pri-miR-9 transcripts are expressed in different brain area, in different cells or at different time. Therefore, the different miR-9 genes together contribute to define the spatio-temporal expression pattern of miR-9, and contribute incrementally to its accumulation over time during neurogenesis.

However there are some objective limits:

-the work relies exclusively on the FISH technique, which is very powerful to follow the expression of individual genes but should be corroborated with an independent approach

-FISH is more a qualitative than a quantitative approach, at the end it appears that almost all miR-9 genes are expressed simultaneously with the exception of form 1 and form 4-5, which are further detailed. It is therefore difficult to draw a certain conclusion on how much each isoform contributes, but it seems likely that all contribute in some way

-the single cell resolution analysis is limited to FISH and not really quantitative

-the characterization of the E or N boxes is rather superficial and has more of a preliminary rather than a mechanistic character. There is no validation that the number of binding sites actually contribute or determine to the expression pattern or that transcription factors such as Neurogenin are really involved

© 2022. Published by The Company of Biologists under the terms of the Creative Commons Attribution License (https://creativecommons.org/licenses/by/4.0/).

- There is no genetic/biochemical/molecular approach to mechanistic dissection of the involvement of various miR-9 genes and their contributions to total miR-9 levels or to regulation of target genes. This could help give a more mechanistic and less descriptive aspect to the work.

Significance

This work is technically excellent, but of limited significance.

The analysis of the expression of the various genes is very detailed, with excellent image quality and a precise description. However, significance is limited to the purely descriptive nature of the work.

No mechanistic hypothesis emerges. There is just an in silico correlation with E or N boxes, which at the end does not explain neither the pattern of expression of the different miR-9 genes, nor the different level of accumulation of the mature miR-9

The whole work is actually a starting point for the subsequent dissection of the mechanisms of regulation of miR-9 expression or the dissection of the specific impact of different genes to miR-9 functions, which in my opinion would be of great interest.

Author response to reviewers' comments

We thank the reviewers for their insightful and constructive comments. We have taken on board fully the reviewer's criticisms and we have put more than a year's additional work to improve the manuscript including substantial new experimentation. We hope that the new manuscript fully addresses all of the reviewers' comments. To reflect the addition of functional experiments, the title has changed from "Sequential onset and concurrent expression of miR-9 genomic loci in single cells contributes to the temporal increase of mature miR-9 in zebrafish neurogenesis" to "Timing of neurogenesis through sequential accumulation of miR-9 from additive expression of multiple alleles" and four new co-authors who participated in improving the paper have been added. We provide a point-by-point account of our revisions below:

Reviewer 1

Evidence, reproducibility and clarity

In their study entitled 'Sequential onset and concurrent expression of miR-9 genomic loci in single cells contributes to the temporal increase of mature miR-9 in zebrafish neurogenesis', Soto et al show that different precursors of the micro-RNA miR-9 (pri-miR-9) have distinct spatial and temporal profiles and speculate that this leads to a temporal increase of overall miR-9 between 24hpf and 48hpf in the hindbrain. While this work is technically appealing, and assessment of the distribution of different pri micro RNAs has not been done in many developmental contexts, the findings seem to be rather preliminary and a lot of the interpretation is correlative and speculative. For example, while the authors state that Her6 switched from noisy to oscillatory at the same time as miR-9 onset, this co-incident is not further explored. Many such statements can be found in the text.

In the revised manuscript, we have addressed this criticism by using quantitative Taqman RT-qPCR analysis of mature miR-9 expression at stages 25, 30, 37, 42, 48hpf, of dissected hindbrains, to show the temporal increase of mature miR-9 during development. Furthermore, we performed quantitative SYBR-green RT-qPCR analysis in the hindbrain for 7 pri-mir-9s. Both RT-qPCR experiments, mature miR-9 and 7 pri-mir-9s, are now presented alongside the in-situ hybridisation in Figure 1.

© 2022. Published by The Company of Biologists under the terms of the Creative Commons Attribution License (https://creativecommons.org/licenses/by/4.0/).

The evidence that during this time window, Her6 switches from noisy to oscillatory to downregulated is shown in our previous paper (Soto et al., 2020). Furthermore, Bioinformatic analysis of RNAseq data at stages 24, 30, 36 and 48hpf presented in Fig. S1 supports that upregulation of pri-mir-9 expression coincides temporally with a downregulation of several her family members. This agrees with our previous findings that an increase of miR-9 can drive the mouse homologue Hes1 expression in a different dynamical space, first, from stable to oscillatory and upon further increase, from oscillatory to declining (Goodfellow et al., 2014).

*Some specific comments:***

-Figure 1 could use a more quantitative assessment of the different pri-miR-9 constructs, maybe also at shorter timescales as currently is seem to be bimodal between no and full expression of the different pri-miR-9 versions.

As mentioned above we have performed quantitative RT-qPCR analysis in the hindbrain for 7 pri-mir-9s over time, shown in new Fig. 1D and E. We have also rearranged the order of the in-situ hybridisation data from early to late to show better the temporal progression of expression between different pri-mir-9s.

-Why do the authors ask their questions in what seems to be the most complicated system with 7 paralogues of pri-miR-9...? How do their findings relate to systems with less paralogues?

We have chosen to do this work in the zebrafish, because it is the system of choice for live imaging. As such it has been instrumental for imaging cell state transitions at the single cell level and in the intact neural tissue (Soto et al., 2020). Thus, it was important to characterise the contribution of different pri-mir-9 in the zebrafish system even though it is more complex than other systems that have less paralogues.

To make sure that we are not looking at a zebrafish-specific phenomenon we have chosen the pri-mir-9 to be analysed not only based on their temporal profile but also based on their wide distribution on the mir-9 phylogenetic tree (pri-mir-9-5, 9-4 and 9-1; Fig. S3D).

In relation to other systems, we have included a recent reference in the mouse where paralogue miRs have phenotypes in the vertebral column which are compatible with a quantitative mechanism that we describe here (as well as qualitative differences) (Amin et al., 2021). These papers indirectly support our findings.

-In Figure 4 the interpretation of the results is not clear as the authors see some pri-miR-9 sometimes in the same cells and other times exclusive between different cells. What difference would that make for overall miR-9 expression in specific regions?

Our interpretation of this result is that some expression is qualitative different (i.e. there is some spatial specificity in the expression of pri-mir-9s, compatible with different paralogues targeting region-specific genes; for example her6 versus her9). Importantly there is also a quantitative aspect, revealed for the first time in this paper whereby two or more pri-mir-9s are expressed in the same cells in a sequential but additive manner. We have re-written the discussion to make the difference between a qualitative and quantitative mechanisms clearer.

-Overall, from a manuscript point of view, the message of the study could be more clearly developed.

We have taken this point fully on board and we have re-written the abstract, introduction, and discussion, and we have changed the title. Together with the additional experimental and theoretical data (in Figures 5 and 6) the message is now more developed and presented in a clearer way for the reader.

-The discussion based on the stainings presented in the results is very speculative. Many interesting and important questions are raised here but none of has been addressed in the results section.

© 2022. Published by The Company of Biologists under the terms of the Creative Commons Attribution License (https://creativecommons.org/licenses/by/4.0/).

We have strengthened our conclusions presented in the discussion based on new staining, quantitation and functional experiments. Briefly, we have used CRISPR/Cas9 knock-down experiment of the late pri-mir-9-1, which we then analysed with a panel of neuronal markers. We found that this knock-down resulted in a specific reduction of late differentiating neurons. This experiment is shown in new Figure 5. Thus, we now show that the late increase of miR-9, contributed by the late pri-mir-9 paralogue, 9-1, is specifically needed to mediate late neurogenesis.

Second, we have used mathematical modelling to ask whether not only the increase of miR-9, but also the way by which the increase is achieved, may be functionally important. This was motivated by the RT-qPCR data for mature miR-9 in Figure 1 and for pri-mir-9-1, 9-4 and 9-5 in Figure 6, which show that the increase during development takes place sharply, in steps. We used theory to predict what a network that shows adaptation to fluctuations in miR-9 would look like. This simple network is based on interactions that are currently known plus some hypothetical, predicted, components. Mathematical modelling then shows that this network shows adaptation of its output (her6 level) to linear increase or fluctuations of input (miR-9). Interestingly, the stepwise increase of miR-9 prevents adaptation of this network and results in persistent downregulation of her6, which is necessary for cells to differentiate. These new data are shown in Figure 6. We think this part of the work is particularly exciting because it reconciles the need of gene expression networks for robustness to external fluctuations, with the need to respond to changes in the input so that they can reach a new state which is particularly important for developmental transitions.

-In the last sentence of the discussion the authors state: 'Therefore, an added benefit of our work is that the detailed characterisation we have described here will enable the selection of the correct genomic locus for genetic manipulation of miR-9 production, depending on the precise research question'. The authors might consider to focus their manuscript on this point and develop it more into a technical report.

We appreciate the constructive spirit of the suggestion, but we have taken the alternative path of strengthening the paper as a primary research paper rather than as a technical report.

-Side note: Figure 1A was already published in the authors previous EMBO paper 2020, DOI10.15252/embj.2019103558 featuring the same first and last author (Figure 3A). While the images are different, so no duplication there, this should be noted in the text.

The image is indeed different, but the EMBO paper will be noted in the text.

Significance

Currently this manuscript is at a descriptive level and many of the presented data seems premature considering the conclusions drawn. It might fit better as a focused technical report or significantly more experiments are needed.

This is from the perspective of a developmental biologist working in zebrafish.

We have added significantly more experiments to the paper, including functional experiments in Figure 5 and mathematical modelling in Figure 6, as we mentioned above and again below. The conclusions are now better supported by the data and we hope that the paper can no longer be deemed descriptive or preliminary.

Reviewer 2

Evidence, reproducibility and clarity

Major points

1) Description of expression pattern of each pri-miR and images are not consistent between figures. For example, in line 198~200, "pri-miR-9-5 is more restricted in its expression to the middle-ventral progenitor region of the ventricular zone, while pri-miR-9-1 was more broadly

© 2022. Published by The Company of Biologists under the terms of the Creative Commons Attribution License (https://creativecommons.org/licenses/by/4.0/).

expressed toward the dorsal progenitor region (Fig. 3d, e)". In Figure 3c (upper panels), however, Pri-miR-9-5 looks more laterally expressed as compared to pri-miR-9-1. Same as in Figure4e

Figure 3c is a maximum intensity projection of a longitudinal Z stack and we think these can be misleading with regards to the D-V (M-L) aspect of expression. Therefore, we are basing our middle, ventral and dorsal description based on transverse sections. In transverse sections it is clear that pri-mir-9-5 is more restricted in the D-V (M-L) dimension than pri-mir-9-1 (see transverse view at 48hpf, last panel in new Fig. 2D).
Please note that we have rearranged and combined previous figures 2 and 3 into one new figure (figure 2) to allow better side by side comparison between pri-mir-9-4 and pri-mir-9-5, each in a double in situ hybridisation experiments with pri-mir-9-1, in two development stages, and longitudinal as well as transverse sections.

2)line 216: "different miR-9 paralogues are concurrently transcriptionally active in the same cells". To show this in more convincing way, distribution of pri-miR-9-1, 4, 5 signals should be quantified according to the location (coordinate) within coronal sections from 32 hpf to 48 hpf.

We have taken on board this suggestion and we have performed better quality and more detailed analysis, Specifically, we have analysed the expression of pri-mir-9-1, 9-4 and 9-5 with triple smiFISH in transverse hindbrain sections. This analysis highlights the transcription sites for each pri-mir-9 and is a quantitative method as the number of spots can be counted. Together with phalloidin labelling for cell membranes, this allowed us to produce high quality data where the co-expression of 2 or 3 pri-mir-9s is shown in detail and furthermore has been quantitated as shown in new Figure 3 and new Figure 4, in 3 developmental stages (30hpf, 36-37hpf and 48hpf).

3)line248: "expression levels of pri-miR-9-5 is consistently reduced (Fig. 5e. dashed line) in the regions where the zebrafish Hes1orthologues, Her6/Her9, are highly expressed (Fig. 5c)". This is not necessarily clear from Figure 5 c-e.

We agree that this was not clear by the comparison shown and we thank you for prompting us to look at this more closely. We have now done a direct comparison of *her6*/*her9*/pri-mir-9-5 expression with a triple smiFISH staining (shown in new Figure 4F). The result shows that pri-mir-9-5, *her6* and *her9* are broadly co-expressed but cells that have high *her6* or *her9* expression tend to have less pri-mir-9 expression.

4)line 253: "We also observed expression of pri-miR-9-4 narrowed to the ventral progenitor region of the ventricular zone (Fig. 5d, dashed line), this can be explained by its higher number of E-boxes when compared to pri-miR-9-1".
Authors inferred that E Boxes mediate transcriptional repression of miR-9-4, because miR-9-4 and 5 showed similar expression pattern, but quite different number of E boxes belonging to them. Authors should try luciferase assay to show E box mediates repression by her6/9.

We have removed all of the E-box part of the data from the revised paper as it was not a key finding the paper and it detracted from main message.

Minor points

1)line193, 194: pri-miR-4, pri-miR-5 ◊ pri-miR-9-4, 9-5.

This will be corrected.

2)line 192~: Describe well miR-9-4 expression pattern in the text.

The relevant text will be revised

3)Number of slices and brains used for staining should be indicated in methods or in figure legends.

This information will be added.

© 2022. Published by The Company of Biologists under the terms of the Creative Commons Attribution License (https://creativecommons.org/licenses/by/4.0/).

Significance

From this study, new insight that microRNAs have more diverse transcriptional regulation mechanism than ever thought thus enables being versatile in multi region of the brain was obtained.

Referees Cross-Commenting

Reviewer #1's Review

-In Figure 4 the interpretation of the results is not clear as the authors see some pri-miR-9 sometimes in the same cells and other times exclusive between different cells. What difference would that make for overall miR-9 expression in specific regions?

I agree with this comment. This figure should be presented in more convincing manner: count the number of positive cells for each pri-miR in the area and classify the expressing pattern according to colocalization for at least three different embryos.

We have performed new experiments and we have added two new figures (Figure 3 and 4) where the expression of pri-mir-9-1, 9-4, and 9-5 is characterised in more detail with triple smiFISH and the result is presented with absolute quantification. Figure 4 is devoted to the characterisation of co-localisation at 3 different time points. We hope that this addresses this criticism fully.

Reviewer #3's Review

FISH is more a qualitative than a quantitative approach, at the end it appears that almost all miR-9 genes are expressed simultaneously with the exception of form 1 and form 4-5, which are further detailed. It is therefore difficult to draw a certain conclusion on how much each isoform contributes, but it seems likely that all contribute in some way

The reviewer is correct in saying that it is indeed likely that each isoform contributes in some way and that all examined pri-mir-9s are indeed active and transcribed during embryonic development. However, in this paper we examine a specific hypothesis, which is that the mature miR-9 increases in single cells by the onset of expression of an additional pri-mir-9 locus during development in the same cells. This new concept is not mutually exclusive with the idea of tissue specific expression. The reviewer is also correct that the FISH analysis on Figure 1 is qualitative but it allowed us to select specific pri-mir-9s (9-1, 9-4, 9-5) for further detailed characterisation. In the revised manuscript we have supplemented the qualitative approach with quantitative smiFISH analysis.

-the single cell resolution analysis is limited to FISH and not really quantitative.

We have improved the single cell resolution analysis by adding triple smiFISH data for pri-mir-9-1, 9-4, and 9-5. smiFISH is very reliable in detecting the sites of transcription as ON or OFF because we are using probes that cover over 1kb of the primary transcript. The result is shown in new Figures 3 and 4 and is quantitated in Fig. 3D, E, F.

I agree with this comment. qPCR for each pri-miRs from the slice shown in Fig. 4 could be a more quantitative approach.

We have used Taqman RT-qPCR for absolute quantification of mature miR-9 and SYBR green RT-qPCR for the seven pri-mir-9s over the hindbrain region at 5 different time points presented as new data in Fig. 1.
We also performed triple smiFISH for the selected primary transcripts presented in new Figures 3 and 4, as mentioned above

-the characterization of the E or N boxes is rather superficial and has more of a preliminary rather than a mechanistic character. There is no validation that the number of binding sites actually contribute or determine to the expression pattern or that transcription factors such as Neurogenin are really involved

© 2022. Published by The Company of Biologists under the terms of the Creative Commons Attribution License (https://creativecommons.org/licenses/by/4.0/).

I agree. To obtain a clue to answer this question, I suggested to do luciferase assay.

We agree that this was a weak part of the paper. We have removed all of the E-box part of the data from the revised paper as it was not a key finding the paper and it detracted from main message. This allowed us to deepen and strengthen the rest of the data.

Reviewer 3

Evidence, reproducibility and clarity

The paper by Soto et al, deals with the expression and the regulation of miR-9 during zebrafish neurogenesis. Authors provide a detailed analysis of the spatiotempral profile of expression of the (7) different genes of miR-9 by fluorescent in situ hybridization in a time window between 24 and 48 hpf.
It emerged that some the pri-miR-9 transcripts are expressed in different brain area, in different cells or at different time. Therefore, the different miR-9 genes together contribute to define the spatio-temporal expression pattern of miR-9, and contribute incrementally to its accumulation over time during neurogenesis.

However there are some objective limits:

-the work relies exclusively on the FISH technique, which is very powerful to follow the expression of individual genes but should be corroborated with an independent approach

In the revised manuscript we have repeated and improved the expression experiments with triple smiFISH and RT-qPCR data, as explained above (Figures 1, 3 and 4).

-FISH is more a qualitative than a quantitative approach, at the end it appears that almost all miR-9 genes are expressed simultaneously with the exception of form 1 and form 4-5, which are further detailed. It is therefore difficult to draw a certain conclusion on how much each isoform contributes, but it seems likely that all contribute in some way

The reviewer is correct in saying that it is indeed likely that each isoform contributes in some way and that all pri-mir-9s examined are indeed active and transcribed during embryonic development. However, in this paper we examine a specific hypothesis, in which the mature miR-9 increases in single cells by the onset of expression of an additional pri-mir-9 locus during development in the same cells. This new concept is not mutually exclusive with the idea of tissue specific expression. The reviewer is also correct that the FISH analysis on Figure 1 is qualitative, but it allowed us to select specific pri-miR-9s (9-1, 9-4, 9-5) for further detailed characterisation. In the revised manuscript we have supplemented the qualitative approach with quantitative smiFISH analysis.

-the single cell resolution analysis is limited to FISH and not really quantitative

We have improved the single cell resolution analysis by adding triple smiFISH data for pri-mir-9-1, 9-4, and 9-5. smiFISH is very reliable in detecting the sites of transcription as ON or OFF because we are using probes that cover over 1kb of the primary transcript. The result is shown in new Figures 3 and 4 and is quantitated in Fig. 3D, E, F.

-the characterization of the E or N boxes is rather superficial and has more of a preliminary rather than a mechanistic character. There is no validation that the number of binding sites actually contribute or determine to the expression pattern or that transcription factors such as Neurogenin are really involved

We agree that this was a weak part of the paper. We have removed all of the E-box part of the data from the revised paper as it was not a key finding the paper and it detracted from main message. This allowed us to deepen and strengthen the rest of the data.

© 2022. Published by The Company of Biologists under the terms of the Creative Commons Attribution License (https://creativecommons.org/licenses/by/4.0/).

- There is no genetic/biochemical/molecular approach to mechanistic dissection of the involvement of various miR-9 genes and their contributions to total miR-9 levels or to regulation of target genes. This could help give a more mechanistic and less descriptive aspect to the work.

We agree with the reviewer that more mechanistic insight was needed in this paper. We have addressed this in two ways. First, we have added a CRISPR/Cas9 knock-down experiment of the late pri-mir-9-1, which we then analysed with a panel of neuronal markers. We found that this knock-down resulted in a specific reduction of late differentiating neurons. This experiment is shown in new Figure 5. Thus, we now show that the late increase of miR-9, contributed by the late pri-mir-9 paralogue, 9-1, is specifically needed to mediate late neurogenesis.

Second, we have used mathematical modelling to ask whether not only the increase of miR-9, but also the way by which the increase is achieved, may be functionally important. This was motivated by the RT-qPCR data for mature miR-9 in Figure 1 and for quantification of active transcription sites for pri-mir-9-1, 9-4 and 9-5 in Figure 6, which show that the increase during development takes place sharply, in steps. We used theory to predict what a network that shows adaptation to fluctuations in miR-9 would look like. This simple network is based on interactions that are currently known plus some hypothetical, predicted, components. Mathematical modelling then shows that this network shows adaptation of its output (her6 level) to linear increase or fluctuations of input (miR-9). Interestingly, the stepwise increase of miR-9 prevents adaptation of this network and results in persistent downregulation of her6, which is necessary for cells to differentiate. These new data are shown in Figure 6. We think this part of the work is particularly exciting because it reconciles the need of gene expression networks for robustness to external fluctuations, with the need to respond to changes in the input so that they can reach a new state which is particularly important for developmental transitions.

In addition to the results section, we have re-written the title, abstract and discussion to incorporate these new findings.

Significance

This work is technically excellent, but of limited significance.

The analysis of the expression of the various genes is very detailed, with excellent image quality and a precise description. However, significance is limited to the purely descriptive nature of the work.

No mechanistic hypothesis emerges. There is just an in silico correlation with E or N boxes, which at the end does not explain neither the pattern of expression of the different miR-9 genes, nor the different level of accumulation of the mature miR-9

*The whole work is actually a starting point for the subsequent dissection of the mechanisms of regulation of miR-9 expression **or** the dissection of the specific impact of different genes to miR-9 functions, which in my opinion would be of great interest.*

We hope that with the addition of functional and theoretical experiments, as detailed immediately above (and described in new Figures 5 and 6) fully addresses the criticism of lack of mechanistic insight. We hope that the paper can now be seen as having great interest and significance.

References

Amin, N.D., Senturk, G., Costaguta, G., Driscoll, S., O'Leary, B., Bonanomi, D., Pfaff, S.L., 2021. A hidden threshold in motor neuron gene networks revealed by modulation of miR-218 dose. Neuron 109, 3252-3267 e3256.

Goodfellow, M., Phillips, N.E., Manning, C., Galla, T., Papalopulu, N., 2014. microRNA input into a neural ultradian oscillator controls emergence and timing of alternative cell states. Nature communications 5, 3399.

© 2022. Published by The Company of Biologists under the terms of the Creative Commons Attribution License (https://creativecommons.org/licenses/by/4.0/).

Soto, X., Biga, V., Kursawe, J., Lea, R., Doostdar, P., Thomas, R., Papalopulu, N., 2020. Dynamic properties of noise and Her6 levels are optimized by miR-9, allowing the decoding of the Her6 oscillator. EMBO J 39, e103558.

Original submission

First decision letter

MS ID#: DEVELOP/2021/200474

MS TITLE: Timing of neurogenesis through sequential accumulation of miR-9 due to additive expression of multiple alleles

AUTHORS: Ximena Soto, Joshua Burton, Cerys S Manning, Thomas Minchington, Robert Lea, Jessica Lee, Jochen Kursawe, Magnus Rattray, and Nancy Papalopulu

Apologies for the delay in obtaining reports from the reviewers of your manuscript. I have now received two referee reports and have reached a decision. The referees' comments are appended below, or you can access them online: please go to BenchPress and click on the 'Manuscripts with Decisions' queue in the Author Area.

The overall evaluation is positive and we would like to publish a revised manuscript in Development, provided that the referees' comments can be satisfactorily addressed. As you will see, one referee's comments request quite minor changes whereas theothersuggests moresubstantialchanges and a shift in focus. I hope you find both sets of comments helpful in further revising the manuscript. Please attend to all of the reviewers' comments in your revised manuscript and detail them in your point-by-point response. If you do not agree with any of their criticisms or suggestions explain clearly why this is so.

Reviewer 1

Advance summary and potential significance to field

In their study entitled 'Timing of neurogenesis through sequential accumulation of miR-9 due to additive expression of multiple alleles', Soto et al show that 7 precursors of the micro-RNA miR-9 (pri-miR-9-1~7) have distinct spatial and temporal expression patterns. Moreover, they found that in the developing hindbrain, mature miR-9 increased in a sharp stepwise manner. From mathematical modelling, they speculated the benefits of a stepwise increase of miR-9 expression to overcome the adaptation mechanism maintaining steady state of expression profile.
The data and the model they presented in the revised version obviously appealing and convincing.

Comments for the author

1) Add discussion on the upstream factors regulating the expression of each pri-miR-9s that show temporally programmed manner.
2) The authors stated “we have shown by smiFISH that pri-mir-9-1, a late onset primary transcript, is co-expressed in the same cells as the earlier on” and “This co-expression may be a strategy to increase the amount of miR-9 available to the cell more than what would be possible with transcription from one locus alone”. pri-miR-9-1 showed broader expression area as compared to pri-miR-9-4 and 5 at later stages in Fig. 2C,D . The authors should discuss the possibility that this may be the main cause of Her6/9 reduction rather than stepwise- and additive expression of pre-miR-9s.
3) Discuss if the similar mechanism of stepwise increase of miR-9s (or other miRs) to overcome the adaptation mechanism can be seen in the mammalian brain development.
4) The authors should show the temporal and spatial expression of Her6/9 in either in situ or immunohistochemistry in the miR-9-1 KO mouse to validate their hypothesis.

© 2022. Published by The Company of Biologists under the terms of the Creative Commons Attribution License (https://creativecommons.org/licenses/by/4.0/).

5) The authors stated “We suggest that in the case of miR-9, a sharp, non-linear, increase may be needed to push a dynamical system into a new state and this may be associated with a cell fate change’.
The authors should discuss if they observed anything that support their idea from miR-9-1 ko brain.

Reviewer 2

Advance summary and potential significance to field

The paper by Soto et al. deals with the expression and the regulation of miR-9 during zebrafish neurogenesis.
Authors provide a detailed analysis of the spatio-tempral profile of expression of the (7) different genes of miR-9 by fluorescent in situ hybridization in a time window between 24 and 48 hpf. It emerged that some the pri-miR-9 transcripts are expressed in different brain area, in different cells or at different time. Single-Molecule FISH analyses reveal that the expression of pri-miR-9-1 at late stage of differentiation is added on to pri-miR-9-3/5 which are expressed at early stage. Using CRISPR/cas9 mediated genome engineering authors generated mutant animals in which the late onset of miR-9 was prevented by removing the pri-miR-9-1 hairpin, resulting in altered expression of neuronal differentiation markers. These results suggest a fine and incremental regulation of miR-9 quantities during neurogenesis.

Comments for the author

This work is technically very well done. The analysis of the expression of the various genes is detailed, with excellent image quality and a precise description of the procedure and results. Although the significance of the work is limited to its purely descriptive nature , the technical value and the precision in the characterization and quantitation of the expression of miR-9 genes during animal neurogenesis is high and can be considered a sufficient contribution to the field.
In order to provide explanation on the significance of the incremental regulation of miR-9, authors discuss a mathematical model centered on the interaction of miR-9 with the Her6 target. However, this part has a very limited significance as it is mostly theoretical and based on non-totally quantitative data. Furthermore the interaction with the Her6 target is part of a previous work by the same authors. Therefore, I would remove from the paper this part and keep the focus on the transcriptional characterization
Points to be addressed are:
POINT 1.
To reinforce the quantitative nature of miR-9 expression characterization , it would be helpful if the authors measure absolute miR-9 expression levels. This could be achieved with a calibration curve using exact quantities of synthetic miR-9 and then converting qPCR data into copies normalized per mass unit (i.e. microgram) or per cell (or per animal, here).
Examples are Figure 1B, Figure 1D, Figure 1E
POINT 2.
I would suggest to strengthen the characterization of the phenotype upon mutation of the pri-miR-9-1 hairpin, which abrogate the late miR-9 onset.
The current characterization is limited to the analysis of expression (by qPCR) of some neurogenesis markers, with only a few showing dysregulation and no overt abnormality mentioned. Her6/9 expression was not mentioned neither analyzed (i was curious to see if changed). Some immunophenotypic characterization is needed here, in particular if authors wish to conclude that "increase in miR-9 in development is functionally important for differentiation” (line 310 and abstract)

© 2022. Published by The Company of Biologists under the terms of the Creative Commons Attribution License (https://creativecommons.org/licenses/by/4.0/).

First revision

Author response to reviewers' comments

We would like to thank the reviewers for the care by which they have read and reviewed our paper and for their constructive comments. In this point-point by response, we outline how we have addressed their comments.

Reviewer 1 Advance Summary and Potential Significance to Field:
In their study entitled 'Timing of neurogenesis through sequential accumulation of miR-9 due to additive expression of multiple alleles', Soto et al show that 7 precursors of the micro-RNA miR-9 (pri-miR-9-1~7) have distinct spatial and temporal expression patterns. Moreover, they found that in the developing hindbrain, mature miR-9 increased in a sharp stepwise manner. From mathematical modelling, they speculated the benefits of a stepwise increase of miR-9 expression to overcome the adaptation mechanism maintaining steady state of expression profile. The data and the model they presented in the revised version obviously appealing and convincing.

Thank you for finding our work appealing and convincing.

Reviewer 1 Comments for the Author:
1)Add discussion on the upstream factors regulating the expression of each pri-miR-9s that show temporally programmed manner.

Unfortunately, this information is not known. In fact, our paper is the first step in understanding the regulation of each pri-miR-9s and historically, it was the motivation for doing this work.

2)The authors stated "we have shown by smiFISH that pri-mir-9-1, a late onset primary transcript, is co-expressed in the same cells as the earlier on" and "This co-expression may be a strategy to increase the amount of miR-9 available to the cell more than what would be possible with transcription from one locus alone". pri-miR-9-1 showed broader expression area as compared to pri-miR-9-4 and 5 at later stages in Fig. 2C,D . The authors should discuss the possibility that this may be the main cause of Her6/9 reduction rather than stepwise- and additive expression of pre-miR-9s.

Yes, it is true that miR-9-1 may be expressed in cells that do not express the other two primary transcripts. We do not exclude tissue specific or domain specific effects of different pri-miR-9s that may target different genes in different areas or contribute to the suppression of a gene (like her6) outside its normal expression domain. However, our single cell imaging analysis allowed us to identify cells that definitively co-express the late pri-miR-9-1 with the early pri-miR-9-4 and-5; it is in these cells that we propose that pri-miR-9s may have an additive temporal effect. We have added a point that we cannot exclude tissue or domain specific effects that may operate with the novel mechanism we propose here.

3)Discuss if the similar mechanism of stepwise increase of miR-9s (or other miRs) to overcome the adaptation mechanism can be seen in the mammalian brain development.

We are the first to propose this mechanism and we are not aware of any report of similar stepwise increase in the mammalian brain. However, in both mouse and humans there are 3 distinct primary miR-9s that are capable of producing the same mature miR-9 form, so it is not unrealistic to expect a similar mechanism to occur in mammals. There is some evidence that combinatorial KO of miR-196 paralogues have additive phenotypes in vertebral development and this is mentioned in page 19. We have clarified that this study was done in the mouse. We have added a point about this in at the end of the discussion.

4)The authors should show the temporal and spatial expression of Her6/9 in either in situ or immunohistochemistry in the miR-9-1 KO mouse to validate their hypothesis.

This is a good suggestion, and it constitutes our main experimental revision. We assume that the reviewer means miR-9-1 KO zebrafish, not mouse. To address this point, we analysed the expression of Her6 in pri-miR-9-1 KO zebrafish, as the reviewer suggests. To obtain quantitative measurements

© 2022. Published by The Company of Biologists under the terms of the Creative Commons Attribution License (https://creativecommons.org/licenses/by/4.0/).

we have used smiFISH for her6 in transverse sections of hindbrains from homozygous pri-miR-9-1 knock out embryos. We confirmed the findings with whole mount chromogenic ISH in homozygous embryos, as well as with snapshot fluorescent imaging in heterozygous pri-miR-9-1 knock out embryos, using the Her6: Venus knock-in line. In all cases, we observed a subtle but reproducible spatial increase of her6 at a time when it should be downregulated. This supports our hypothesis that the late her6 expression transition to a lower expression level does not take place in the absence of a late miR-9 input at the appropriate time and space during development. This is now in Fig. 7, Fig. S7 and separate section at the end of the results section.

5)The authors stated "We suggest that in the case of miR-9, a sharp, non-linear, increase may be needed to push a dynamical system into a new state and this may be associated with a cell fate change'. The authors should discuss if they observed anything that support their idea from miR-9-1 ko brain.

As we detail above, our idea is supported by the lack of her6 downregulation in late stages when pri-miR-9-1 is specifically knocked out.

Reviewer 2 Advance Summary and Potential Significance to Field: The paper by Soto et al. deals with the expression and the regulation of miR-9 during zebrafish neurogenesis. Authors provide a detailed analysis of the spatio-tempral profile of expression of the (7) different genes of miR-9 by fluorescent in situ hybridization in a time window between 24 and 48 hpf. It emerged that some the pri-miR-9 transcripts are expressed in different brain area, in different cells or at different time. Single-Molecule FISH analyses reveal that the expression of pri-miR-9-1 at late stage of differentiation is added on to pri-miR-9-3/5 which are expressed at early stage. Using CRISPR/cas9 mediated genome engineering, authors generated mutant animals in which the late onset of miR-9 was prevented by removing the pri-miR-9-1 hairpin, resulting in altered expression of neuronal differentiation markers. These results suggest a fine and incremental regulation of miR-9 quantities during neurogenesis.

Reviewer 2 Comments for the Author:This work is technically very well done. The analysis of the expression of the various genes is detailed, with excellent image quality and a precise description of the procedure and results. Although the significance of the work is limited to its purely descriptive nature, the technical value and the precision in the characterization and quantitation of the expression of miR-9 genes during animal neurogenesis is high and can be considered a sufficient contribution to the field.

We thank the reviewer for their supportive comments.

In order to provide explanation on the significance of the incremental regulation of miR-9, authors discuss a mathematical model centered on the interaction of miR-9 with the Her6 target. However, this part has a very limited significance as it is mostly theoretical and based on non-totally quantitative data. Furthermore the interaction with the Her6 target is part of a previous work by the same authors. Therefore, I would remove from the paper this part and keep the focus on the transcriptional characterization

We respectfully disagree with the reviewer here. The mathematical modelling was instrumental in helping us understand the significance of the stepwise miR-9 increase in the transcriptional or posttranscriptional response of the miR-9 target, her6. It served as a hypothesis generating tool, which allowed us to predict that without a stepwise increase, the expression of Her6 would not change because it would adapt to the linear increase of miR-9.
In the revised manuscript, we have tested this hypothesis by characterising the response of Her6 to the pri-mir-9-1 KO. The mathematical modelling also made a specific prediction that there exists a factor X that is targeted by miR-9 and targets Her6. In our preliminary bioinformatic analysis, we have identified Onecut as a candidate for factor X, which further supports the value of the model. This is very exciting as Onecut has been postulated to be a temporal neurogenesis factor and it will be followed up in future work. This has been added in the discussion (page 20).

© 2022. Published by The Company of Biologists under the terms of the Creative Commons Attribution License (https://creativecommons.org/licenses/by/4.0/).

Points to be addressed are:

POINT 1.
To reinforce the quantitative nature of miR-9 expression characterization, it would be helpful if the authors measure absolute miR-9 expression levels. This could be achieved with a calibration curve using exact quantities of synthetic miR-9 and then converting qPCR data into copies normalized per mass unit (i.e. microgram) or per cell (or per animal, here). Examples are Figure 1B, Figure 1D, Figure 1E.

As the reviewer says, we have done quantitative measuring of mature and each pri-mir-9, over development, by qPCR in Figure 1B, 1D and 1E. The reviewer suggests that, in addition, we should provide the copy number of the mature miR-9 by using a calibration curve of mature miR-9. Although this may be technically feasible, we have not done it because we think that the normalisation step will lead to uninterpretable results. We are taking the view that population averaging across multiple cells and embryos masks differences and dynamics that are evident at the single cell level. For this reason, most of our analysis is done by single cell imaging. It is currently not possible to provide a reliable estimate of microRNA copy number by imaging because they are too small to be reliably detected with sm or smiFISH- we have tried!

POINT 2.
I would suggest to strengthen the characterization of the phenotype upon mutation of the pri-miR-9-1 hairpin, which abrogate the late miR-9 onset. The current characterization is limited to the analysis of expression (by qPCR) of some neurogenesis markers, with only a few showing dysregulation and no overt abnormality mentioned. Her6/9 expression was not mentioned neither analyzed (i was curious to see if changed). Some immunophenotypic characterization is needed here, in particular if authors wish to conclude that "increase in miR-9 in development is functionally important for differentiation" (line 310 and abstract)

Yes, we agree that this was important to do. In the revised manuscript we analysed the expression of Her6/9 in pri-miR-9-1 KO zebrafish, as the reviewer suggests. To obtain quantitative measurements we have used smiFISH for *her6* in a homozygous pri-miR-9-1 knock down embryos, in transverse hindbrain sections. We confirmed the findings with whole mount chromogenic ISH in homozygous pri-miR-9-1 mutant embryos, as well as with snapshot fluorescent imaging in heterozygous pri-miR-9-1 mutant embryos. In all cases, we observed a subtle but reproducible spatial and temporal increase of her6 at a time when it should be downregulated. This supports our hypothesis that the late her6 expression transition to a lower expression level does not take place in the absence of a late miR-9 input. These findings are now shown in Fig. 7, Fig. S7, mentioned in the abstract, introduction (page 6) and new results section in page 16. The material and methods have correspondingly been expanded.

<u>Second decision letter</u>

MS ID#: DEVELOP/2021/200474

MS TITLE: Timing of neurogenesis through sequential accumulation of miR-9 due to additive expression of multiple alleles

AUTHORS: Ximena Soto, Joshua Burton, Cerys S Manning, Thomas Minchington, Robert Lea, Jessica Lee, Jochen Kursawe, Magnus Rattray, and Nancy Papalopulu

Apologies for the delay in obtaining the reviewers reports on your revised manuscript. However I have now received the reports and have reached a decision. The referees' comments are appended below, or you can access them online: please go to BenchPress and click on the 'Manuscripts with Decisions' queue in the Author Area.

As you will see, one reviewer is happy with the revisions but the other thinks that there are still two issues that have not been fully addressed in the revision. I think the referee suggestions may

© 2022. Published by The Company of Biologists under the terms of the Creative Commons Attribution License (https://creativecommons.org/licenses/by/4.0/).

indeed strengthen your manuscript but I will leave it to you to decide if you wish to do any further experimental work before we proceed to publication.

Please attend to all of the reviewers' comments in your revised manuscript and detail them in your point-by-point response. If you do not agree with any of their criticisms or suggestions explain clearly why this is so.

Reviewer 1

Advance summary and potential significance to field

In their study entitled 'Timing of neurogenesis through sequential accumulation 2 of miR-9 due to additive expression of multiple alleles', Soto et al show that 7 precursors of the micro-RNA miR-9 (pri-miR-9-1~7) have distinct spatial and temporal expression patterns. Moreover, they found that in the developing hindbrain, mature miR-9 increased in a sharp stepwise manner. From mathematical modeling, they speculated the benefits of a stepwise increase of miR-9 expression to overcome the adaptation mechanism maintaining a steady state of expression profile. With the newly added data on the expression of her6 in miR-9 KO, their study presented in the revised version is obviously convincing and deserves publication.

Comments for the author

2)The authors stated "we have shown by smiFISH that pri-mir-9-1, a late onset primary transcript, is co-expressed in the same cells as the earlier on" and "This co-expression may be a strategy to increase the amount of miR-9 available to the cell more than what would be possible with transcription from one locus alone". pri-miR-9-1 showed broader expression area as compared to pri-miR-9-4 and 5 at later stages in Fig. 2C,D . The authors should discuss the possibility that this may be the main cause of Her6/9 reduction rather than stepwise- and additive expression of pre-miR-9s.
Yes, it is true that miR-9-1 may be expressed in cells that do not express the other two primary transcripts. We do not exclude tissue specific or domain specific effects of different pri-miR-9s that may target different genes in different areas or contribute to the suppression of a gene (like her6) outside its normal expression domain. However, our single cell imaging analysis allowed us to identify cells that definitively co-express the late pri-miR-9-1 with the early pri-miR-9-4 and-5; it is in these cells that we propose that pri-miR-9s may have an additive temporal effect.
We have added a point that we cannot exclude tissue or domain specific effects that may operate with the novel mechanism we propose here.
In the revised version, they discuss this point quite well.
3) Discuss if the similar mechanism of stepwise increase of miR-9s (or other miRs) to overcome the adaptation mechanism can be seen in the mammalian brain development.
We are the first to propose this mechanism and we are not aware of any report of similar stepwise increase in the mammalian brain. However, in both mouse and humans there are 3 distinct primary miR-9s that are capable of producing the same mature miR-9 form, so it is not unrealistic to expect a similar mechanism to occur in mammals. There is some evidence that combinatorial KO of miR-196 paralogues have additive phenotypes in vertebral development and this is mentioned in page 19. We have clarified that this study was done in the mouse. We have added a point about this in at the end of the discussion.
In the revised version, they discuss this point quite well.
4)The authors should show the temporal and spatial expression of Her6/9 in either in situ or immunohistochemistry in the miR-9-1 KO mouse to validate their hypothesis.
5)The authors stated "We suggest that in the case of miR-9, a sharp, non-linear increase may be needed to push a dynamical system into a new state and this may be associated with a cell fate change'. The authors should discuss if they observed anything that supports their idea from miR-9-1 ko brain.
This is a good suggestion, and it constitutes our main experimental revision.
We assume that the reviewer means miR-9-1 KO zebrafish, not mouse.
To address this point, we analysed the expression of Her6 in pri-miR-9-1 KO zebrafish, as the reviewer suggests.

© 2022. Published by The Company of Biologists under the terms of the Creative Commons Attribution License (https://creativecommons.org/licenses/by/4.0/).

To obtain quantitative measurements we have used smiFISH for her6 in transverse sections of hindbrains from homozygous pri-miR-9-1 knock out embryos.
We confirmed the findings with whole mount chromogenic ISH in homozygous embryos, as well as with snapshot fluorescent imaging in heterozygous pri-miR-9-1 knock out embryos, using the Her6: Venus knock-in line.
In all cases, we observed a subtle but reproducible spatial increase of her6 at a time when it should be downregulated. This supports our hypothesis that the late her6 expression transition to a lower expression level does not take place in the absence of a late miR-9 input at the appropriate time and space during development.
This is now in Fig. 7, Fig. S7 and a separate section at the end of the results section.
done in the mouse. We have added a point about this in at the end of the discussion.
As we detail above, our idea is supported by the lack of her6 downregulation in late stages when pri-miR-9-1 is specifically knocked out.
This is the main point that I think improves the manuscript greatly. The authors added a detailed expression pattern analysis of her6 in miR-9-1-/- KO, which satisfactorily answers my question.

Reviewer 2

Advance summary and potential significance to field

This work is technically strong. The expression analysis of various miRNA genes is detailed, with excellent image quality and precise description. The characterization and quantification of miR-9 gene expression during animal neurogenesis is high and can be considered a sufficient contribution to the field.
At this stage the document still needs some improvements

Comments for the author

The manuscript has been revised. However, there are still some points that have not been fully solved.
POINT 1. We have asked for more quantitative assessment of miR-9 expression, which has been performed so far with qPCR in Figure 1B, Figure 1D, Figure 1E. However, authors, refused and they motivated with “Although this may be technically feasible, we have not done it because we think that the normalisation step will lead to uninterpretable results.”…The meaning of “uninterpretable results” is unclear as the results would not be much different that those from a regular qPCR, as those in Figure 1B, Figure 1D, Figure 1E, but much more accurate. The normalization has been suggested as copies per microgram of RNA or copies per cell, hence this motivation cannot be accepted. In addition, absolute quantitation is not hard to achieve, as admitted by authors…
POINT 2. We have asked for improving the characterization of the phenotype upon mutation of the pri-miR-9-1 hairpin, which abrogate the late miR-9 onset. The same request came also from the other reviewer.
However, authors have just performed smiFISH analysis for her6, which is shown in Figure 7, with one representative image and 6 total measurements (control and KO), which in my opinion is insufficient.

Second revision

Author response to reviewers' comments

We were happy to see that reviewer 1 found that we have addressed their comments satisfactorily and no further work is requested.

Reviewer 2 feels that the manuscript can be improved further. With regards to the request for absolute quantification of copies of microRNA per cell, we still think that this experiment will not add much to the paper, considering that we have performed RT-qPCR both of the mature mir_9 and each of the primary transcripts and these are shown in several places in the manuscript (as the

© 2022. Published by The Company of Biologists under the terms of the Creative Commons Attribution License (https://creativecommons.org/licenses/by/4.0/).

reviewer notes). Perhaps we did not explain well what we mean by the normalisation step being problematic and for this we apologise. What we mean is that, dividing the number of microRNA copies with the number of cells would lead to an average abundance (“population averaging”) which would be potentially quite inaccurate as it does not account for cell to cell heterogeneity. It is also unclear how we would decide the number of cells in the denominator. For these reasons, coupled with a lack of strong justification of what extra useful information it would provide to the reader, we decided not to do this experiment.
With regards to the second point, which is the characterisation of phenotype upon mutation of pri-mir-9-1, the original manuscript already showed the results of the analysis of several neuronal markers in Fig. 5. Further on, the revised manuscript added a characterisation of the effect of pri-miR-9-1 mutation on the expression of her6. This experiment was performed by crossing Her6::Venus+/-;pri-mir-9-1+/- F1 adults, followed by live imaging of the endogenous fluorescently tagged her6 in small pools of embryos which was then followed by genotyping, so that homozygous and heterozygous mutants can be compared to controls. In the current revision, we have repeated the experiment 3 more times which allowed to increase the biological repeats and sample size, as shown in Fig. S7.

We have reduced the abstract to the stated limit of 180 words and we have slightly changed the title to make it snappier.

Third decision letter

MS ID#: DEVELOP/2021/200474

MS TITLE: Sequential and Additive Expression of miR-9 precursors Control Timing of Neurogenesis

AUTHORS: Ximena Soto, Joshua Burton, Cerys S Manning, Thomas Minchington, Robert Lea, Jessica Lee, Jochen Kursawe, Magnus Rattray, and Nancy Papalopulu
ARTICLE TYPE: Research Article

Thank you for sending your manuscript to Development through Review Commons.

I am happy to tell you that your manuscript has been accepted for publication in Development, pending our standard ethics checks.

© 2022. Published by The Company of Biologists under the terms of the Creative Commons Attribution License (https://creativecommons.org/licenses/by/4.0/).
